# Transition-metal-free [3 + 3] annulation of indol-2-ylmethyl carbanions to nitroarenes. A novel synthesis of indolo[3,2-*b*]quinolines (quindolines)

**DOI:** 10.3762/bjoc.14.14

**Published:** 2018-01-23

**Authors:** Michał Nowacki, Krzysztof Wojciechowski

**Affiliations:** 1 Institute of Organic Chemistry, Polish Academy of Sciences, Kasprzaka 44/52, 01-224 Warsaw, Poland

**Keywords:** carbanions, cyclization, heterocycles, nitroarenes, nucleophilic substitution, silylation

## Abstract

Indol-2-ylmethyl carbanions stabilized by alkoxycarbonyl, cyano or benzenesulfonyl groups react with nitroarenes to form σ^H^-adducts, which in the presence of base (triethylamine or DBU) and trimethylchlorosilane transform into indolo[3,2-*b*]quinoline derivatives in moderate to good yields.

## Introduction

**Figure 1:**
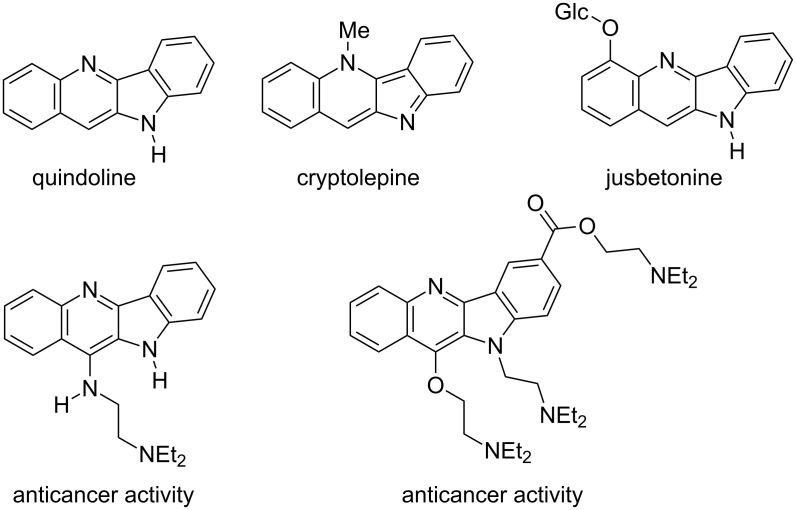
Selected indolo[3,2-*b*]quinolines (quindolines) with biological activity.

The indolo[3,2-*b*]quinoline (quindoline) system is present in numerous alkaloids of plant origin representing several biological activities and used in traditional tropical medicine for the treatment of various diseases, particularly malaria [1–5]. Further development revealed the potential of synthetic quindoline derivatives as anticancer agents [1,6–7] (Figure 1).

**Scheme 1:**
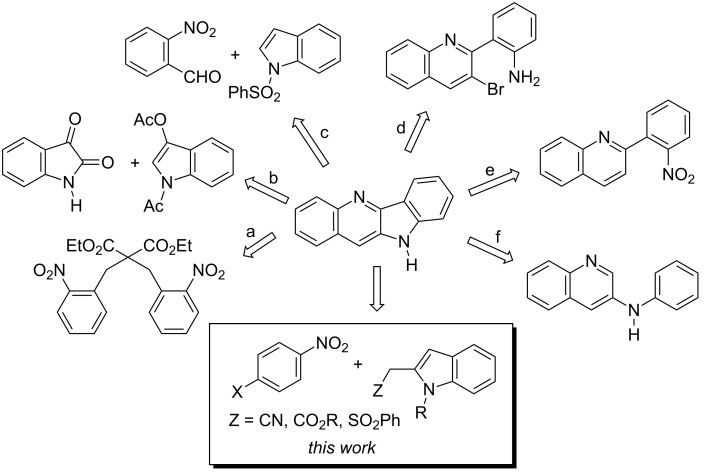
Selected starting materials for the construction of the quindoline system.

Synthetic strategies towards indolo[3,2-*b*]quinolines have been reviewed [8]. These methodologies usually employ multistep procedures. Selected starting materials applicable to the synthesis of indolo[3,2-*b*]quinolines are presented in Scheme 1.

Bis(2-nitrobenzyl)malonate upon treatment with sodium hydroxide undergoes a multistep transformation resulting in 11-hydroxyindolo[3,2-*b*]quinoline-5-*N*-oxide (a) [9]. This approach is based on the first synthesis of the indolo[3,2-*b*]quinoline framework by Fichter and Boehringer in 1906 [10]. Indoxyl [11] and *N*,*O*-diacetylindoxyl [12] under basic conditions condensed with isatine to form quindoline-11-carboxylic acid easily transformed into quindoline (b) [12]. Another application of indole derivatives to the construction of quindoline starts with the reaction of 1-phenylsulfonylindole with 2-nitrobenzaldehyde (c) [13]. Several methods use prerequisite quinoline derivatives for the construction of the pyrrole ring of quindoline. 2-(2-Aminophenyl)-3-bromoquinoline cyclized to quindoline by reacting with pyridinium hydrochloride (d) [14]. Insertion of nitrene generated from 2-(2-nitrophenyl)quinoline by triethylphosphite (e) [15] and palladium(II) acetate-mediated oxidative cyclization of 3-(phenylamino)quinoline (f) afford quindoline [16]. Some of these syntheses require harsh conditions and multistep procedures. Another drawback of some of the known methodologies is the use of transition metal catalysts for the formation of prerequisite reagents or for ultimate steps of the synthesis resulting in the formation of the indolo[3,2-*b*]quinoline framework [14,16–18]. These procedures may require meticulous removal of traces of transition metals, particularly if the product is intended to use in biomedical or pharmaceutical applications [19].

**Scheme 2:**
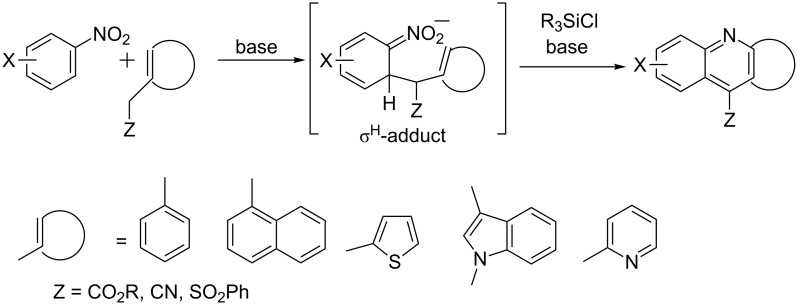
Synthesis of condensed pyridines mediated by a σ^H^-adduct.

During our studies on the reactions of nitroarenes with nucleophiles [20–23], particularly carbanions, we were interested in a direct transformation of the formed σ^H^-adducts into heterocyclic systems – indoles [21,23] and quinolines [21–22]. Quinolines were formed when the σ^H^-adducts, obtained from an allyl sulfone carbanion, were treated with a silylating agent or a Lewis acid [24]. In a similar reaction σ^H^-adducts of benzyl carbanions of arylacetonitriles upon treatment with trialkylchlorosilanes yielded 9-cyanoacridines [25]. This [3 + 3] annulation leading to quinoline can be considered as an umpolung of the Skraup quinoline synthesis since it uses reversed polarity of reagents, where the C=C–NO_2_ fragment of the nitroarene is the electrophile reacting with an arylmethyl carbanion, the nucleophile (Scheme 2). Recently, we used the reactions of heteroanalogues of benzyl carbanions to synthesise 11-substituted norcryptotackieine derivatives (indolo[2,3-*b*]quinolines) [26], benzothieno[2,3-*b*]quinolines [27], and other tetra- and pentacyclic polyazaarenes containing the quinoline system [28].

We elaborated two general variations of this methodology. In the first one, both reagents, C–H acid and nitroarene, upon treatment with a relatively weak base such as DBU and a silylating agent or Lewis acid, undergo slow transformation to the quinoline derivative. In this process, a low concentration of the σ^H^-adduct is postulated. Another approach uses a strong base such as potassium *tert*-butoxide to form the σ^H^-adduct in high concentration. This σ^H^-adduct is then treated with a silylating agent and a weaker base to form the final product.

## Results and Discussion

In this paper, we present the transformation of σ^H^-adducts, formed from indol-2-ylmethyl carbanions and nitroarenes, to indolo[3,2-*b*]quinoline derivatives. As starting nucleophile precursors, we have chosen derivatives of indole-2-ylmethyl phenyl sulfone (**1a**), *tert*-butyl indol-2-ylacetate (**1b**,**c**) and indol-2-ylacetonitrile (**1d**).

As a model compound for screening of the reaction conditions, we used sulfone **1a**. We found that no reaction occurred when the sulfone and 4-chloronitrobenzene were treated with DBU and trimethylchlorosilane. Even when we kept these reagents for a prolonged time (up to six days) no product was observed and the starting materials were recovered. When we treated these reagents in tetrahydrofuran solution containing triethylamine precooled to −70 ºC with *t*-BuOK and after 20 min added trimethylchlorosilane, stirred for 3 h at −65 to −60 °C, and then allowed to reach room temperature and stirred for 24 h we obtained 10-(*n*-butyl)-11-benzenesulfonyl-10*H*-indolo[3,2-*b*]quinoline **3** in 59% yield.

Under these conditions other nitrobenzene derivatives **2a–e** reacted similarly, giving the condensed azaarenes (**4–9**) in good yields. The results are summarized in Table 1.

**Table 1:** The synthesis of indolo[3,2-*b*]quinolines (step-by-step procedure).

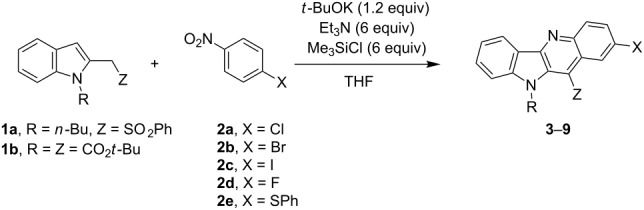			

Indole	ArNO_2_	Product	Yield (%)

**1a**	**2a**	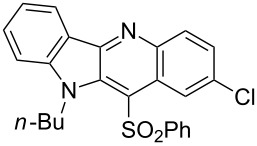 **3**	59
**1a**	**2b**	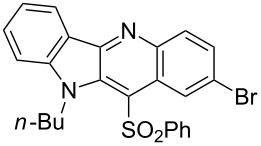 **4**	59
**1a**	**2c**	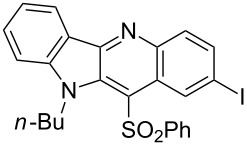 **5**	33
**1a**	**2d**	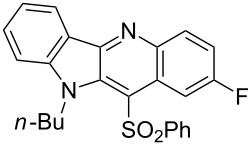 **6**	15
**1a**	**2e**	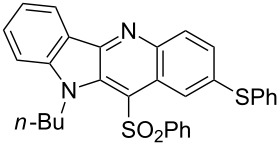 **7**	34
**1b**	**2a**	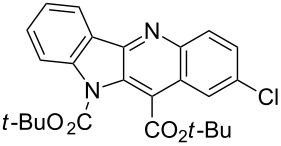 **8**	28
**1b**	**2b**	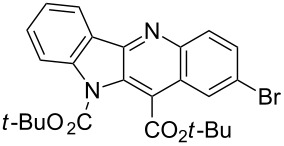 **9**	22

**Scheme 3:**
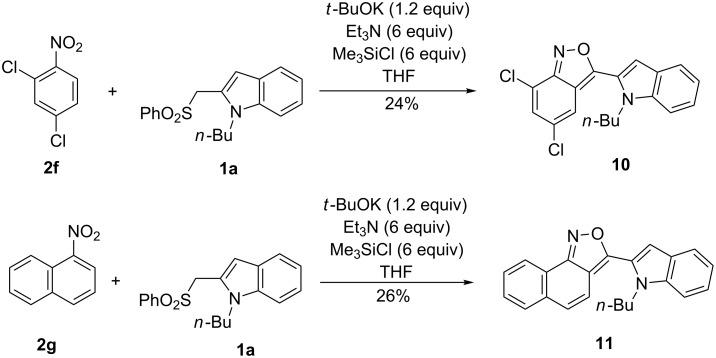
Formation of condensed isoxazole derivatives.

In some cases, we observed in the reaction of sulfone **1a** the formation of other products. This occurred in the reaction with 2,4-dichloronitrobenzene (**2f**), where we obtained 3-(indol-2-yl)-2,1-benzisoxazole **10** instead of the expected indoloquinoline. In an analogous reaction with 1-nitronaphthalene (**2g**) condensed isoxazole derivative **11** was formed (Scheme 3). The formation of 2,1-benzisoxazoles in reactions of benzyl sulfone carbanions with nitroarenes under similar conditions has been already observed [29].

Reactions of *tert*-butyl 1-(*tert*-butoxycarbonyl)indol-2-ylacetate (**1b**) with *p*-chloro- and *p*-bromonitrobenzene led to the expected products **8** and **9** in moderate yields.

**Scheme 4:**
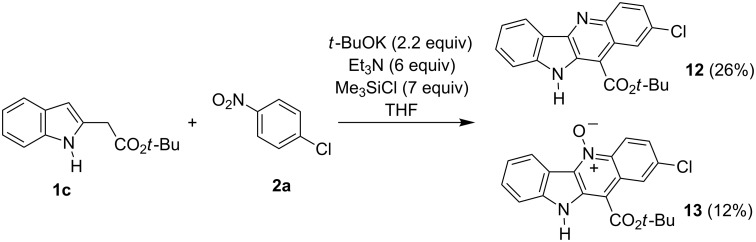
Reaction of unprotected indole ester **1c** with 4-chloronitrobenzene.

In one of our previous papers [28] we used *N*-silylated indol-3-ylacetonitrile as a carbanion precursor in the reaction with nitroarenes. Attempts to obtain *tert-*butyl *N*-trimethylsilylindol-2-ylacetate via direct silylation of the ester **1c** with trimethylchlorosilane was unsuccessful, the product proved unstable and decomposed rapidly. Thus, we decided to perform a one-pot reaction in which in situ *N*-silylation furnished the expected carbanion precursor. Thus, when we treated *tert*-butyl indol-2-ylacetate (**1c**) and *p*-chloronitrobenzene (**2a**) with triethylamine and potassium *tert*-butoxide and then added trimethylchlorosilane we obtained unprotected indoloquinoline **12** and its *N*-oxide **13** in 26 and 12% yields, respectively (Scheme 4).

To extend the scope of the reaction to more active nitroarenes, we used slightly modified reaction conditions using DBU as a base. Earlier we have found that in the reactions of bicyclic nitroarenes this base was sufficiently strong to deprotonate ethyl *N*-(*tert*-butoxycarbonyl)indol-3-ylacetate [28].

Thus, when we mixed *tert*-butyl *N*-(*tert*-butoxycarbonyl)indol-2-ylacetate (**1b**), 5-nitroquinoline (**2i**), trimethylchlorosilane and DBU in dimethylformamide at room temperature we found that after four days both starting materials disappeared (TLC control) and we isolated indolophenanthridine **16** in 41% yield. Other nitroarenes under analogous reaction conditions gave condensed indole derivatives as summarized in Table 2. Better yields were obtained in the reactions of 1-(*tert*-butoxycarbonyl)indol-2-ylacetonitrile with nitroquinolines **2i–k** and 5-nitroindazole **2l**. This can be attributed to a lesser sterical demand in the formation of the σ^H^-adduct by a carbanion stabilized by the cyano group in comparison with the ester group.

**Table 2:** The synthesis of indolo[3,2-*b*]quinolines by one-pot procedure.

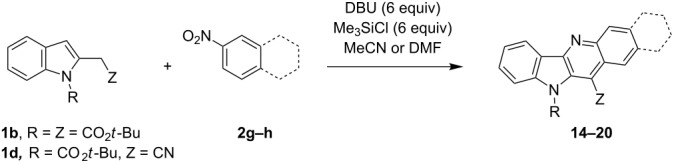					

Indole	ArNO_2_	Product	Solvent	Time [days]	Yield (%)

**1b**	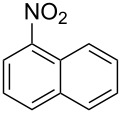 **2g**	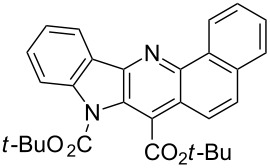 **14**	MeCN	11	18
**1b**	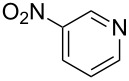 **2h**	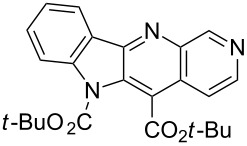 **15**	MeCN	10	40
**1b**	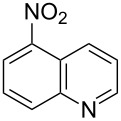 **2i**	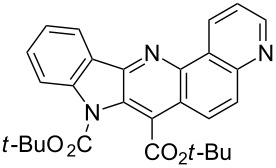 **16**	DMF	4	41
**1b**	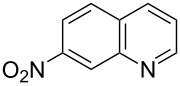 **2j**	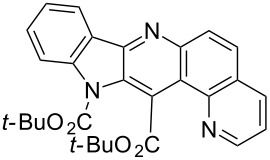 **17**	DMF	7	13
**1d**	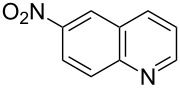 **2k**	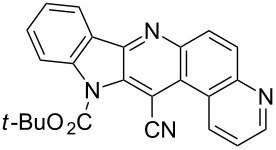 **18**	DMF	2	72
**1d**	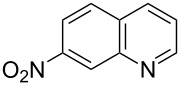 **2j**	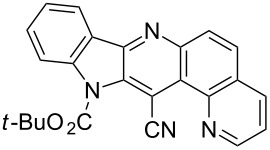 **19**	DMF	3	51
**1d**	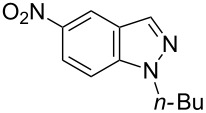 **2l**	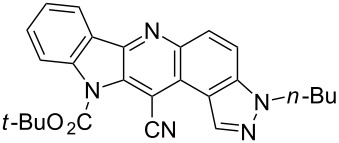 **20**	MeCN	4	61

**Scheme 5:**
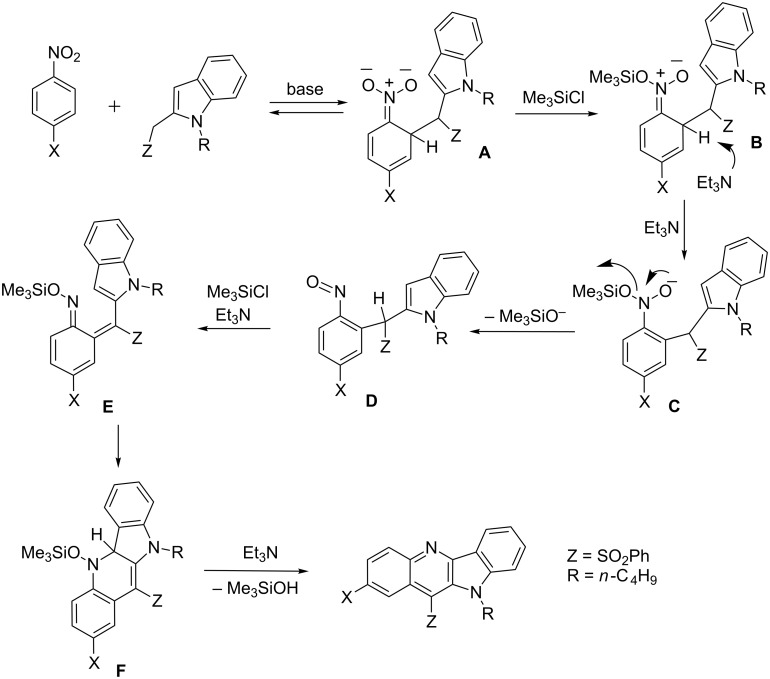
A plausible mechanism for the formation of 11-(phenylsulfonyl)indolo[3,2-*b*]quinolines.

A plausible way for the formation of indolo[3,2-*b*]quinolines from σ^H^-adduct **A** generated from the indolylmethyl carbanion of the sulfone and 4-substituted nitrobenzenes is shown in Scheme 5. The formed σ^H^-adduct **A** undergoes *O*-silylation and the formed silyl nitronate **B** is deprotonated to form intermediate **C**, that, after elimination of silanol, forms nitroso intermediate **D**. Consecutive silylation of **D** leads to azaxylylene **E** that electrocyclizes to the dihydroquinoline framework **F** from which, after elimination of another molecule of silanol, the final product is formed. Similar electrocyclizations of aza-*ortho*-xylylenes leading to condensed heterocycles are known processes [30–31].

## Conclusion

We developed a simple synthesis of indolo[3,2-*b*]quinoline derivatives by the reaction of nitroarenes with indol-2-ylmethyl carbanions generated from easily accessible starting materials. The reactions proceed under mild conditions and the elaborated method does not employ transition metals at any stage thus might be applicable in the synthesis of pharmaceutically important intermediates.

## Experimental

Melting points are uncorrected. ^1^H and ^13^C NMR spectra were recorded on Bruker Avance 500 or Varian vnmr s500 (both 500 MHz for ^1^H and 125 MHz for ^13^C spectra) instruments at 298 K. Chemical shifts are expressed in parts per million (ppm) referred to TMS, coupling constants in hertz (Hz). Electron impact mass spectra (EI, 70 eV) were obtained on an AutoSpec Premier spectrometer. Electrospray mass spectra (ESI) were obtained on 4000 Q-TRAP and SYNAPT G2-S HDMS. Silica gel (Merck 60, 230–400 mesh) was used for column chromatography (CC). Toluene or hexane/ethyl acetate mixtures were used for elution. TLC analyses were performed on Merck silica lgel 60 F_254_ aluminum plates with hexane/ethyl acetate mixtures. All reagents and solvents were of reagent grade or purified according to standard methods before use. All reactions were run under argon atmosphere.

**Reactions of protected indol-2-ylmethyl derivatives with moderately active nitroarenes (step-by-step procedure). Synthesis of 10-*n*-butyl-2-chloro-11-(phenylsulfonyl)-10*H*-indolo[3,2-*b*]quinoline (3). Typical procedure.** Triethylamine (0.61 g, 6 mmol) was added dropwise to a solution of 1-butylindol-2-ylmethyl phenyl sulfone (**1a**, 0.35 g, 1 mmol) and 4-chloronitrobenzene (0.24 g, 1.5 mmol) in anhydrous THF (12 mL) and cooled to −70 °C. Then 0.85 mL of a 1.4 M solution of *t*-BuOK (1.2 equiv) in THF was added dropwise and the temperature was maintained below −65 °C. After 20 min of stirring of the mixture, TMSCl (0.65 g, 6 mmol) was added dropwise at this temperature. The solution was stirred for 3 h at −65 to −60 °C, then allowed to reach rt, and stirred overnight (18–21 h). The reaction mixture was quenched with H_2_O (5 mL) and saturated aqueous NH_4_Cl solution (25 mL). The mixture was extracted with EtOAc (5 × 25 mL), and the combined extract was washed with brine (50 mL), dried over Na_2_SO_4_ and evaporated. The crude product was purified by column chromatography (silica gel, toluene/hexane 1:1, then toluene, then EtOAc/toluene 1:5). Yellow solid, yield 266 mg (59%); mp 176–178 °C (EtOAc/hexane); IR (KBr): 3052, 2958, 2900, 2874, 2855, 1971, 1936, 1903, 1820, 1780, 1736, 1618, 1578, 1547, 1496, 1480, 1460, 1444, 1424, 1408, 1382, 1366, 1332, 1320, 1302, 1221 cm^−1^; ^1^H NMR (500 MHz, CDCl_3_) δ 0.78 (t, *J* = 7.4 Hz, 3H), 1.06 (sex, *J* = 7.5 Hz, 2H), 1.63 (quin, *J* = 7.7 Hz, 2H), 4.69 (t, *J* = 7.8 Hz, 2H), 7.40–7.46 (m, 3H), 7.50–7.56 (m, 3H), 7.71 (td, *J* = 7.7, 0.9 Hz, 1H), 7.78 (d, *J* = 7.6 Hz, 2H), 8.19 (d, *J* = 8.9 Hz, 1H), 8.47 (d, *J* = 7.6 Hz, 1H), 8.64 (d, *J* = 2.1 Hz, 1H); ^13^C NMR (125 MHz, CDCl_3_) δ 13.62, 19.95, 30.20, 49.60, 111.55, 119.20, 122.04, 122.07, 122.21, 123,17, 123.55, 126.09, 127.46, 129.17, 131.06, 131.26, 133.03, 133.13, 133.30, 141.92, 143.59, 147.24, 150.26; EIMS *m*/*z* (%): 450 (31), 229 (22), 448 (73), 405 (18), 357 (12), 345 (13), 343 (12), 342 (13), 341 (37), 340 (12), 339 (12), 309 (15), 308 (21), 307 (65), 306 (62), 305 (74), 293 (17), 292 (10), 291 (10), 278 (10), 267 (42), 266 (23), 265 (100), 251 (13), 229 (13), 224 (11), 216 (11), 215 (19), 101 (30), 91 (10), 85 (17), 83 (16), 77 (19), 72 (15), 71 (22), 69 (11), 57 (39), 56 (11), 55 (44), 43.5 (34), 41.5 (28), 39.5 (11); HRMS (EI) *m*/*z*: calcd for C_25_H_21_N_2_O_2_S^35^Cl*^+^, 448.1012; found, 448.1015.

**Reactions of *N*-Boc protected indol-2-ylmethyl derivatives with active nitroarenes (one-pot procedure). Typical procedure. Synthesis of 5,6-di(*tert*-butoxycarbonyl)indolo[2,3-*b*][1,7]naphthiridine (15).** 3-Nitropyridine (0.15 g, 1.2 mmol) and *tert*-butyl 1-(*tert*-butoxycarbonyl)indol-2-ylacetate (**1b**, 0.33 g, 1 mmol) were dissolved in 5 mL of MeCN. The resulted mixture was stirred at room temperature until dissolution, then were added TMSCl (0.65 g, 6 mmol) in one portion and DBU (0.91 g, 6 equiv) dropwise (during 1 min). The reaction vial was stoppered and the mixture stayed without stirring at room temperature. Progress of the reaction was examined by tlc. After completion of the reaction the mixture was poured onto saturated aqueous NH_4_Cl solution (30 mL), extracted with EtOAc (5 × 25 mL), the combined extract was washed with brine (50 mL), dried over Na_2_SO_4_ and evaporated. The crude product was purified by column chromatography (silica gel, EtOAc/toluene: 1:10, then 1:5, then 1:2). Yellow solid, yield 167 mg (40%); mp 168–170 °C (EtOAc/hexane; then washed with pentane); IR (KBr): 3032, 3005, 2974, 2933, 2909, 2877, 1739 (CO), 1725 (CO), 1620, 1592, 1572, 1484, 1461, 1387, 1369, 1343, 1311, 1294, 1242, 1208 cm^−1^; ^1^H NMR (500 MHz, CDCl_3_) δ 1.77 (s, 9H), 1.78 (s, 9H), 7.50 (t, *J* = 7.5 Hz, 1H), 7.67 (td, *J* = 7.8, 1.1 Hz, 1H), 8.11–8.15 (m, 2H), 8.47 (d, *J* = 7.6 Hz, 1H), 8.65 (d, *J* = 5.8 Hz, 1H), 9.66 (s, 1H); ^13^C NMR (125 MHz, CDCl_3_) δ 28.40, 28.56, 83.97, 85.66, 116.31, 117.53, 121.99, 123.55, 124.35, 124.38, 127.16, 128.99, 131.53, 140.73, 142.28, 143.37, 149.70, 150.50, 154.09, 163.93. HRMS (ESI) *m*/*z*: calcd for C_24_H_26_N_3_O_4_^+^, 420.1923.; found, 420.1917.

## Supporting Information

File 1 Experimental part and copies of NMR spectra.
