# Supplementary material for: Transition-metal-free [3 + 3] annulation of indol-2-ylmethyl carbanions to nitroarenes. A novel synthesis of indolo[3,2-b]quinolines (quindolines)
Source: Beilstein J Org Chem. 2018 Jan 23;14:194–202. doi: 10.3762/bjoc.14.14 (PMC5789443; doi:10.3762/bjoc.14.14)
Supplement: File 1 — Experimental part and copies of NMR spectra. [file Beilstein_J_Org_Chem-14-194-s001.pdf]

Supporting Information

for

Transition-metal-free [3 + 3] annulation of indol-2-ylmethyl carbanions to nitroarenes. A novel synthesis of indolo[3,2-*b*]quinolines (quindolines)

Michał Nowacki and Krzysztof Wojciechowski*

Address: Institute of Organic Chemistry, Polish Academy of Sciences, Kasprzaka 44/52, 01-224 Warsaw, Poland

Email: Krzysztof Wojciechowski - kwojciechowski@icho.edu.pl

*Corresponding author

Experimental part and copies of NMR spectra

1. Experimental S2

1.1. Synthesis of starting materials S2

1.2. Reactions of protected indol-2-ylmethyl derivatives with moderately active nitroarenes (*step-by-step* procedure). S15

1.3. Reactions of *N*-Boc protected indol-2-ylmethyl derivatives with active nitroarenes (*one-pot* procedure). S21

2. Copies of ^{1}H and ^{13}C NMR spectra S27

1. Experimental

Melting points are uncorrected. ^{1}H and ^{13}C NMR spectra were recorded on Bruker Avance 500 or Varian vnmr s500 (both 500 MHz for ^{1}H and 125 MHz for ^{13}C spectra) instruments at 298 K. Chemical shifts are expressed in parts per million (ppm) referred to TMS, coupling constants in hertz (Hz). Electron impact mass spectra (EI, 70 eV) were obtained on an AutoSpec Premier spectrometer. Electrospray mass spectra (ESI) were obtained on 4000 Q-TRAP and SYNAPT G2-S HDMS. Silica gel (Merck 60, 230–400 mesh) was used for column chromatography (CC). Toluene or hexane/ethyl acetate mixtures were used for elution. TLC analyses were performed on Merck silica gel 60 F_{254} aluminum plates with hexane/ethyl acetate mixtures. All reagents and solvents were of reagent grade or purified according to standard methods before use. All reactions were run under argon atmosphere.

1.1. Synthesis of starting materials

The sulfone **1a** and nitrile **1d** were obtained in a few simple steps from ethyl indole-2-carboxylate as shown in the Scheme S1. This ester was alkylated with *n*-butyl bromide in the presence of K_2CO_3 and tetrabutylammonium bromide. The *N*-alkylated ester was transformed into *N*-methylamide that was then reduced with $LiAlH_4$ to the corresponding amine. Quaternization of the amine with methyl iodide in the presence of Na_2CO_3 furnished ammonium salt in which the trimethylamine group was then replaced with the phenylsulfonyl group during heating with sodium benzenesulfinate in DMSO at 160 ^{o}C to give the desired sulfone **1a**.

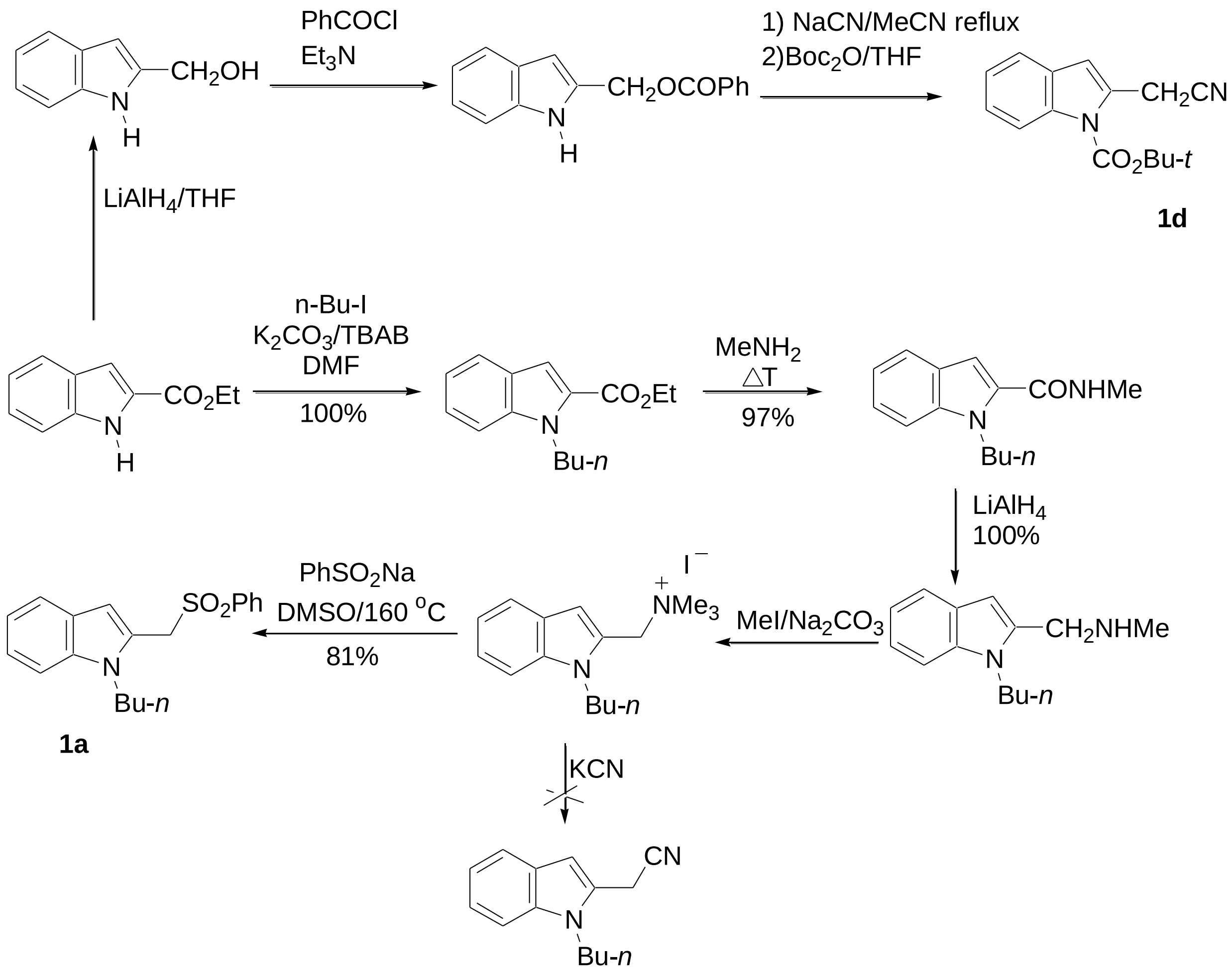

Scheme S1: Synthesis of indol-2-ylmethyl phenyl sulfone and nitrile.

Attempted replacement of the ammonium group with a cyanide anion to obtain the corresponding indol-2-ylacetonitrile was unsuccesful. At room temperature no replacement of the trimethylammonium group occurred. A prolonged heating resulted in the

consumption of the ammonium salt occurred but no expected acetonitrile was formed and the reactants turned into tars. It is in contrast to the reactions of trimethylammonium salts of gramine that are commonly used for synthesis of indol-3-ylmethyl derivatives. The indol-2-ylcacetonitrile was synthesized from ethyl indole-2-carboxylate via reduction to alcohol which was then esterified with benzoyl chloride, and in the formed benzoate replacement of benzoate with cyanide anion furnished the nitrile.

The desired *tert*-butyl indol-2-ylacetate was obtained in three steps as shown in the Scheme S2. Condensation of 2-nitrophenylacetic acid and Meldrum's acid followed by esterification with tert-butanol furnished the ketoester [Bradshaw, B.; Parra, C.; Bonjoch, J., *Organic Lett*. **2013**, *15*, 2458-2461], which upon reduction with zinc in the presence of ammonium chloride cyclized to *tert*-butyl indol-2-ylacetate.

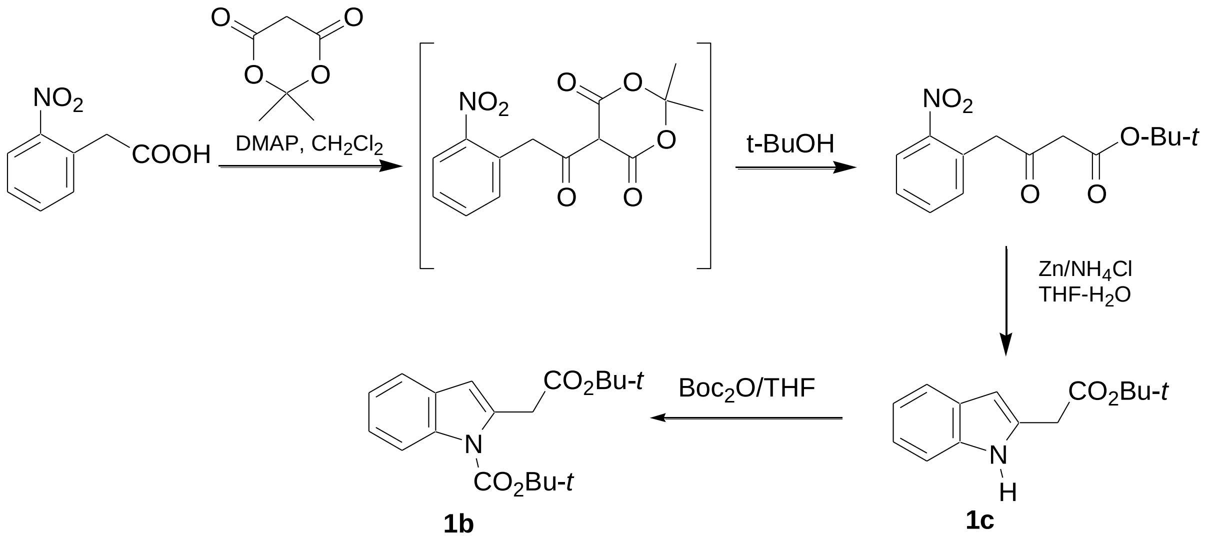

Scheme S2: Synthesis of tert-butyl indol-2-ylacetate.

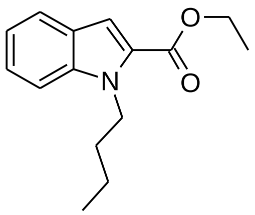

Ethyl 1-butylindole-2-carboxylate

To a solution of ethyl indole-2-carboxylate (7.51 g; 39.7 mmol) and Bu_4NBr (0.38 g; 1.2 mmol) dissolved in dry DMF (20 mL), solid K_2CO_3 (21.94 g; 158.7 mmol) was poured and BuI (6.8 mL; 10.95 g; 59.5 mmol) was added dropwise. The mixture was vigorously stirred for 42 h at room temperature and then heated for 1 h at 50 °C. Solid was filtered out, washed with Et_2O (2 × 50 mL). The organic phases were combined and washed with water (150 mL). The aqueous phase was washed with Et_2O (4 × 50 mL). The organic phases again were combined, washed with water (2 × 25 mL), brine (1 × 100 mL), dried over Na_2SO_4 and the solvent was evaporated in vacuo. The product was pure enough to use it in the next step without further purification.

Dark yellow oil, yield 10.00 g (~100%).

IR (CH_2Cl_2) 3059, 2958, 2932, 2872, 1712, 1614, 1518, 1480, 1465, 1413, 1368, 1354, 1320, 1297, 1248, 1235 cm^{-1}.

^{1}H NMR (Bruker 500 MHz, $CDCl_3$) δ = 0.94 (t, *J* = 7.4 Hz, 3H), 1.37 (sex, *J* = 7.6 Hz, 2H), 1.41 (t, *J* = 7.1 Hz, 3H), 1.78 (sex, *J* = 7.6 Hz, 2H), 4.37 (q, *J* = 7.1 Hz, 2H), 4.56 (t, *J* = 7.5 Hz, 2H), 7.13 (t, *J* = 7.3 Hz, 2H), 7.31 – 7.34 (m, 1H), 7.39 (d, *J* = 8.4 Hz, 1H), 7.67 (d, *J* = 8.0 Hz, 1H).

^{13}C NMR (Bruker 125 MHz, $CDCl_3$) δ = 13.84, 14.35, 20.20, 32.73, 44.53, 69.44, 110.37, 110.47, 120.38, 122.59, 124.74, 125.97, 127.52, 139.06, 162.02.

HRMS (ESI) m/z calcd for $C_{15}H_{20}NO_2^+$: 246.1494; found: 246.1487.

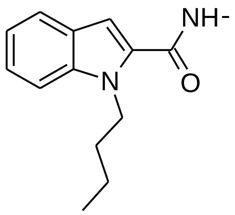

1-Butylindole-2-(*N*-methylcarboxamide)

Ethyl 1-butylindole-2-carboxylate (9.25 g; 37.70 mmol) dissolved in liquid $MeNH_2$ (14.33 g) was heated for 42 h at 80 °C in a sealed vessel. The pressure tube was cooled to room temperature, put in a cooling bath (acetone – solid carbodioxide), cautiously opened and $MeNH_2$ was evaporated. Crude product was dissolved in Et_2O and solid was precipitated from hexane.

White solid, yield 8.42 g (97%): mp 105-107 °C (Et_2O-hexane).

IR (KBr) 3298, 3078, 3058, 3032, 2952, 2927, 2870, 2802, 1765, 1641 (CO), 1614, 1552, 1456, 1410, 1362, 1315, 1279, 1213 cm^{-1}.

^{1}H NMR (Bruker 500 MHz, $CDCl_3$) δ =0.93 (t, *J* = 7.4 Hz, 3H), 1.35 (sex, *J* = 7.5 Hz, 2H), 1.79 (quin, *J* = 7.6 Hz, 2H), 3.00 (d, *J* = 4.9 Hz, 3H), 4.55 (t, *J* = 7.5 Hz, 2H), 6.21 (s, 1H), 6.78 (s, 1H), 7.13 (td, *J* = 7.3, 0,5 Hz, 1H), 7.29, (td, *J* = 7.6, 0.9 Hz, 1H), 7.39 (d, *J* = 8.3 Hz, 1H), 7.61 (d, *J* = 8.0 Hz, 1H).

^{13}C NMR (Bruker 125 MHz, $CDCl_3$) δ = 13.82, 20.19, 26.36, 32.71, 44.42, 103.57, 110.42, 120.29, 121.75, 123.76, 126.15, 131.90, 138.22, 163.24.

HRMS (ESI) m/z calcd for $C_{14}H_{18}N_2NaO^+$: 253.1317; found: 253.1307.

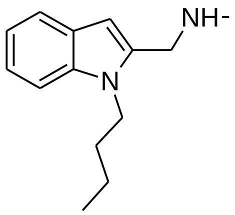

N-Methyl-N-(1-butylindol-2-ylmethyl)amine

The solution of 1-butylindole-2-(*N*-methylcarboxamide) (7.33 g; 31.8 mmol) dissolved in dry THF (60 mL) was added dropwise to a stirred suspension of $LiAlH_4$ (7.24 g; 227.7 mmol) in dry THF (40 mL). The mixture was refluxed for 24 h, cooled to room temperature and cautiously quenched with water (7.1 mL), 15% NaOH (7.1 mL) and again with water (21.8 mL). The solution was stirred for 1 h and the white precipitate was filtered out. The filter cake was washed several times with Et_2O (overall 220 mL). Combined organic phases were dried over K_2CO_3 and the solvents were evaporated in vacuo. Product was pure enough to use it in the next step without further purification.

Yellow oil, yield 6.99 g; (~100%).

IR (KBr) 3327, 3054, 3031, 2957, 2930, 2871, 2789, 1911, 1874, 1758, 1610, 1576, 1549, 1462, 1410, 1362, 1335, 1314, 1259, 1238, 1210 cm^{-1}.

^{1}H NMR (Bruker 500 MHz, $CDCl_3$) δ = 0.95 (t, *J* = 7.4 Hz, 3H), 1.38 (sex, *J* = 7.5 Hz, 2H), 1.75 (quin, *J* = 7.6 Hz, 2H), 1.88 (s, 1H), 2,50 (s, 3H), 3.89 (s, 2H), 4.17 (t, *J* = 7.6 Hz, 2H), 6.38 (s, 1H), 7.06 (t, *J* = 7.4 Hz, 1H), 7.16 (t, *J* = 7.6 Hz, 1H), 7.30 (d, *J* = 8.2 Hz, 1H), 7.55 (d, *J* = 7.9 Hz, 1H).

^{13}C NMR (Bruker 125 MHz, $CDCl_3$) δ = 13.82, 20.35, 32.39, 36.05, 43.25, 47.97, 100.83, 109.34, 119.17, 120.28, 121.04, 127.65, 137.07, 137.79.

MS (EI): m/z (%) = 217 (19), 216 (100), 215 (18), 203 (13), 187 (23), 186 (59), 185 (95), 184 (13), 172 (34), 171 (15), 170 (63), 160 (17), 157 (14), 156 (21), 145 (38), 144 (88), 143 (19), 132 (14), 131 (34), 130 (91), 129 (15), 128 (12), 118 (20), 117 (16), 115 (17), 103 (14), 102 (10), 89 (12), 77 (14), 44.5 (20), 42.5 (18), 41.5 (14).

HRMS (EI) m/z calcd for $C_{14}H_{20}N_2$: 216.1626; found: 216.1630.

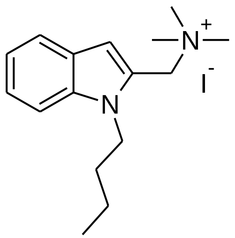

(1-*n*-Butylindol-2-ylmethyl)trimethylammonium iodide

Methyl-(1-*n*-butylindol-2-yl)amine (6.94 g; 32.1 mmol) dissolved in anhydrous MeCN (40 mL) was added to a suspension of Na_2CO_3 (13.61 g; 128.4 mmol in dry MeCN (50 mL). After putting reaction flask in a cooling bath (acetone – solid carbodioxide) MeI (18.23 g; 8.0 mL) was added dropwise. After removing cooling bath, the mixture was stirred for 22 h. The solid was filtered out and washed several times with MeCN. Colmbined organic phases were evaporated in vacuo giving 5.59 g of crude product.

The solid, which was filtered out after the reaction was suspended into in water (50 mL) and stirred for 0.5 h to separate inorganic salts from the product which turned out to be insoluble in water. The solid was filtered out, washed with water (20 mL) and dried what allowed to obtain additional 10.05 g of product which was successfully used directly in the next. A sample for analytical purposes was crystallized from EtOH-pirydyne 2:1 and washed with cooled EtOH.

White solid, overall yield 15.65 g (> 100%) due to presence of occluded inorganic impurities: mp 201-204 °C (EtOH-pirydyne).

IR (KBr) 3453, 3092, 3047, 2998, 2954, 2869, 2810, 2549, 2517, 2488, 1929, 1901, 1789, 1726, 1611, 1560, 1534, 1482, 1460, 1409, 1379, 1348, 1327, 1348, 1327, 1313, 1271, 1241, 1203 cm^{-1}.

^{1}H NMR (Varian 500 MHz, DMSO) δ = 0.85 (t, *J* = 7.4 Hz, 3H), 1.24 (sex, *J* = 7.6 Hz, 2H), 1.58 (sex, *J* = 7.5 Hz, 2H), 3.11 (s, 9H), 4.35 (t, *J* = 7.3 Hz, 2H), 4.81 (s, 2H), 6.85 (s, 1H), 7.11 (t, *J* = 7.4 Hz, 1H), 7.26 (td, *J* = 7.7, 0.8 Hz, 1H), 7.57 (d, *J* = 8.3 Hz, 1H), 7.65 (d, *J* = 7.9 Hz, 1H).

^{13}C NMR (Varian 125 MHz, DMSO) δ = 13.69, 19.32, 31.81, 42.87, 51.64, 58.91, 108.50, 111.13, 119.97, 121.05, 122.86, 126.68, 126.72, 137.14.

HRMS (ESI) m/z calcd for $C_{16}H_{25}N_2^+$: 245.2018; found: 245.2018.

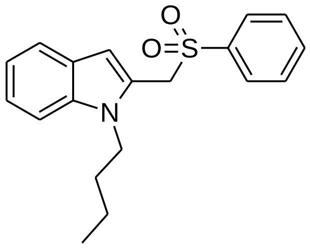

1-Butylindol-2-ylmethyl phenyl sulfone (**1a**)

(1-Butylindol-2-ylmethyl)trimethylammonium iodide (5.00 g; ~13.43 mmol) and benzenesulfinic acid sodium salt (4.41 g; 26.9 mmol) were dissolved in DMSO (30 mL) after heating the suspension to 90 °C. Then the mixture was stirred at 155–160 °C for 2 h. After cooling to room temperature, the mixture was poured into water (125 mL) and extracted with CH_2Cl_2 (5 × 30 mL). Cobbined organic phases were dried with Na_2SO_4 and solvent was evapoated in vacuo. Crude product was crystallized from EtOH.

White solid, yield 81% 3.57 g; (81%) mp 144-145.5 °C (EtOH).

IR (KBr) 3087, 3056, 3036, 2981, 2952, 2929, 2870, 1990, 1963, 1897, 1805, 1763, 1611, 1584, 1538, 1481, 1457, 14447, 1416, 1368, 1349, 1308, 1252, 1224 cm^{-1}. ^{1}H NMR (Bruker 500 MHz, DMSO) δ = 0.86 (t, *J* = 7.4 Hz, 3H), 1.26 (sex, *J* = 7.5 Hz, 2H), 1.56 (quin, *J* = 7.6 Hz, 2H), 4.09 (t, *J* = 7.6 Hz, 2H), 4.98 (s, 2H), 6.23 (s, 1H), 7.00 (t, *J* = 7.4 Hz, 1H), 7.14 (td, *J* = 7.5, 0.9 Hz, 1H), 7.41 (d, *J* = 8.2 Hz, 1H), 7.45 (t, *J* = 7.8 Hz, 1H), 7.60 (d, *J* = 7.8 Hz, 1H), 7.74 (t, *J* = 7.4 Hz, 1H), 7.78 – 7.81 (m, 2H).

^{13}C NMR (Bruker 125 MHz, DMSO) δ = 13.59, 19.44, 31.59, 42.05, 53.14, 104.89, 110.14, 119.28, 120.19, 121.61, 126.62, 126.75, 128.03, 129.12, 133.92, 136,68, 138.47.

HRMS (ESI) m/z calcd for $C_{19}H_{21}NO_2NaS^+$: 350.1191; found: 350.1181.

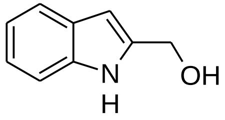

Indol-2-ylmethylcarbinol

The solution of ethyl indole-2-carboxylate (4.73 g; 25 mmol) dissolved in dry THF (25 mL) was added dropwise to a stirred suspension of $LiAlH_4$ (1.23 g; 32.3 mmol) in dry THF (40 mL). The mixture was stirred 2.5 h at room temperature and cautiously

quenched by adding dropwise: water (1.2 mL), 15% NaOH (1.2 mL) and again water (3.7 mL). The solution was stirred for 1 h and the white precipitate was filtered out. The filter cake was washed with Et_2O (4 × 25 mL). The combined organic phases were dried over Na_2SO_4 and the solvents were evaporated in vacuo. The crude product was crystallized from EtOAc-hexane 1:2 and washed with EtOAc-hexane 1:20. The product that remained in the filtrate was separated by column chromatography (silica gel, EtOAc-hexane 1:4, then 1:2).

White solid, overall yield 3.39 g (92%); mp 74-75.5 °C (EtOAc-hexane).

^{1}H NMR (Bruker 500 MHz, $CDCl_3$) δ 2.16 (s 1H), 4.73 (s, 2H), 6.37 (s, 1H), 7.10 (t, *J* = 7.4 Hz; 1H), 7.17 (t, *J* = 7.6 Hz; 1H), 7.27 (d, *J* = 8.1 Hz; 1H), 7.57 (t, *J* = 7.7 Hz; 1H), 8.32 (s, 1H).

^{13}C NMR (Bruker 125 MHz, $CDCl_3$) δ = 58.62, 100.54, 110.96, 119.92, 120.60, 122.17, 128.05, 136.36, 137.50.

MS (EI): m/z (%) = 148 (15), 147 (98), 146 (25), 131 (26), 130 (98), 129 (100), 128 (25), 119 (11), 118 (35), 117 (23), 103 (15), 102 (20), 91 (24), 90 (16), 89 (26), 84 (24), 77 (19), 73 (10), 69 (10), 66 (28), 65 (11), 63 (16), 57 (15), 51 (12), 43 (26), 39 (11).

HRMS (EI) m/z calcd for $C_9H_9NO^{*+}$: 147.0684; found: 147.0678.

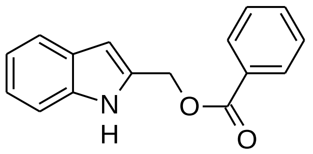

Indol-2-ylmethyl benzoate

To a flask, put in a cooling (ice – water), containing a solution of indol-2-ylmethylcarbinol (3.02 g; 20.5 mmol) in dry THF (103 mL), NEt_3 (2.49 g; 3.4 mL; 24.6 mmol) and PhCOCl (3.46 g; 2.9 mL; 24.6 mmol) were added dropwise. The cooling bath was removed and the mixture was strirred for 2 h 45 min at room temperature. Saturated aquous solution of $NaHCO_3$ (75 mL) was added and the mixture was stirred for 0.5 h. The mixture was extracted with EtOAc (5 × 25 mL). The extract was washed with brine (1 × 100 mL), dried over Na_2CO_3 and solvents were evaporated in vacuo. The crude product was dissolved in mixture CH_2Cl_2-hexane and was precipitated during concentration (using rotary evaporator), collected and washed with hexane.

White solid, yield 3.66 g (71%): mp 126.5-128 °C (EtOAc-hexane; then washed with pentane).

IR (KBr) 3348, 3079, 3055, 3032, 3017, 2963, 1932, 1900, 1784, 1701 (CO), 1616, 1600, 1583, 1550, 1429, 1453, 1423, 1383, 1344, 1318, 1277, 1218 cm^{-1}.

^{1}H NMR (Bruker 500 MHz, $CDCl_3$) δ = 5.47 (s, 2H), 6.61 (s, 1H), 7.09 (t, *J* = 7.4 Hz; 1H), 7.19 (t, *J* = 7.5 Hz; 1H), 7.35 (d, *J* = 8.1 Hz; 1H), 7.42 (t, *J* = 7.7 Hz; 2H), 7.55 (t, *J* = 7.4 Hz; 1H), 7.60 (d, *J* = 7.9 Hz; 1H), 8.05 (d, *J* = 7.4 Hz; 2H), 8.71 (s, 1H).

^{13}C NMR (Bruker 125 MHz, $CDCl_3$) δ = 60.24, 104.09, 111.14, 119.99, 120.89, 122.79, 127.56, 128.43, 129.68, 129.78, 133.04, 133.34, 136.63, 167.75.

HRMS (ESI) m/z calcd for $C_{16}H_{13}NNaO_2^+$: 274.0844.; found: 274.0833.

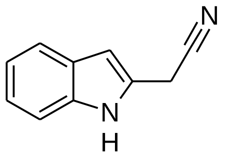

Indol-2-ylacetonitrile

To a sollution of indol-2-ylmethyl benzoate (3.54 g; 14.1 mmol) in MeCN (100 mL) was poured NaCN (1.84 g, 28.2 mmol). The suspension was vigoriously stirred and refluxed (~80 °C) for 17 h. After cooling to room temperature, saturated aquous solution of $NaHCO_3$ (100 mL) was added. The mixture was extracted with EtOAc (5 × 25 mL). The extract was washed with brine (1 × 100 mL), dried over Na_2SO_4 and solvents were evaporated in vacuo. The product was separated by column chromatography (silica gel, EtOAc-hexane, 1:10, then 1:4).

Brown solid, yield 1.35 g (61%): mp 97-98.5 °C (CH_2Cl_2-hexane; then washed with pentane).

IR (KBr) 3346 (NH), 3058, 3022, 2950, 2254 (CN), 1620, 1594, 1553, 1429, 1454, 1433, 1401, 1369, 1344, 1299, 1223 cm^{-1}.

^{1}H NMR (Bruker 500 MHz, $CDCl_3$) δ = 3.87 (s 2H), 6.46 (s, 1H), 7.08 – 7.16 (m, 1H), 7.16 – 7.25 (m, 1H), 7.30 – 7.36 (m, 1H), 7.52 – 7.60 (m, 1H), 8.16 (s, 1H). ^{13}C NMR (Bruker 125 MHz, $CDCl_3$) δ = 17.55, 102.76, 110.93, 116.31, 120.49, 120.54, 122.72, 125.74, 128.08, 136.51.

MS (EI): m/z (%) = 157 (22), 156 (100), 155 (88), 130 (50), 129 (44), 128 (25), 103 (11), 102 (21), 101 (20), 89 (12), 78 (21), 77 (20), 75 (11), 63 (16), 51 (17), 50 (10), 39 (10).

HRMS (EI) m/z calcd for $C_{10}H_8N_2^{*+}$: 156.0687; found: 156.0689.

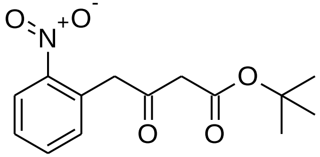

***Tert*-butyl 4-(2-nitrophenyl)-3-oxobutanoate**

To a flask, put in a cooling bath (ice – water), containing a sollution of Meldrum's acid (3.79 g; 26.3 mmol), 2-nitrophenylacetic acid (4.77 g; 26.3 mmol), and DMAP (3.53 g; 28.9 mmol) in CH_2Cl_2 (40 mL), DCC (6.51; 31.6 mmol) dissolved in CH_2Cl_2 (25 mL) was added dropwise. After 3 h the cooling bath was removed and the mixture was stirred for 20 h at room temperature. 1,3-Dicyclohexylurea was filtered out, washed with CH_2Cl_2 (50 mL). Combined filtrates were washed with saturated aquous solution of NH_4Cl (100 mL). Aquous phase was extracted with CH_2Cl_2 (2 × 25 mL). Combined extracts were washed with brine (100 mL), dried over Na_2SO_4 and the solvents were evaporated in vacuo. The product was separated by column chromatography (silica gel, hexane, then EtOAc-hexane, 1:4).

White solid, 5.68 g (77%): (CH_2Cl_2-hexane, then washed with pentane).

IR (KBr) 3089, 3073, 3037, 2981, 2936, 2876, 2855, 1731 (CO), 1705 (CO), 1613, 1577, 1526, 1479, 1455, 1431, 1409, 1393, 1370, 1341, 1315, 1301, 1283, 1254, 1206 cm^{-1}.

^{1}H NMR (Bruker 500 MHz, $CDCl_3$) δ = 1.50 (s, 9H), 2.09 (s, 2H), 4.25 (s, 2H), 7.31 (d, *J* = 7.6 Hz, 1H), 7.47 (td, *J* = 7.8, 1.3 Hz; 1H), 7.60 (td, *J* = 7.5, 1.2 Hz; 1H), 8.13 (dd, *J* = 8.2, 1.0 Hz;, 1H).

^{13}C NMR (Bruker 125 MHz, $CDCl_3$) δ = 27.98, 47.70, 50.59, 82.28, 123.57, 125.28, 128.59, 129.77, 133.63, 133.66, 166.20, 198.54.

HRMS (ESI) m/z calcd for $C_{14}H_{17}NNaO_5^+$: 302.1004; found: 302.1004.

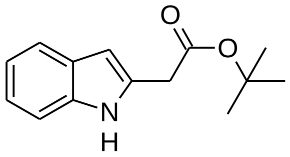

Tert-butyl indol-2-ylacetate (1c)

To a biphasic mixture of solution of *tert*-butyl 4-(2-nitrophenyl)-3-oxobutanoate (5.68 g; 20.3 mmol) in THF (125 mL) and saturated aquous solution of NH_4Cl (125 mL) was added zink dust (26.6 g; 406.6 mmol) in one portion. The mixture was vigoriously stirred for 3 h. Unreacted zinc was separated and washed with EtOAc (50 mL). The combined liquid phases were separated. The aquous phase was extracted with EtOAc (3 × 50 mL). The combined organic extracts were washed with brine (100 mL), dried over Na_2SO_4 and the solvents were evaporated in vacuo. The product was separated by column chromatography (silica gel, toluene-hexane, 1:1).

Brown solid, yield 3.21 g (68%): 112-114 mp °C (EtOAc-hexane; then washed with pentane).

IR (KBr) 3351, 3109, 3085, 3060, 3003, 2983, 2929, 2899, 2739, 2678, 2497, 1914, 1881, 1715 (CO), 1618, 1584, 1553, 1476, 1456, 1430, 1391, 1368, 1345, 1328, 1290, 1251, 1225 cm^{-1}.

^1H NMR (Bruker 500 MHz, $CDCl_3$) δ = 1.48 (s, 9H), 3.73 (s, 2H), 6.32 (d, *J* = 0.8 Hz, 1H), 7.07 (td, *J* = 7.1, 0.8 Hz; 1H), 7.14 (td, *J* = 7.6, 1.0 Hz; 1H), 7.33 (d, *J* = 7.8 Hz, 1H), 7.54 (d, *J* = 7.8 Hz, 1H), 8.71 (s, 1H).

^{13}C NMR (Bruker 125 MHz, $CDCl_3$) δ = 28.05, 35.00, 81.87, 101.51, 110.75, 119.69, 120.02, 121.52, 128.24, 131.26, 136.29, 169.93.

HRMS (ESI) m/z calcd for $C_{14}H_{17}NNaO_2^+$: 254.1157; found: 254.1150.

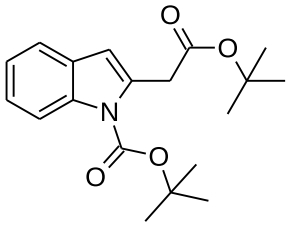

tert-Butyl 1-(_tert_-butoxycarbonyl)indol-2-ylacetate (1b)

To a sollution of *tert*-butyl 2-indoleacetate (2.31 g, 10.0 mmol) in dry THF (40 mL), di-*tert*-butyl dicarbonate (2.51 g; 11.5 mmol) dissolved in THF (10 mL) was added dropwise. Afer pouring DMAP (61 mg; 0.5 mmol), the mixture was stirred for 24 h, and then concentrated. The crude product was dissolved in Et_2O (50 mL) and washed with saturated aquous solution of NH_4Cl (50 mL). Aquous phase was extracted with Et_2O (4 × 25 mL). The combined organic extracts were washed with brine (50 mL), dried over Na_2SO_4 and solvent was evaporated in vacuo. The product was separated by column chromatography (silica gel, hexane, then EtOAc 20:1).

Yellowish oil, yield 3.35 g (100%).

IR (CH_2Cl_2) 3054, 2982, 2927, 2853, 2305, 1733 (CO), 1597, 1572, 1475, 1454, 1422, 1381, 1370, 1329, 1265, 1222 cm^{-1}.

^1H NMR (Bruker 500 MHz, $CDCl_3$) δ = 1.71 (s, 9H), 4.15 (s, 2H), 6.73 (s, 1H), 7.24 (t, *J* = 7.4 Hz; 1H), 7.32 (t, *J* = 7.6 Hz; 1H), 7.52 (t, *J* = 7.7 Hz; 1H), 8.09 (t, *J* = 8.4 Hz; 1H).

^{13}C NMR (Bruker 125 MHz, $CDCl_3$) δ = 28.07, 28.20, 37.37, 80.91, 83.94, 110.04, 115.66, 120.14, 122.56, 123.69, 128.95, 134.26, 136.42, 150.51, 169.64.

HRMS (ESI) m/z calcd for $C_{19}H_{25}NNaO_4^+$: 354.1681; found: 354.1676.

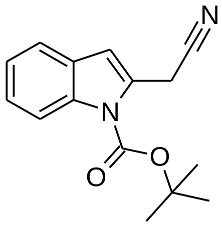

1-(*tert*-Butoxycarbonyl)indol-2-ylacetonitrile (1d).

To a solution of indol-2-ylacetonitrile (1.31 g, 8.4 mmol) in dry THF (30 mL), di-*tert*-butyl dicarbonate (2.20 g; 10.1 mmol) dissolved in THF (10 mL) was added dropwise. Afer pouring DMAP (51 mg; 0.4 mmol), the mixture was stirred for 24 h, and then concentrated. The crude product was dissolved in Et_2O (25 mL) and washed with saturated aquous solution of NH_4Cl (50 mL). The aquous phase was extracted with Et_2O (4 × 25 mL). The combined organic extracts were washed with brine (100 mL), dried over Na_2SO_4 and solvent was evaporated in vacuo. The product was separated by column chromatography (silica gel, toluene-hexane, 1:1, then 2:1). Pale brown solid, yield 1.18 g (55%): (CH_2Cl_2-hexane).

IR (KBr) 3464, 3123, 3088, 3072, 3034, 3008, 2983, 2975, 2952, 2934, 2925, 2910 2887, 2870, 2248 (CN), 1743 (CO), 1598, 1568, 1473, 1453, 1425, 1390, 1371, 1346, 1329, 1304, 1282, 1256, 1240, 1216 cm^{-1}.

^{1}H NMR (Bruker 500 MHz, $CDCl_3$) δ = 1.71 (s, 9H), 4.15 (s, 2H), 6.73 (s, 1H), 7.24 (t, *J* = 7.4 Hz; 1H), 7.32 (t, *J* = 7.6 Hz; 1H), 7.52 (t, *J* = 7.7 Hz; 1H), 8.09 (t, *J* = 8.4 Hz; 1H).

^{13}C NMR (Bruker 125 MHz, $CDCl_3$) δ = 20.20, 28.15, 85.27, 110.33, 115.73, 116.77, 120.61, 123.25, 124.77, 128.27, 129.04, 136.62, 150.10.

HRMS (ESI) m/z calcd for $C_{15}H_{16}N_2NaO_2^+$: 279.1109.; found: 279.1101.

1.2. Reactions of protected indol-2-ylmethyl derivatives with moderately active nitroarenes (*step-by-step* procedure).

General procedure.

Triethylamine (6 mmol) was added dropwise to a solution of sulfone **1a** or ester **1b,c** (1 mmol) and nitroarene (1.5 - 2 mmol) in anhydrous THF (12 mL) cooled to −70 °C. Then 0.85 mL of a 1.4 M solution of *t*-BuOK (1.2 equiv, 2.2 equiv in the case of reaction

with **1c**) in THF was added dropwise and the temperature was maintained below −65 °C. After 20 min of stirring of the mixture, TMSCl (6 mmol, 7 mmol in the case of reaction with **1c**) was added dropwise at this temperature. The solution was stirred for 3 h at −65 to −60 °C, then allowed to reach rt, and stirred overnight (18–21 h). The reaction mixture was quenched with H_2O (5 mL) and saturated aqueous NH_4Cl (25 mL). The mixture was extracted with EtOAc (5 × 25 mL), and the extract was washed with brine (50 mL), dried over Na_2SO_4 and evaporated. The crude product was purified by column chromatography using appropriate solvent (or mixture of solvents).

The following compounds were obtained:

10-*n*-Butyl-2-chloro-11-(phenylsulfonyl)-10*H*-indolo[3,2-*b*]quinoline (3)

Column chromatography (silica gel, toluene-hexane: 1:1, then toluene, then EtOAc-toluene 1:5).

Yellow solid, yield 266 mg (59%): mp 176-178 °C (EtOAc-hexane).

IR (KBr) 3052, 2958, 2900, 2874, 2855, 1971, 1936, 1903, 1820, 1780, 1736, 1618, 1578, 1547, 1496, 1480, 1460, 1444, 1424, 1408, 1382, 1366, 1332, 1320, 1302, 1221 cm^{-1}.

^{1}H NMR (500 MHz, $CDCl_3$) δ = 0.78 (t, *J* = 7.4 Hz, 3H), 1.06 (sex, *J* = 7.5 Hz, 2H), 1.63 (quin, *J* = 7.7 Hz, 2H), 4.69 (t, *J* = 7.8 Hz, 2H), 7.40 – 7.46 (m, 3H), 7.50 – 7.56 (m, 3H), 7.71 (td, *J* = 7.7, 0.9 Hz, 1H), 7.78 (d, *J* = 7.6 Hz, 2H), 8.19 (d, *J* = 8.9 Hz, 1H), 8.47 (d, *J* = 7.6 Hz, 1H), 8.64 (d, *J* = 2.1 Hz, 1H).

^{13}C NMR (125 MHz, $CDCl_3$) δ = 13.62, 19.95, 30.20, 49.60, 111.55, 119.20, 122.04, 122.07, 122.21, 123,17, 123.55, 126.09, 127.46, 129.17, 131.06, 131.26, 133.03, 133.13, 133.30, 141.92, 143.59, 147.24, 150.26.

MS (EI): m/z (%) = 450 (31), 229 (22), 448 (73), 405 (18), 357 (12), 345 (13), 343 (12), 342 (13), 341 (37), 340 (12), 339 (12), 309 (15), 308 (21), 307 (65), 306 (62), 305 (74), 293 (17), 292 (10), 291 (10), 278 (10), 267 (42), 266 (23), 265 (100), 251 (13), 229 (13), 224 (11), 216 (11), 215 (19), 101 (30), 91 (10), 85 (17), 83 (16), 77 (19), 72 (15), 71 (22), 69 (11), 57 (39), 56 (11), 55 (44), 43,5 (34), 41.5 (28), 39.5 (11).

HRMS (EI) m/z calcd for $C_{25}H_{21}N_2O_2S^{35}Cl^{*+}$: 448.1012; found: 448.1015.

2-Bromo-10-*n*-butyl-11-(phenylsulfonyl)-10*H*-indolo[3,2-*b*]quinoline (4)

Column chromatography (silica gel, toluene).

Yellow solid, yield 289 mg (59%): mp 179-181 °C (EtOAc-hexane).

IR (KBr); 3121, 3091, 3055, 3023, 2999 2958, 2933, 2900, 2873, 1898, 1825, 1738, 1616, 1598, 1575, 1542, 1477, 1460, 1447, 1419, 1407, 1365, 1332, 1315, 1300, 1226 cm^{-1}.

^{1}H NMR (500 MHz, $CDCl_3$) δ = 0.78 (t, *J* = 7.4 Hz, 3H), 1.06 (sex, *J* = 7.4 Hz, 2H), 1.64 (quin, *J* = 7.7 Hz, 2H), 4.69 (t, *J* = 7.8 Hz, 2H), 7.41 – 7.47 (m, 3H), 7.51 – 7.56 (m, 2H), 7.65 (dd, *J* = 9.0, 2.1 Hz, 1H), 7.71 (t, *J* = 7.8, Hz, 1H), 7.77 (d, *J* = 7.6 Hz, 2H), 8.11 (d, *J* = 8.9 Hz, 1H), 8.47 (d, *J* = 7.6 Hz, 1H), 8.79 (d, *J* = 2.0 Hz, 1H). ^{13}C NMR (125 MHz, $CDCl_3$) δ = 13.65, 19.97, 30.19, 49.64, 111.58, 119.16, 121.46, 122.04, 122.07, 122.25, 123.46, 126.17, 127.72, 129.16, 130.03, 131.11, 131.33, 132.90, 133.35, 142.06, 143.52, 147.30, 150.36.

HRMS (ESI): m/z calcd for $C_{25}H_{22}N_2O_2SBr^{*+}$: 493.0585; found: 493.0584.

10-*n*-Butyl-2-iodo-11-(phenylsulfonyl)-10*H*-indolo[3,2-*b*]quinoline (5)

Column chromatography (silica gel, toluene).

Yellow solid, yield 176 mg (33%): mp 171-172 °C (EtOAc-hexane).

IR (KBr) 3117, 3090, 3049, 2955, 2871, 1900, 1740, 1614, 1595, 1572, 1538, 1494, 1476, 1459, 1445, 1407, 1364, 1332, 1314, 1259, 1225 cm^{-1}.

^{1}H NMR (500 MHz, $CDCl_3$) δ = 0.80 (t, *J* = 7.4 Hz, 3H), 1.09 (sex, *J* = 7.5 Hz, 2H), 1.68 (quin, *J* = 7.7 Hz, 2H), 4.72 (t, *J* = 7.8 Hz, 2H), 7.40 – 7.46 (m, 3H), 7.51 – 7.55 (m, 2H), 7.72 (td, *J* = 7.8, 0.9 Hz, 1H), 7.74 – 7.77 (m, 2H), 7.80 (dd, *J* = 8.8, 1.7 Hz, 1H), 7.95 (d, *J* = 8.8 Hz, 1H), 8.47 (d, *J* = 7.6 Hz, 1H), 8.93 (d, *J* = 1.6 Hz, 1H).

^{13}C NMR (125 MHz, $CDCl_3$) δ = 13.64, 19.98, 30.14, 49.76, 93.24, 111.60, 119.15, 122.04, 122.05, 122.29, 123,60, 126.33, 129.08, 131.02, 131.33, 132.63, 133.18, 133.32, 135.27, 142.38, 143.56, 147.44, 150.46.

HRMS (ESI) m/z calcd for $C_{25}H_{22}IN_2O_2S^{+}$:541.0447; found: 541.0446.

10-*n*-Butyl-2-fluoro-11-(phenylsulfonyl)-10*H*-indolo[3,2-*b*]quinoline (6)

Column chromatography (silica gel, toluene).

Yellow solid, yield 65 mg (15%): mp 162-164 °C (MeOH-CH_2Cl_2).

IR (KBr); 3052, 2960, 2930, 2873, 1898, 1776, 1624, 1582, 1559, 1507, 1483, 1461, 1440, 1412, 1386, 1370, 1332, 1319, 1309, 1252, 1228 cm^{-1}.

^{1}H NMR (500 MHz, $CDCl_3$) δ = 0.77 (t, *J* = 7.4 Hz, 3H), 1.05 (heks, *J* = 7.5 Hz, 2H), 1.60 (quin, *J* = 7.7 Hz, 2H), 4.67 (t, *J* = 7.85 Hz, 2H), 7.35 – 7.47 (m, 4H), 7.50 – 7.56 (m, 2H), 7.70 (td, *J* = 7.7, 0.9 Hz, 1H), 7.79 (d, *J* = 7.5 Hz, 2H), 8.27 (dd, *J* = 9.15, 6.0 Hz, 1H), 8.35 (d, *J* = 11.9 Hz, 1H), 8.47 (d, *J* = 7.6 Hz, 1H).

^{13}C NMR (125 MHz, $CDCl_3$) δ = 13.61, 19.93, 30.25, 49.51, 108.92 (d, J_{CF} = 26Hz), 111.50, 116.75 (d, J_{CF} = 25Hz), 119.26, 122.03 (d, J_{CF} = 5Hz), 122.16, 123.79 (d, J_{CF} = 11Hz), 125.91, 129.21, 131.03, 132.07 (d, J_{CF} = 10Hz), 133.21, 133.24, 140.72, 143.60, 146.97, 149.56, 160.75; one C missing.

HRMS (ESI): m/z calcd for $C_{25}H_{22}N_2O_2SF^+$: 433.1386; found: 433.1384.

10-*n*-Butyl-11-(phenylsulfonyl)-2-(phenylthio)-10*H*-indolo[3,2-*b*]quinoline (7).

Column chromatography (silica gel, toluene).

Yellow solid, yield 34%: mp 149-151 °C (hexane-EtOAc).

IR (KBr) 3114, 3058, 2954, 2930, 2906, 2868, 1894, 1810, 1616, 1570, 1536, 1476, 1461, 1442, 1402, 1371, 1328, 1306, 1286, 1227 cm^{-1}.

^{1}H NMR (500 MHz, $CDCl_3$) δ = 0.80 (t, *J* = 7.3 Hz, 3H), 1.09 (sex, *J* = 7.4 Hz, 2H), 1.68 (quin, *J* = 7.6 Hz, 2H), 4.71 (t, *J* = 7.8 Hz, 2H), 7,32 (t, *J* = 8.0 Hz, 2H), 7.34 – 7.42 (m, 7 H), 7.46 (t, *J* = 7.5 Hz, 1H), 7.51 – 7.54 (m, 3H), 7.69 (td, *J* = 7.8, 1.0 Hz, 1H), 8.12 (d, *J* = 8.5 Hz, 1H), 8.39 (d, *J* = 2.0 Hz, 1H), 8.45 (d, *J* = 7.5 Hz, 1H).

^{13}C NMR (125 MHz, $CDCl_3$) δ = 13.65, 19.99, 30.16, 49.95, 111.58, 119.37, 121.86, 122.05, 122.29, 122.72, 123.39, 126.21, 127.72, 128.23, 128.91, 129.48, 130.24, 130.90, 132.92, 133.03, 133.05, 133.39, 136.75, 142.44, 143.63, 147.26, 149.64.

MS (EI): m/z (%) = 524 (25), 523 (51), 522 (100), 479 (11), 382 (23), 381 (34), 350 (11), 349 (36), 341(11), 340 (33), 339 (79), 337 (13), 325 (19), 324 (20), 308 (21), 307 (67), 306 (62), 305 (85), 294 (11), 293 (40), 292 (26), 272 (39), 255 (12), 230 (11), 229 (10), 215 (11), 169.5 (10), 169 (10), 77 (13).

HRMS (EI) m/z calcd for $C_{31}H_{26}N_2O_2S^+$: 522.1436; found: 522.1442.

Bis(*tert*-butyl) 2-chloro-10*H*-indolo[3,2-*b*]quinoline-10,11-dicarboxylate (8).

Column chromatography (silica gel, toluene-hexane: 2:1, then toluene).

Pale yellow solid, yield 125 mg (28%): mp 192-194 °C (EtOAc-hexane; then washed with pentane).

IR (KBr) 3137, 3078, 3060, 2979, 2932, 2874, 1735 (CO), 1719 (CO), 1619, 1589, 1573, 1496, 1484, 1458, 1429, 1384, 1370, 1344, 1305, 1273, 1258, 1244, 1215 cm^{-1}.

^{1}H NMR (500 MHz, $CDCl_3$) δ = 1.76 (s, 9H), 1.79 (s, 9H), 7.47 (t, *J* = 7.5 Hz, 1H), 7.63 (td, *J* = 7.8, 1.1 Hz, 1H), 7.67 (dd, *J* = 9.0, 2.2 Hz, 1H), 8.11 (d, *J* = 8.4 Hz, 1H), 8.20 (d, *J* = 9.0 Hz, 1H), 8.35 (d, *J* = 2.2 Hz, 1H), 8.42 (d, *J* = 7.6 Hz, 1H).

^{13}C NMR (125 MHz, $CDCl_3$) δ = 28.42, 28.56, 83.84, 85.23, 116.31, 121.64, 124.09, 124.17, 124.34, 124.40, 124.65, 127.19, 129.10, 130.92, 130.97, 132.50, 142.01, 144.28, 148.44, 150.68, 164.48.

HRMS (ESI) m/z calcd for $C_{24}H_{26}{}^{35}ClN_2O_4^+$: 453.1581.; found: 453.1581.

Bis(*tert*-butyl) 2-bromo-10*H*-indolo[3,2-*b*]quinoline-10,11-dicarboxylate (9).

Column chromatography (silica gel, EtOAc-hexane: 1:40, then 1:20).

Pale yellow solid yield 108 mg (22%): mp 191-193 °C (EtOAc-hexane; then washed with pentane).

IR (KBr) 3137, 3086, 3073, 3061, 3009, 2977, 2935, 1899, 1732 (CO), 1619, 1588, 1570, 1482, 1459, 1425, 1384, 1369, 1348, 1307, 1276, 1258, 1244, 1212 cm^{-1}.

^{1}H NMR (500 MHz, $CDCl_3$) δ = 1.76 (s, 9H), 1.79 (s, 9H), 7.47 (t, *J* = 7.4 Hz, 1H), 7.63 (t, *J* = 7.8, Hz, 1H), 7.80 (dd, *J* = 9.0, 1.7 Hz, 1H), 8.11 (d, *J* = 8.3 Hz, 1H), 8.14 (d, *J* = 8.9 Hz, 1H), 8.43 (d, *J* = 7.8 Hz, 1H), 8.53 (d, *J* = 2.0 Hz, 1H).

^{13}C NMR (125 MHz, $CDCl_3$) δ = 28.42, 28.55, 83.87, 85.26, 116.31, 120.75,121.70, 124.11, 124.29, 124.60, 124.88, 127.09, 127.52, 130.97, 131.03, 131.61, 142.05, 144.39, 148.50, 150.65, 164.44.

HRMS (ESI) m/z calcd for $C_{25}H_{26}{}^{79}BrN_2O_4{}^+$: 497.1076; found: 497.1066.

3-(1-*n*-Butylindol-2-yl)-5,7-dichloro-2,1-benzisoxazole (10).

Column chromatography (silica gel, toluene-hexane 1:1, then toluene).

Yellow solid, yield 87 mg (24%): mp 87.5-89 °C (MeOH).

IR (KBr) 3060, 2959, 2871, 1924, 1884, 1798, 1739, 1669, 1621, 1550, 1508, 1467, 1430, 1353, 1330, 1305, 1256, 1238, 1211 cm^{-1}.

^{1}H NMR (500 MHz, $CDCl_3$) δ = 0.92 (t, *J* = 7.4 Hz, 3H), 1.37 (sex, *J* = 7.5 Hz, 2H), 1.83 (quin, *J* = 7.6 Hz, 2H), 4.53 (t, *J* = 7.6 Hz, 2H), 7.14 (s, 1H), 7.20 (t, *J* = 7.4 Hz, 1H), 7.34 – 7.38 (m, 2H), 7.45 (d, *J* = 8.35 Hz), 7.71 (d, *J* = 8.0 Hz, 1H), 7.73 (s, 1H).

^{13}C NMR (125 MHz, $CDCl_3$) δ = 13.73, 20.14, 32.61, 45.39, 106.43, 110.32, 116.68, 118.14, 120.97, 121.93, 122.33, 124.71, 125.48, 127.52, 129.65, 131.59, 138.87, 154.47, 160.55.

MS (EI): m/z (%) = 362 (16), 361 (17), 360 (65), 359 (26), 358 (79), 343 (12), 341 (16), 332 (11), 331 (15), 329 (22), 319 (12), 318 (28), 317 (52), 316 (43), 315 (65), 306 (17), 305 (20), 304 (80), 303 (37), 302 (100), 301 (14), 280 (15), 239 (13), 203 (11), 41.5 (10).

HRMS (EI) m/z calcd for $C_{19}H_{16}N_2OCl_2{}^{*+}$: 358.0640; found: 358.0623.

1-(1-*n*-Butylindol-2-yl)naphth[1,2-*c*][1,2]oxazole (11).

Column chromatography (silica gel, toluene-hexane 1:1).

Beige solid, yield 87 mg (26%): mp 119-120 °C (EtOAc-hexane).

IR (KBr) 3053, 2951, 2861, 1913, 1880, 1758, 1679, 1624, 1577, 1532, 1469, 1440, 1423, 1405, 1383, 1344, 1323, 1267, 1243 cm^{-1}.

^{1}H NMR (500 MHz, $CDCl_3$) δ = 0.90 (t, *J* = 7.4 Hz, 3H), 1.37 (sex, *J* = 7.5 Hz, 2H), 1.85 (quin, *J* = 7.6 Hz, 2H), 4.54 (t, *J* = 7.5 Hz, 2H), 7.14 (s, 1H), 7.19 (t, *J* = 7.5 Hz, 1H), 7.32 – 7.36 (m, 2H), 7.46 (d, *J* = 8.3 Hz, 1H), 7.56 (d, *J* = 9.2 Hz, 1H), 7.60- 7.68 (m, 2H), 7.72 (d, *J* = 7.9 Hz, 1H), 7.76 (d, *J* = 7.4 Hz, 1H), 8.56 (dd, *J* = 7.8, 0.79 Hz, 1H).

^{13}C NMR (125 MHz, $CDCl_3$) δ = 13.75, 20.17, 32.60, 45.26, 105.50, 110.22, 114.16, 117.41, 120.55, 121.60, 122.04, 123.84, 124.12, 126.51, 127.36, 127.68, 127.89, 128.53, 129.82, 133.98, 138.44, 156,40, 158.02.

HRMS (ESI) m/z calcd for $C_{23}H_{21}N_2O^+$: 341.1654; found: 341.1652.

tert-Butyl 2-chloro-10_H_-indolo[3,2-_b_]quinoline-11-carboxylate (12).

Column chromatography (silica gel, CH_2Cl_2-hexane 1:4, than 1:2), then CH_2Cl_2, then CH_2Cl_2-EtOAc 1:10).

Yellow solid, yield 90 mg (26%): mp >305 °C (CH_2Cl_2-hexane, then washed with pentane).

IR (KBr) 3468 (NH), 3417, 3115, 3071, 2992, 2968, 2926, 1714 (CO), 1681, 1627, 1596, 1488, 1463, 1443, 1407, 1392, 1365, 1327, 1292, 1256, 1208 cm^{-1}.

^1H NMR (500 MHz, $CDCl_3$) δ = 1.83 (s, 9H), 7.36 (t, *J* = 7.4 Hz, 1H), 7.46 (t, *J* = 8.0 Hz, 1H), 7.58 – 7.65 (m, 2H), 8.28 (d, *J* = 8.9 Hz, 1H), 8.47 (d, *J* = 7.6 Hz, 1H), 9.14 (s, 1H), 9.88 (s, 1H).

^{13}C NMR (125 MHz, $CDCl_3$) δ = 28.53, 84.07, 111.26, 121.17, 121.42, 122.29, 123.57, 124.40, 126.78, 130.68, 131.49, 131.51, 133.17, 133.19, 134.39, 143.68, 166.64 (one signal missing).

HRMS (ESI) m/z calcd for $C_{20}H_{18}{}^{35}ClN_2O_2^+$: 353.1057; found: 353.1049.

tert-Butyl 2-chloro-10_H_-indolo[3,2-_b_]quinoline-11-carboxylate-5-oxide (13).

Column chromatography (silica gel, CH_2Cl_2, then CH_2Cl_2-EtOAc 1:10).

Brown solid, yield 44 mg (12%): mp 269-272 °C (CH_2Cl_2-hexane, then washed with pentane).

IR (KBr) 3464, 3410, 3134, 3098, 3005, 2972, 2928, 1708, 1681 (CO), 1622, 1599, 1579, 1490, 1458, 1435, 1413, 1392, 1366, 1341, 1318, 1285, 1266, 1239 cm^{-1}.

^1H NMR (500 MHz, $CDCl_3$) δ = 1.81 (s, 9H), 7.37 (t, *J* = 7.5 Hz, 1H), 7.43 (t, *J* = 8.0 Hz, 1H), 7.56 – 7.65 (m, 2H), 8.87 - 8.93 (m, 2H), 9.23 (s, 1H), 10.16 (s, 1H).

^{13}C NMR (125 MHz, $CDCl_3$) δ = 28.53, 84.12, 101.76, 110.62, 116.95, 120.73, 121.74, 123.56, 124.82, 125.28, 127.04, 127.06, 130.74, 135.26, 136.41, 138.14, 141.13, 165.71.

HRMS (ESI) m/z calcd for $C_{20}H_{18}{}^{35}ClN_2O_3^+$: 369.1006; found: 369.0995.

1. 3. Reactions of *N*-Boc protected indol-2-ylmethyl derivatives with active nitroarenes (*one-pot* procedure).

General procedure.

Nitroarene (1.2 mmol) and the precursor of the carbanion (1 mmol) were dissolved in 5 mL of appropriate solvent (MeCN or DMF). The resulting mixture was stirred at room temperature until dissolution, then were added TMSCl (6 mmol) – in one portion and DBU (6 equiv) – dropwise (during 1 min). The reaction vial was stoppered and the mixture stayed without stirring at room temperature usually by several days – progress of the reaction was examined by tlc. In many cases quinoline derivatives precipitated out and were filtered off. In these cases, the solid was washed with chilled MeCN. After completion of the reaction the mixture, after separating precipitated solid – if any, was poured onto mixture of saturated aqueous NH_4Cl solution (25 mL) and water (5 mL), extracted with EtOAc (5 × 25 mL), the extract was washed with brine (50 mL), dried over Na_2SO_4 and evaporated. The crude product was separated using appropriative solvent (or mixture of solvents) on a chromatography column.

The following compounds were obtained:

Bis(*tert*-butyl) 8*H*-benzo[*h*]indolo[2,3-*b*]quinoline-7,8-dicarboxylate (14)

Column chromatography (x 2) (silica gel, 1st: EtOAc- hexane: 1:50, then 1:20; 2nd: toluene:hexane 1:1, then toluene).

Yellow solid, yield 85 mg (18%): mp 189-190.5 °C (EtOAc-hexane; then washed with pentane).

IR (KBr); 3050, 3006, 2977, 2933, 1953, 1893, 1843, 1809, 1737, 1722, 1627, 1611, 1583, 1570, 1518, 1497, 1461, 1438, 1407, 1393, 1369, 1316, 1289, 1250, 1216 cm^{-1}.

^{1}H NMR (500 MHz, $CDCl_3$) δ = 1.78 (s, 9H), 1.81 (s, 9H), 7.51 (t, *J* = 7.5 Hz, 1H), 7.63 (td, *J* = 7.8, 4.5 Hz, 1H), 7.71 (td, *J* = 7.4, 1.0 Hz, 1H), 7.88 (d, *J* = 9.2 Hz, 1H), 7.93 (d, *J* = 8.4 Hz, 1H), 7.79 (td, *J* = 7.6, 1.0 Hz, 1H), 8.15 (d, *J* = 8.4 Hz, 1H), 8.29 (d, *J* = 9.2 Hz, 1H), 8.61 (d, *J* = 7.6 Hz, 1H), 9.59 (d, *J* = 8.2 Hz, 1H).

^{13}C NMR (125 MHz, $CDCl_3$) δ = 28.47, 28.60, 83.51 84.98, 116.24, 121.52, 121.86, 122.62, 123.86, 124.91, 125.35, 126.30, 126.98, 127.04, 127.50, 128.00, 128.07, 130.10, 131.50, 132.72, 141.19, 143.84, 145.81, 150.84, 165.19.

HRMS (ESI): m/z calcd for $C_{29}H_{29}N_2O_4^+$: 469.2127; found: 469.2108.

Bis(*tert*-butyl) 6*H*-indolo[3,2-*b*][1,7]naphthiridine-5,6-dicarboxylate (15)

Column chromatography (silica gel, EtOAc-toluene: 1:10, then 1:5, then 1:2).

Yellow solid, yield 167 mg (40%): mp 168-170 °C (EtOAc-hexane; then washed with pentane).

IR (KBr) 3032, 3005, 2974, 2933, 2909, 2877, 1739 (CO), 1725 (CO), 1620, 1592, 1572, 1484, 1461, 1387, 1369, 1343, 1311, 1294, 1242, 1208 cm^{-1}.

^{1}H NMR (500 MHz, $CDCl_3$) δ = 1.77 (s, 9H), 1.78 (s, 9H), 7.50 (t, *J* = 7.5 Hz; 1H), 7.67 (td, *J* = 7.8, 1.1 Hz; 1H), 8.11 – 8.15 (m, 2H), 8.47 (d, *J* = 7.6 Hz, 1H), 8.65 (d, *J* = 5.8 Hz, 1H), 9.66 (s, 1H).

^{13}C NMR (125 MHz, $CDCl_3$) δ = 28.40, 28.56, 83.97, 85.66, 116.31, 117.53, 121.99, 123.55, 124.35, 124.38, 127.16, 128.99, 131.53, 140.73, 142.28, 143.37, 149.70, 150.50, 154.09, 163.93.

HRMS (ESI) m/z calcd for $C_{24}H_{26}N_3O_4^+$: 420.1923.; found: 420.1917.

Bis(*tert*-butyl) 8*H*-indolo[3,2-*b*][1,7]phenanthroline-7,8-dicarboxylate (16).

A pale yellow solid was collected by filtration and washed with MeCN; the product that remained in the filtrate was separated by column chromatography (silica gel, EtOAc-hexane, 1:10).

Yield 194 mg (41%); mp 179-180 °C (solid was only washed with MeCN).

IR (KBr) 3060, 2975, 2929, 2251, 1737 (CO), 1723 (CO), 1612, 1584, 1514, 1497, 1477, 1458, 1415, 1393, 1368, 1349, 1319, 1252, 1217 cm^{-1}.

^{1}H NMR (500 MHz, $CDCl_3$) δ = 1.78 (s, 9H), 1.81 (s, 9H), 7.51 (td, *J* = 7.4, 0.6 Hz; 1H), 7.65 (td, *J* = 7.8, 1.4 Hz; 1H), 7.68 (dd, *J* = 8.2, 4.4 Hz; 1H), 8.13 (d, *J* = 9.9, Hz; 1H), 8.15 (d, *J* = 8.3, Hz; 1H), 8.46 (d, *J* = 9.5 Hz; 1H), 8.53 (dd, *J* = 7.5, 0.4 Hz; 1H), 9.06 (dd, *J* = 4.3, 1.7 Hz; 1H), 9.81 (dd, *J* = 8.3, 1.0 Hz; 1H).

^{13}C NMR (125 MHz, $CDCl_3$) δ 28.45, 28.61, 83.84, 85,21, 116.29, 121.40, 121.58, 121.89, 123.97, 125.04, 126.34, 126.46, 127.03, 127.33, 128.91, 130.45, 133.17, 141.34, 143.16, 146.63, 148.66, 150.75, 150.98, 164.86.

HRMS (ESI) m/z calcd for $C_{28}H_{28}N_3O_4^+$: 470.2080; found: 470.2071.

Bis(*tert*-butyl) 12*H*-indolo[3,2-*j*][1,7]phenanthroline-12,13-dicarboxylate (17).

Column chromatography (silica gel, EtOAc- toluene: 1:10, then 1:5).

Brown solid, yield 63 mg (13%): mp 176-178 °C (EtOAc-hexane; then washed with pentane).

IR (KBr); 3125, 3060, 3022, 2976, 2934, 1929, 1899, 1758 (CO), 1714 (CO), 1630, 1613, 1589, 1513, 1498, 1482, 1459, 1405, 1939, 1367, 1315, 1300, 1249, 1217 cm^{-1}.

^{1}H NMR (500 MHz, $CDCl_3$) δ = 1.70 (s, 9H), 1.76 (s, 9H), 7.49 (t, *J* = 7.6 Hz, 1H), 7.51 (dd, *J* = 8.4, 4.5 Hz, 1H), 7.65 (t, *J* = 7.5 Hz, 1H), 8.11 (d, *J* = 8.4 Hz, 1H), 8.27 (d, *J* = 9.4 Hz, 1H), 8.35 (d, *J* = 9.4 Hz, 1H), 8.44 (d, *J* = 7.6 Hz, 1H), 8.99 (d, *J* = 4.2 Hz, 1H), 9.01 (d, *J* = 8.9 Hz, 1H).

^{13}C NMR (125 MHz, $CDCl_3$) δ = 28.44, 28.36, 85.10, 85.29, 116.00, 119.58, 119.79, 121.48, 124.12, 124.45, 125.15, 125.80, 128.40, 130.12, 130.75, 132.95, 136.79, 142.09, 145.69, 147.74, 147.97, 148.55, 150.61, 166.65.

HRMS (ESI): m/z calcd for $C_{28}H_{28}N_3O_4^+$: 470.2080; found: 470.2070.

***tert*-Butyl 13-cyano-12*H*-indolo[3,2-*b*][4,7]phenanthroline-12-carboxylate (18).**

A pale yellow solid was collected by filtration and washed with MeCN; the product that remained in the filtrate was separated by column chromatography (silica gel, EtOAc-toluene, 1:20, then 1:5).

Yield 285 mg (72%): mp > 305 °C (only washed with MeCN).

IR (KBr) 3028, 2983, 2932, 2226 (CN), 1967, 1928, 1803, 1741 (CO), 1614, 1585, 1505, 1492, 1482, 1456, 1410, 1380, 1365, 1346, 1311, 1289, 1254, 1234, 1209 cm^{-1}.

^{1}H NMR (500 MHz, $CDCl_3$) δ = 1.85 (s, 9H), 7.52 (t, *J* = 7.5 Hz, 1H), 7.67 – 7.71 (m, 2H), 8.24 (d, *J* = 8.4 Hz, 1H), 8.27 (d, *J* = 9.5 Hz; 1H), 8.38 (d, *J* = 9.3 Hz; 1H), 9.07 (dd, *J* = 4.2, 1.2 Hz; 1H), 10.06 (d, *J* = 7.8 Hz, 1H).

^{13}C NMR (125 MHz, $CDCl_3$) δ = 28.21, 86.98, 101.78, 115.82, 116.88, 121.40, 121.74, 121.89, 121.74, 123.65, 124.17,124.70, 131.44, 132.17, 132.37, 133.27, 133.92, 142.62, 145.37, 147.68, 148.51, 149.35, 150.88.

HRMS (ESI) m/z calcd for $C_{24}H_{19}N_4O_2^+$: 395.1508; found: 395.1505.

***tert*-Butyl 13-cyano-12*H*-indolo[3,2-*b*][1,5]phenanthroline-12-carboxylate (19).**

A beige solid was almost quantitatively collected by filtration and washed with MeCN.

Yield 201 mg (51%): mp > 305 °C (solid was only washed with MeCN).

IR (KBr) 3059, 2985, 2934, 2224 (CN), 1972, 1948, 1875, 1799, 1728 (CO), 1683, 1614, 1599, 1561, 1504, 1494, 1482, 1456, 1406, 1393, 1367, 1345, 1295, 1254, 1225 cm^{-1}.

^{1}H NMR (500 MHz, $CDCl_3$) δ = 1.83 (s, 9H), 7.54 (t, *J* = 7.5 Hz, 1H), 7.66 (dd, *J* = 7.7, 4.3 Hz, 1H), 7.72 (td, *J* = 7.7, 1.3 Hz, 1H), 8.00 (d, *J* = 9.0 Hz, 1H), 8.26 (d, *J* = 9.0 Hz, 1H), 8.28 – 8.32 (m, 2H), 8.47 (d, *J* = 7.7 Hz, 1H), 9.17 (dd, *J* = 4.2, 1.7 Hz, 1H). 7.67 – 7.71 (m, 2H), 8.24 (d, *J* = 8.4 Hz, 1H), 8.27 (d, *J* = 9.5 Hz; 1H), 8.38 (d, *J* = 9.3 Hz; 1H), 9.07 (dd, *J* = 4.2, 1.2 Hz; 1H), 10.06 (d, *J* = 7.8 Hz, 1H).

^{13}C NMR (125 MHz, $CDCl_3$) δ = 28.15, 86.72, 103.39, 115.70, 116.18, 121.74, 123.12, 123.61, 123.80, 124.47, 126.68, 128.79, 128.98, 131.42, 134.49, 135.67, 143.11, 144.85, 146.94, 148.51, 148.53, 149.40.

HRMS (ESI) m/z calcd for $C_{24}H_{19}N_4O_2^+$: 395.1508; found: 395.1496.

tert-Butyl 3-_n_-butyl-12-cyano-11_H_-indolo[3,2-_b_]pyrazolo[4,5-_f_]quinoline-11-carboxylate (20).

A pale yellow solid was collected by filtration and washed with MeCN; the product that remained in the filtrate was separated by column chromatography (silica gel, toluene, then EtOAc-toluene, 1:10).

Yield 270 mg (61%): mp 298-300 °C (solid was only washed with MeCN).

IR (KBr); 3129, 3063, 2956, 2929, 2873, 2225 (CN), 1905 (1734 (CO), 1615, 1604, 1522, 1463, 1426, 1392, 1365, 1328, 1295, 1253, 1223 cm^{-1}.

^{1}H NMR (500 MHz, $CDCl_3$) δ = 0.98 (t, *J* = 7.4 Hz, 3H), 1.41 (sex, *J* = 7.4 Hz, 2H), 1.83 (s, 9H), 2.01 (quin, *J* = 7.3 Hz, 2H), 4.53 (t, *J* = 7.1 Hz, 2H), 7.51 (t, *J* = 7.5 Hz, 1H), 7.66 (t, *J* = 7.7 Hz, 1H), 7.81 (d, *J* = 9.2 Hz, 1H), 8.18 (d, *J* = 9.2 Hz, 1H), 8.25 (d, *J* = 8.3 Hz, 1H), 8.42 (d, *J* = 7.6 Hz, 1H), 9.29 (s, 1H).

^{13}C NMR (125 MHz, $CDCl_3$) δ = 13.63, 20.06, 28.16, 32.20, 49.23, 86.99, 100.37, 113.64, 115.65, 116.64, 121.26, 121.33, 123.57, 123.92, 124.45, 128.89, 130.64, 131.78, 134.41, 137.95, 141.83, 142.76, 144.83, 149.56.

HRMS (ESI): m/z calcd for $C_{26}H_{26}N_5O_2^+$: 440.2087; found: 440.2072.

Copies of ^{1}H and ^{13}C NMR spectra

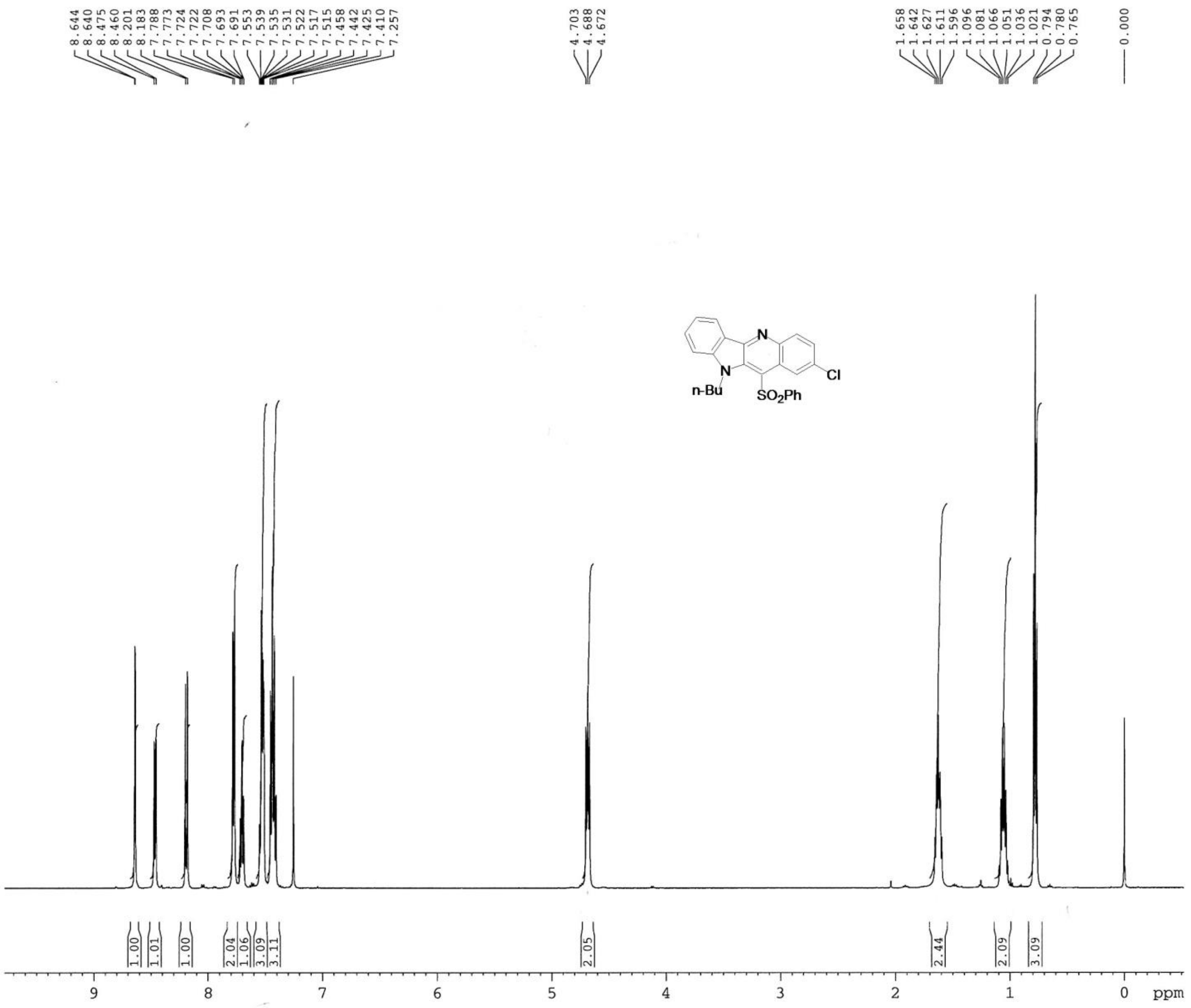

^{1}H NMR spectrum of 10-*n*-butyl-2-chloro-11-(phenylsulfonyl)-10*H*-indolo[3,2-*b*]quinoline (3).

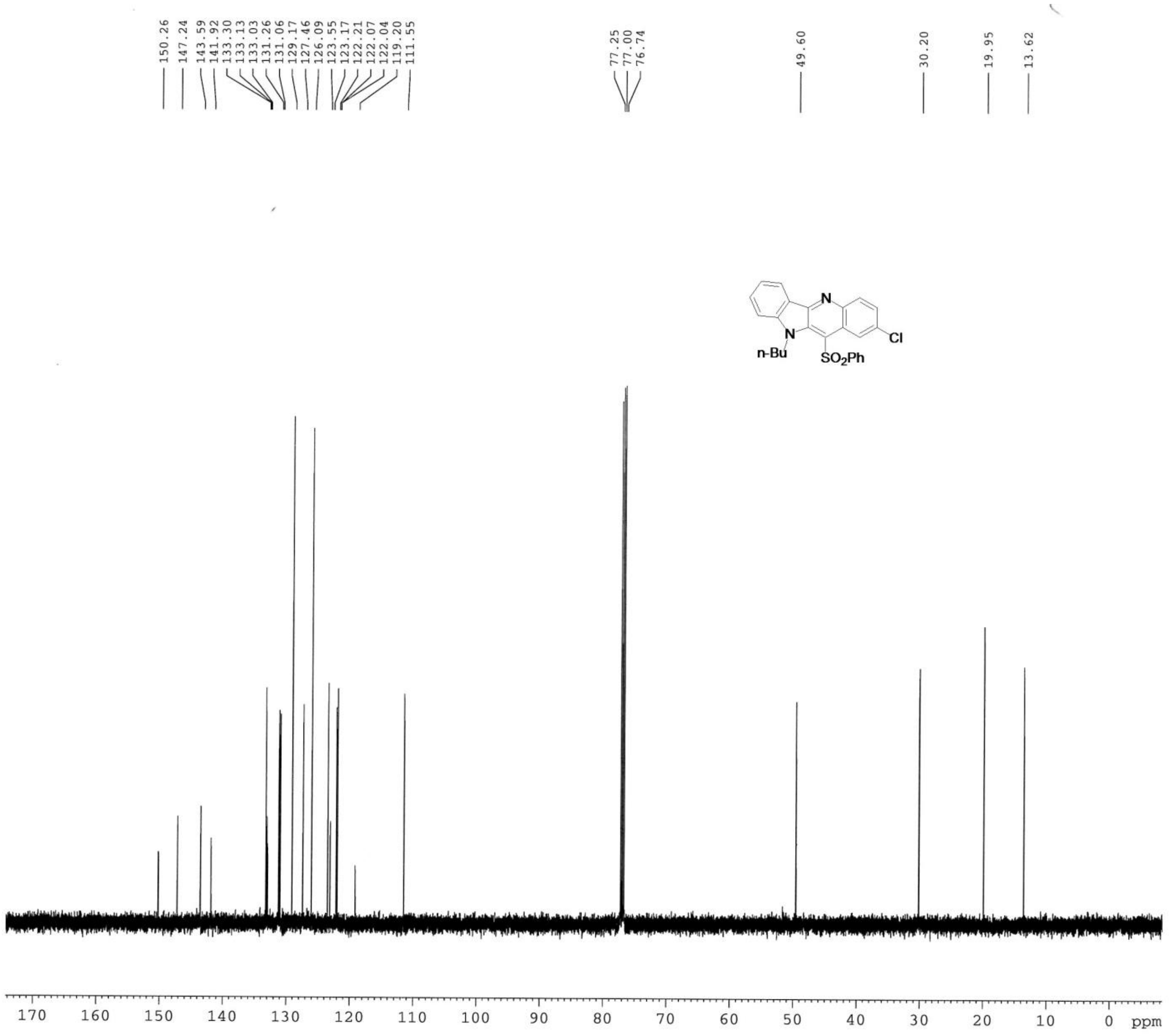

^{13}C NMR spectrum of 10-*n*-butyl-2-chloro-11-(phenylsulfonyl)-10*H*-indolo[3,2-*b*]quinoline (3)

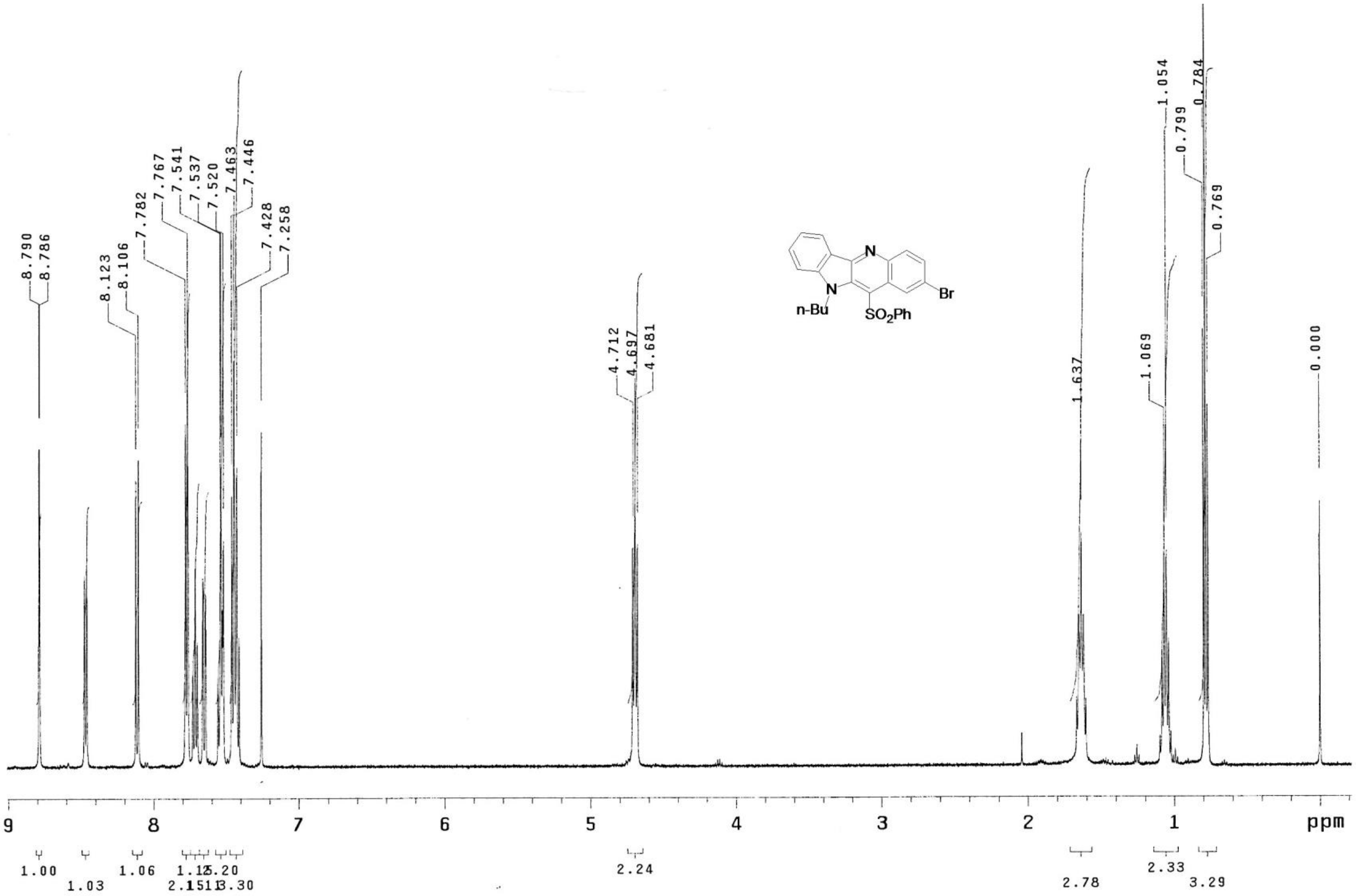

^{1}H NMR spectrum of 2-bromo-10-*n*-butyl-11-(phenylsulfonyl)-10*H*-indolo[3,2-*b*]quinoline (4)

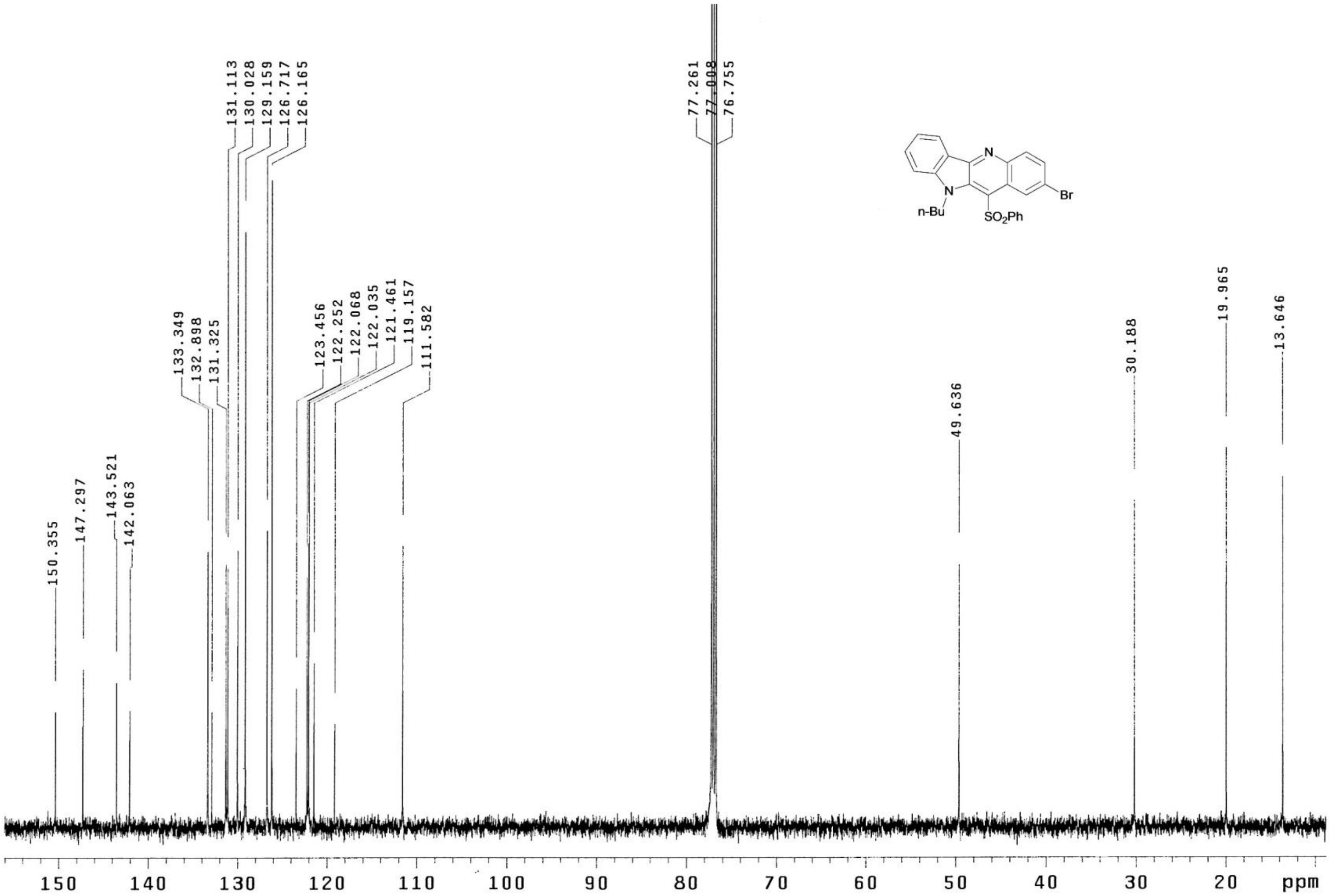

^{13}C NMR spectrum of 2-bromo-10-*n*-butyl-11-(phenylsulfonyl)-10*H*-indolo[3,2-*b*]quinoline (4).

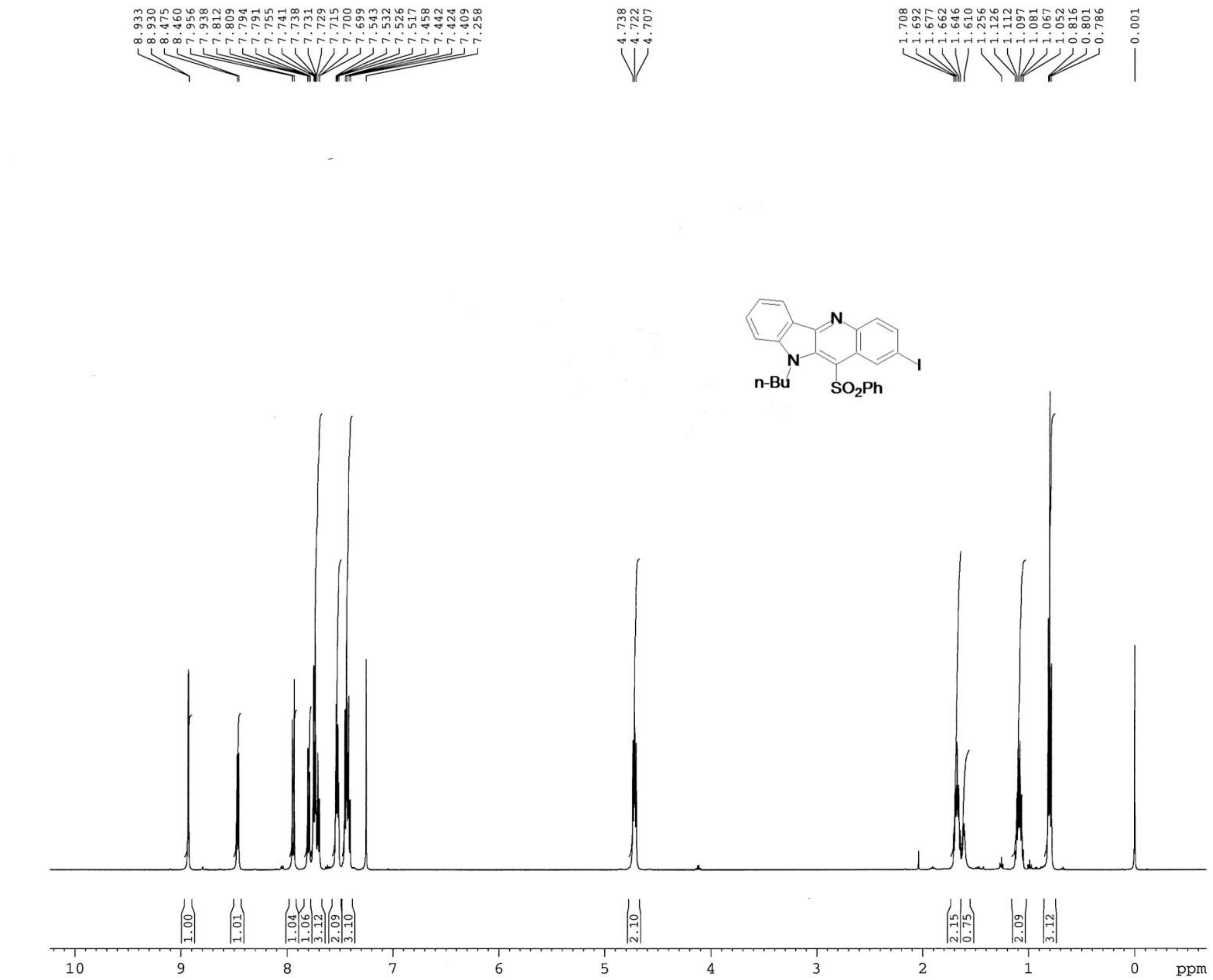

^{1}H NMR spectrum of 10-*n*-butyl-2-iodo-11-(phenylsulfonyl)-10*H*-indolo[3,2-*b*]quinoline(5).

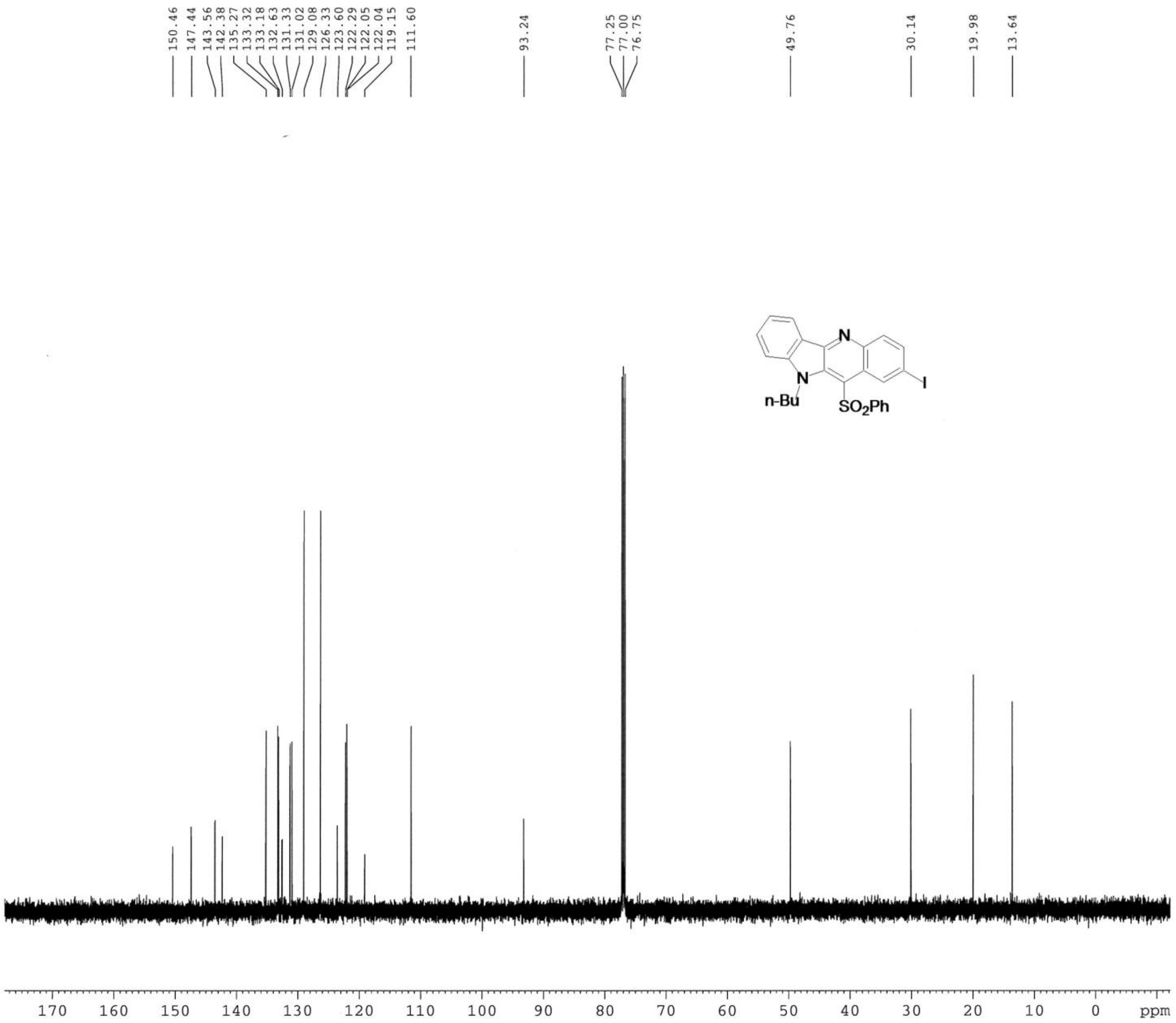

^{13}C NMR spectrum of 10-*n*-butyl-2-iodo-11-(phenylsulfonyl)-10*H*-indolo[3,2-*b*]quinoline (5).

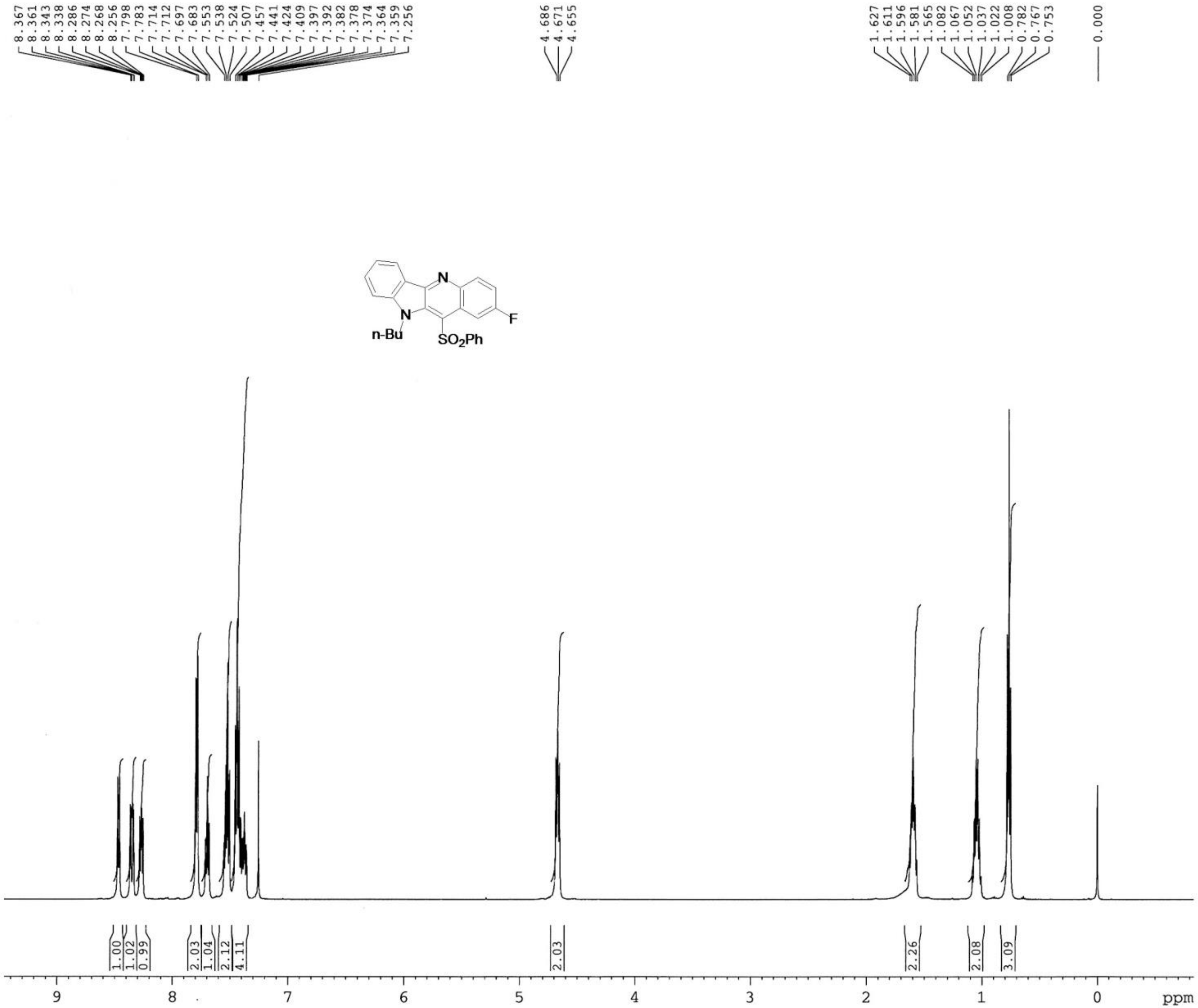

^{1}H NMR spectrum of 10-*n*-butyl-2-fluoro-11-(phenylsulfonyl)-10*H*-indolo[3,2-*b*]quinoline (6).

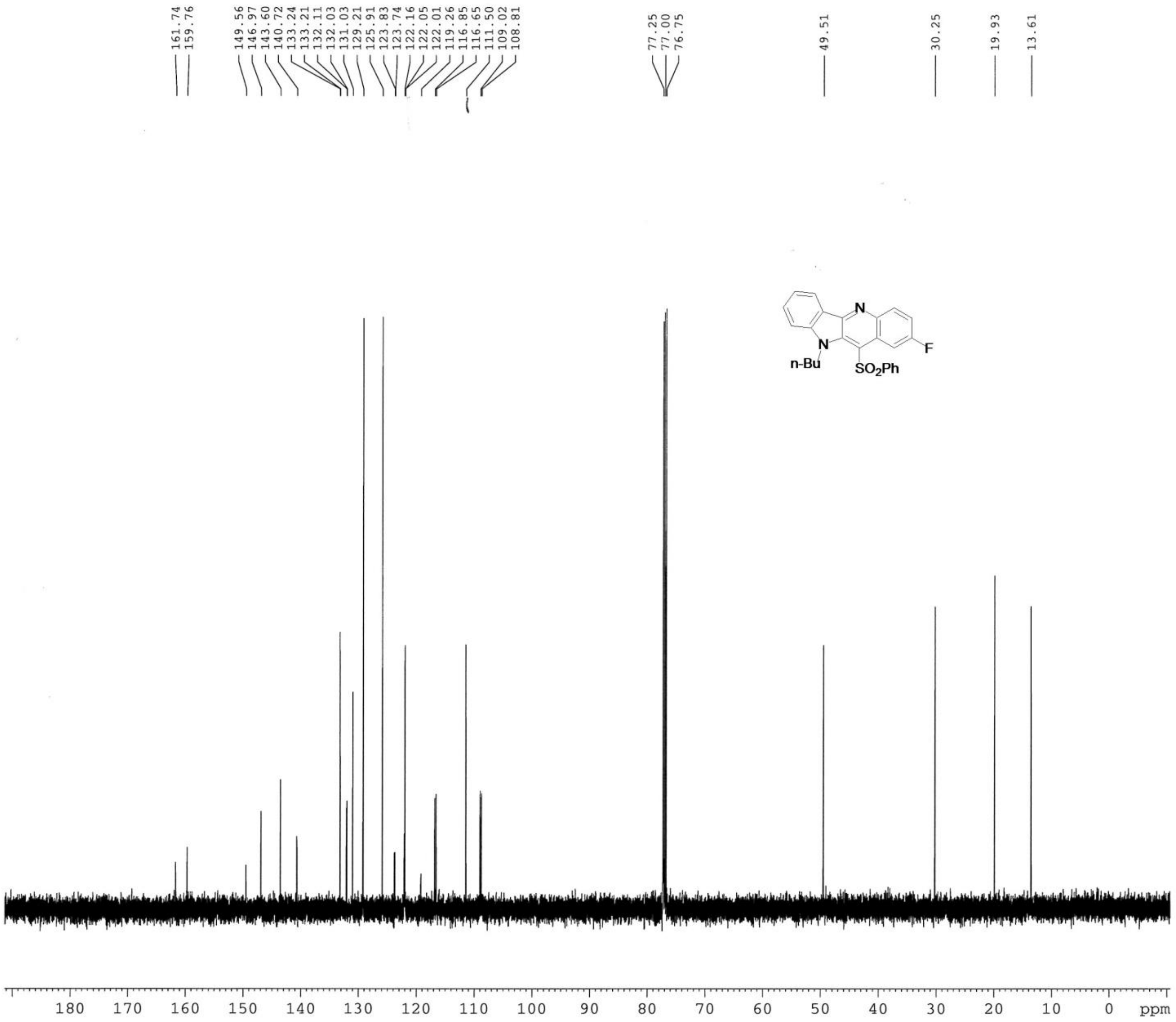

^{13}C NMR spectrum of 10-*n*-butyl-2-fluoro-11-(phenylsulfonyl)indolo[3,2-*b*]quinoline (6).

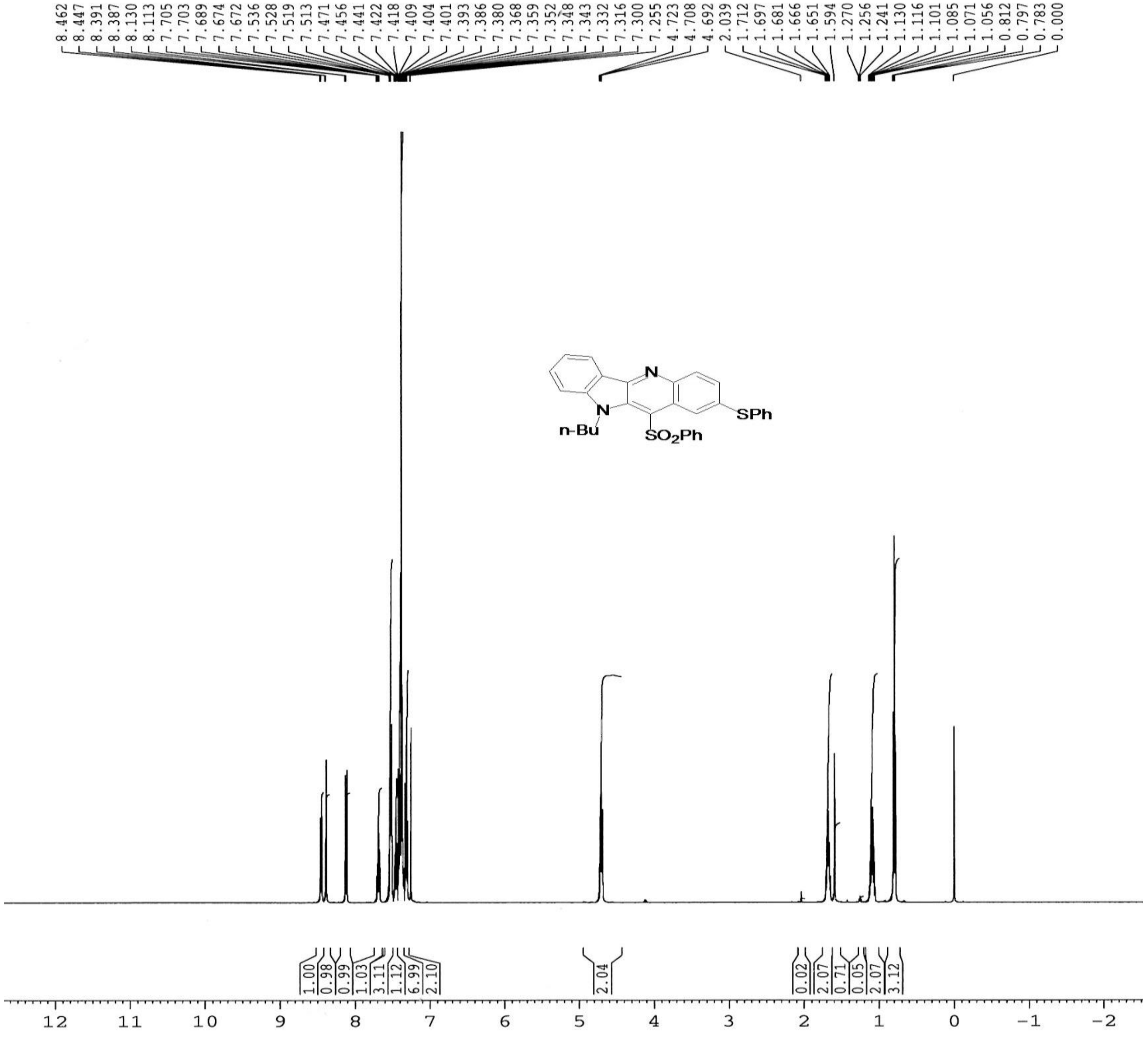

^{1}H NMR spectrum of 10-*n*-butyl-11-(phenylsulfonyl)-2-(phenylthio)-10*H*-indolo[3,2-*b*]quinoline (7).

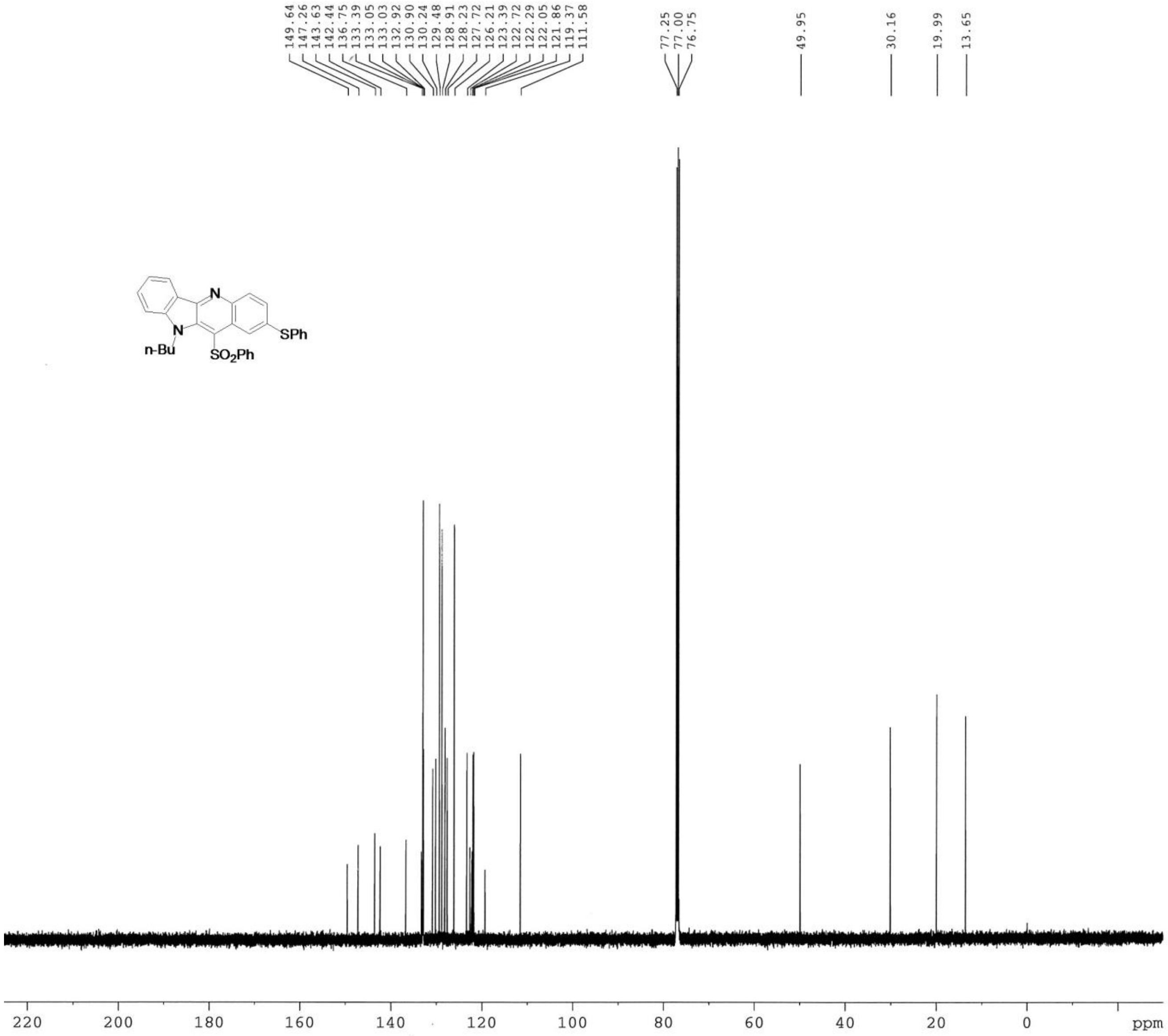

^{13}C NMR spectrum of 10-*n*-butyl-11-(phenylsulfonyl)-2-(phenylthio)indolo[3,2-*b*]quinoline (7).

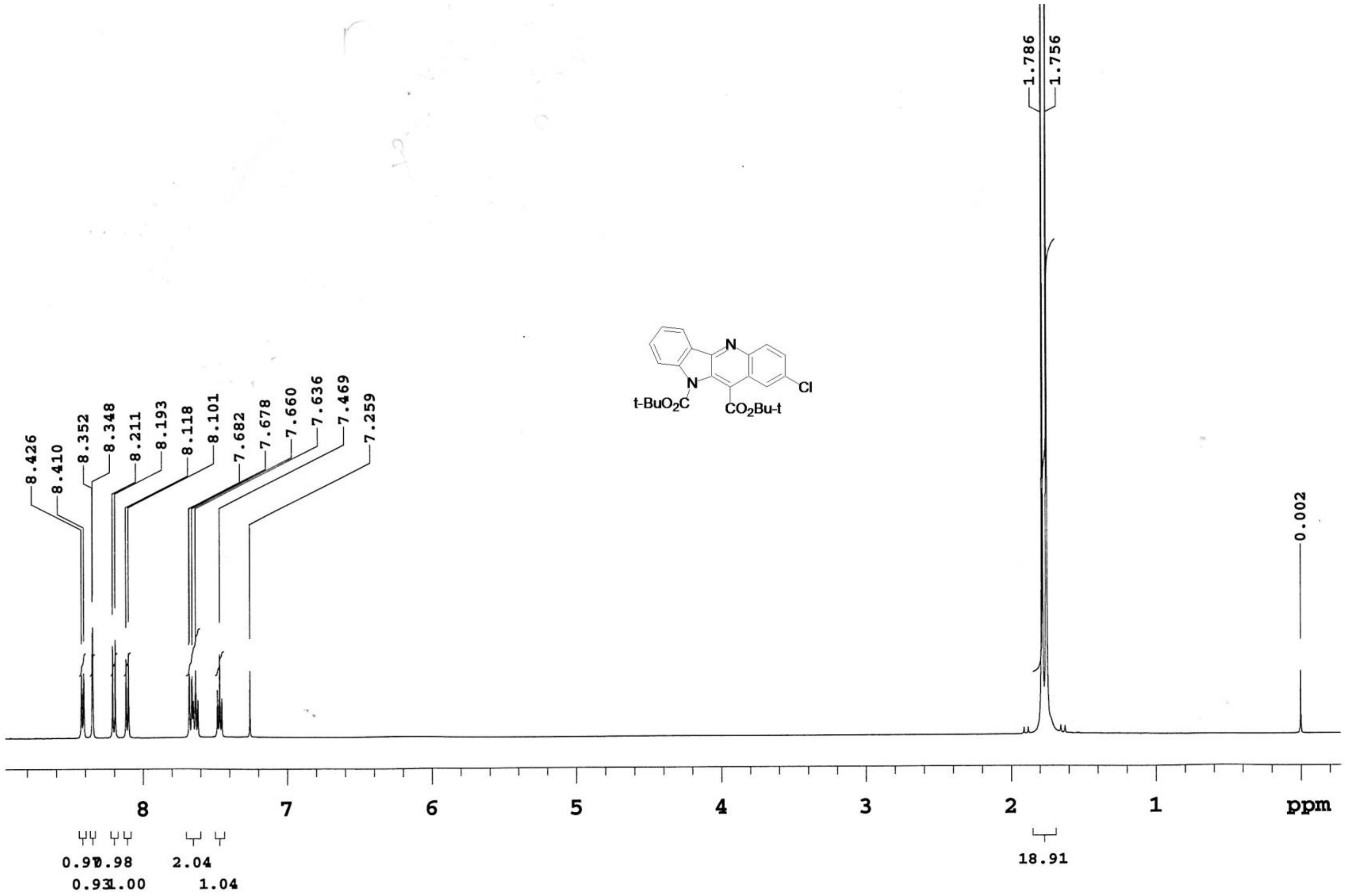

^{1}H NMR spectrum of bis(*tert*-butyl) 2-chloro-10*H*-indolo[3,2-*b*]quinoline-10,11-dicarboxylate (8).

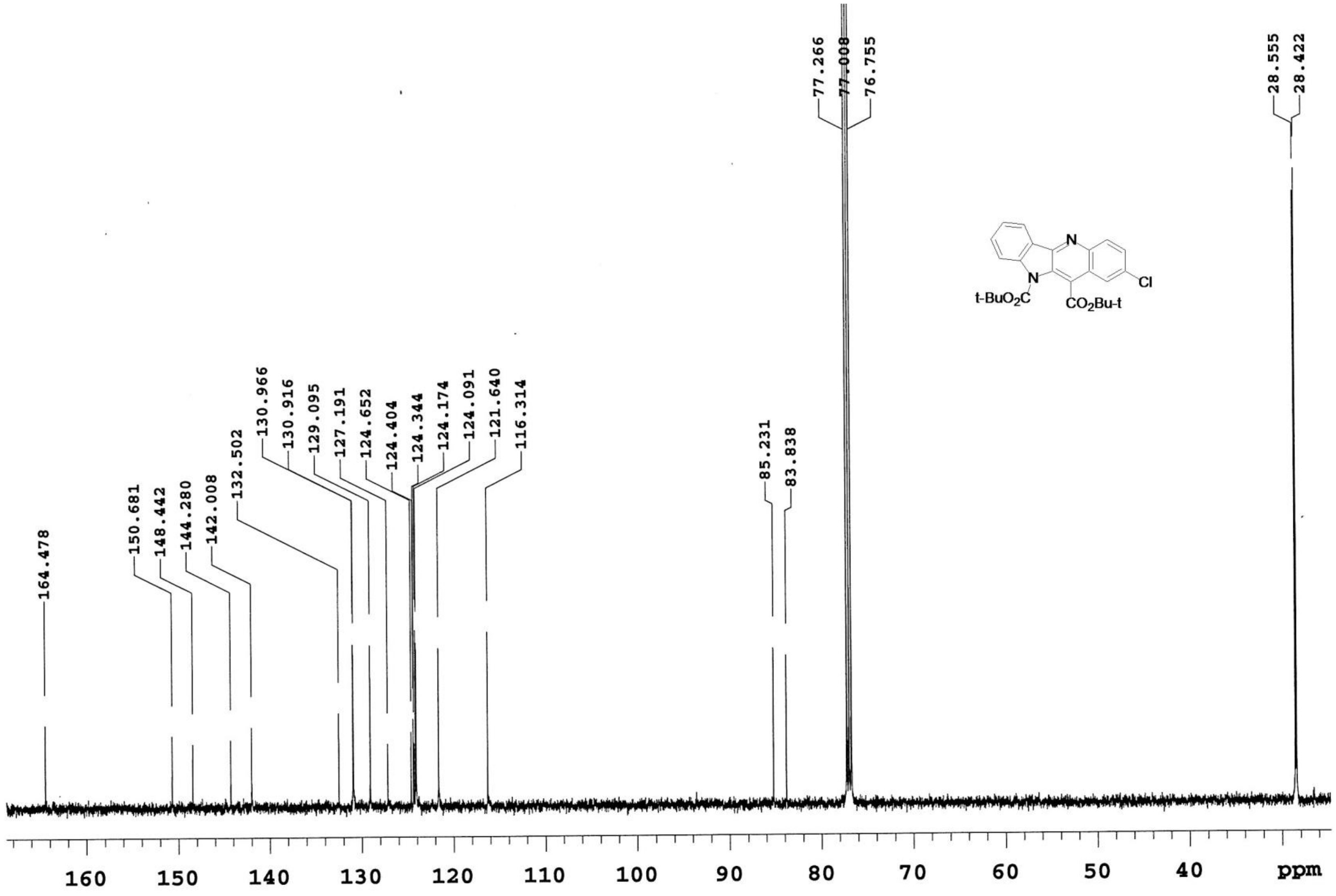

^{13}C NMR spectrum of bis(*tert*-butyl) 2-chloro-10*H*-indolo[3,2-*b*]quinoline-10,11-dicarboxylate (8).

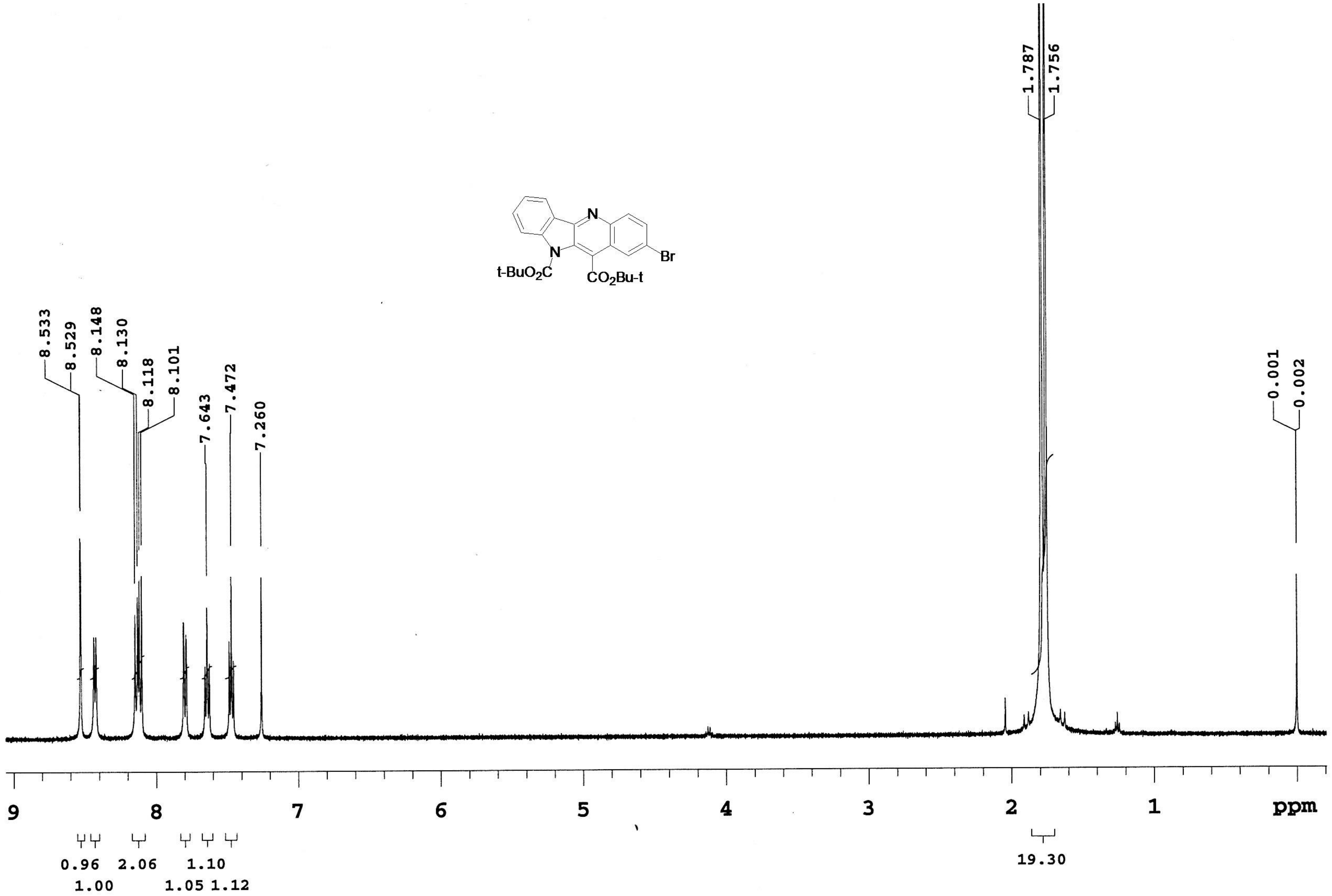

^{1}H NMR spectrum of bis(*tert*-butyl) 2-bromo-10*H*-indolo[3,2-*b*]quinoline-10,11-dicarboxylate (9).

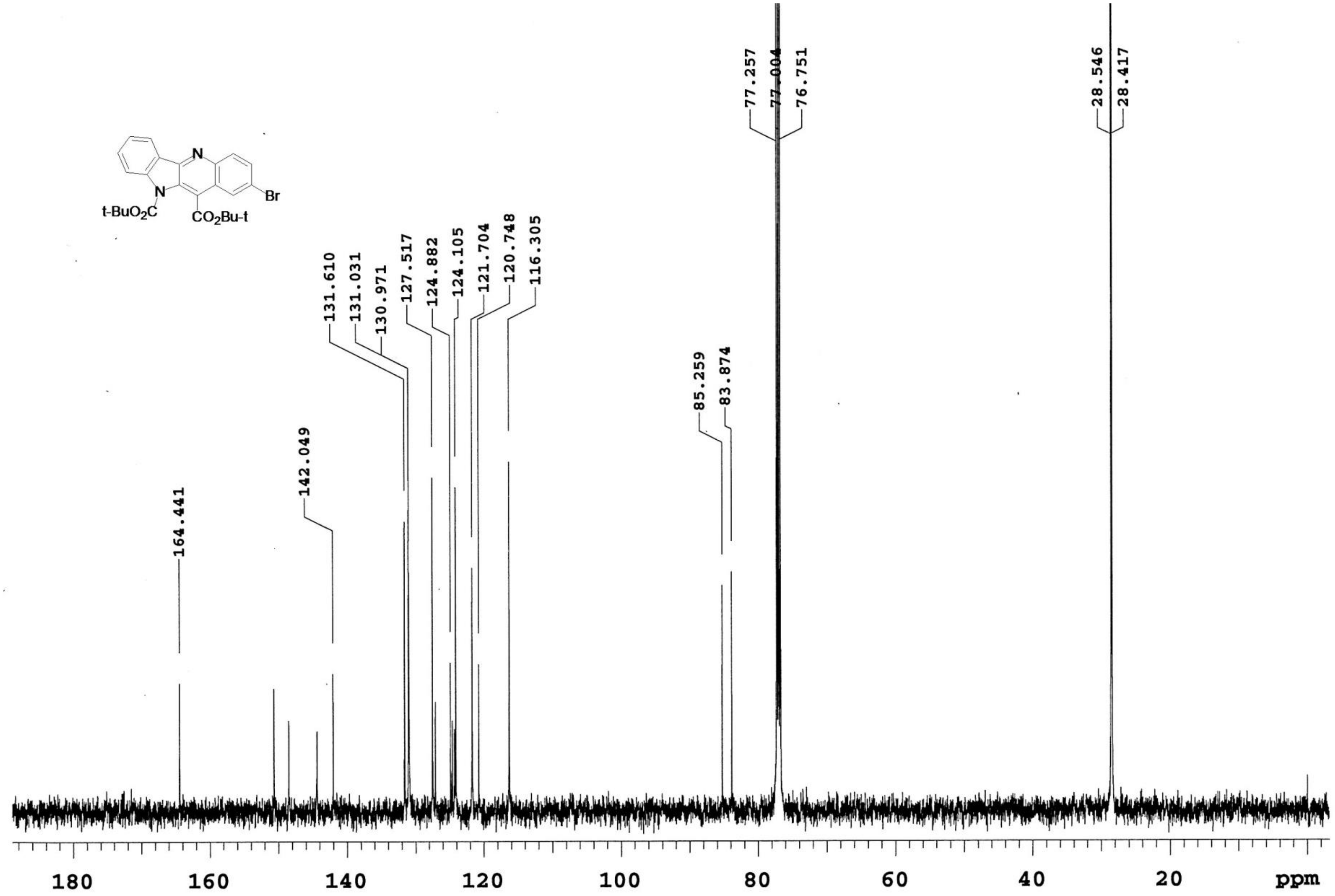

^{13}C NMR spectrum of bis(*tert*-butyl) 2-bromo-10*H*-indolo[3,2-*b*]quinoline-10,11-dicarboxylate (9)).

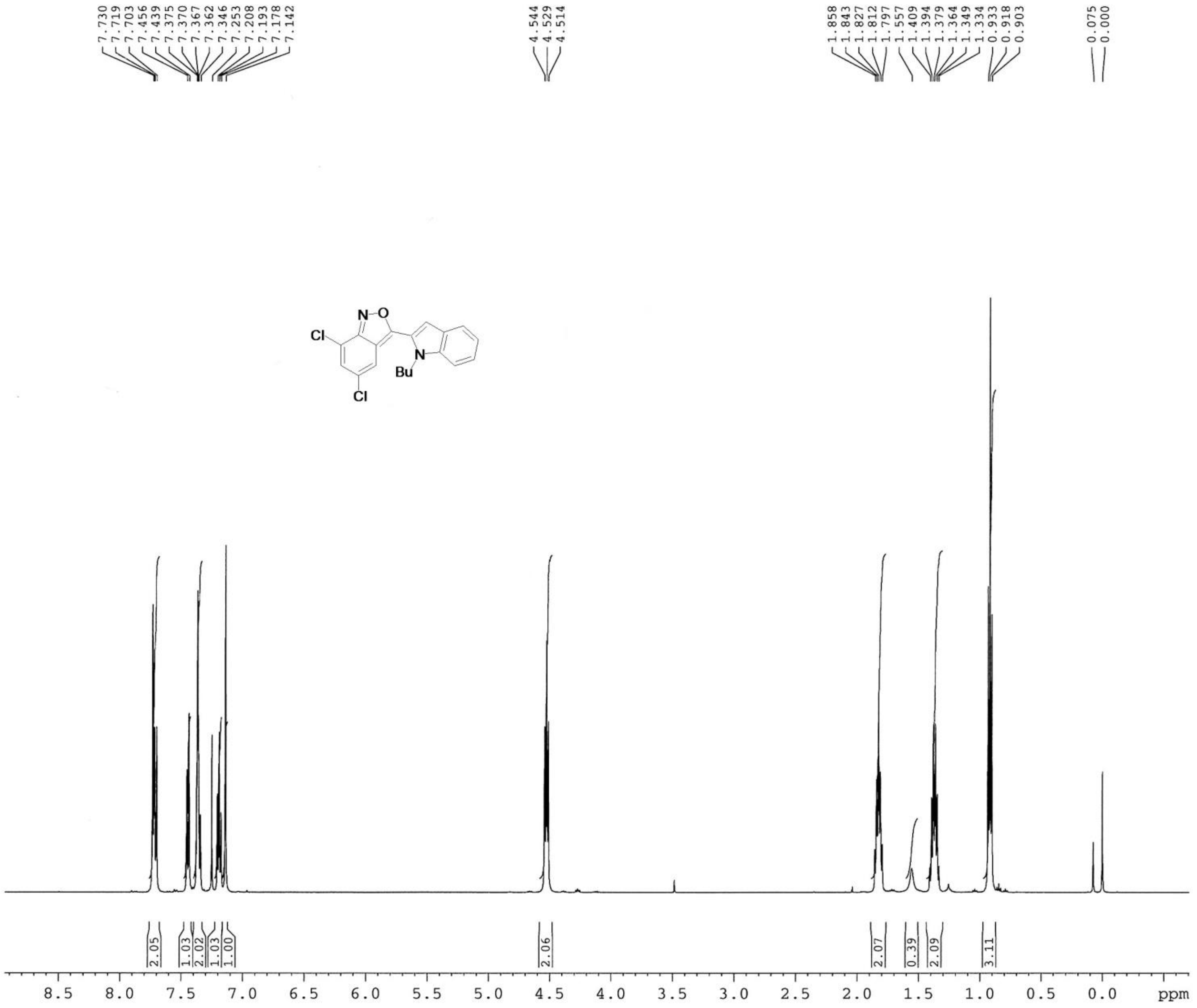

^{1}H NMR spectrum of 3-(1-*n*-butylindol-2-yl)- 5,7-dichloro-2,1-benzisoxazole (10).

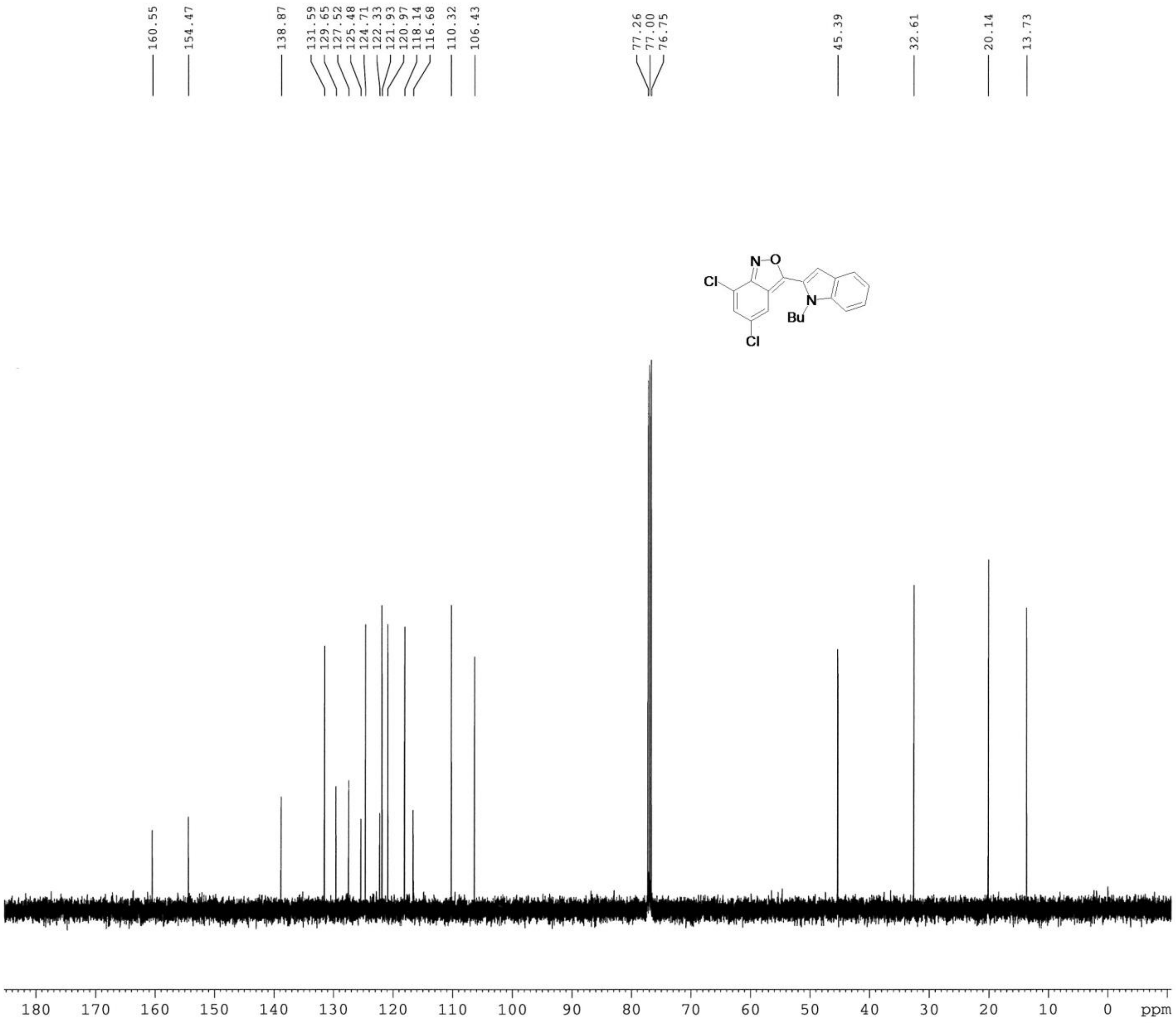

^{13}C NMR spectrum of 3-(1-*n*-butylindol-2-yl)- 5,7-dichloro-2,1-benzisoxazole (10).

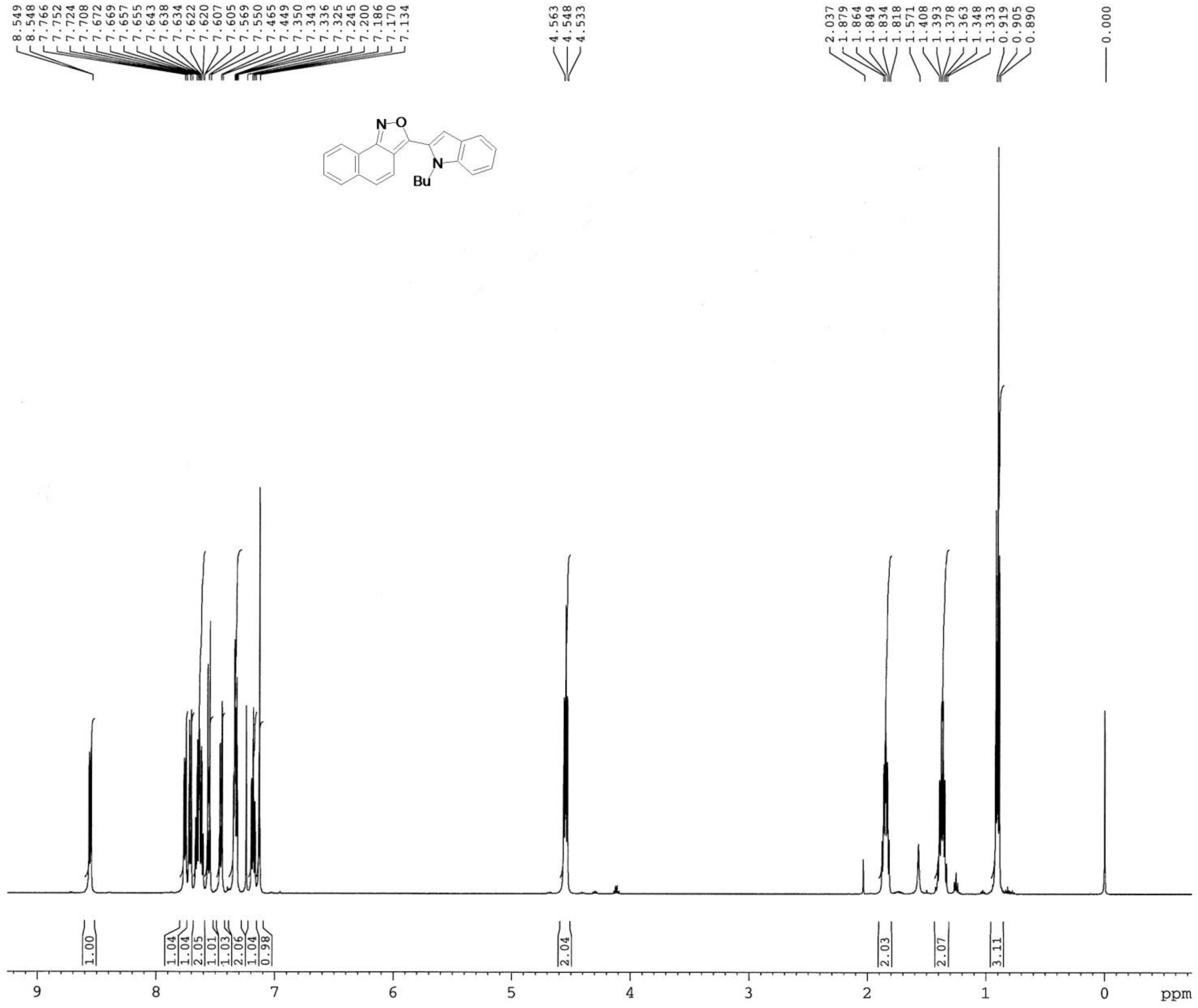

^{1}H NMR spectrum of 1-(1-*n*-butylindol-2-yl)naphth[1,2-*c*][1,2]oxazole (11).

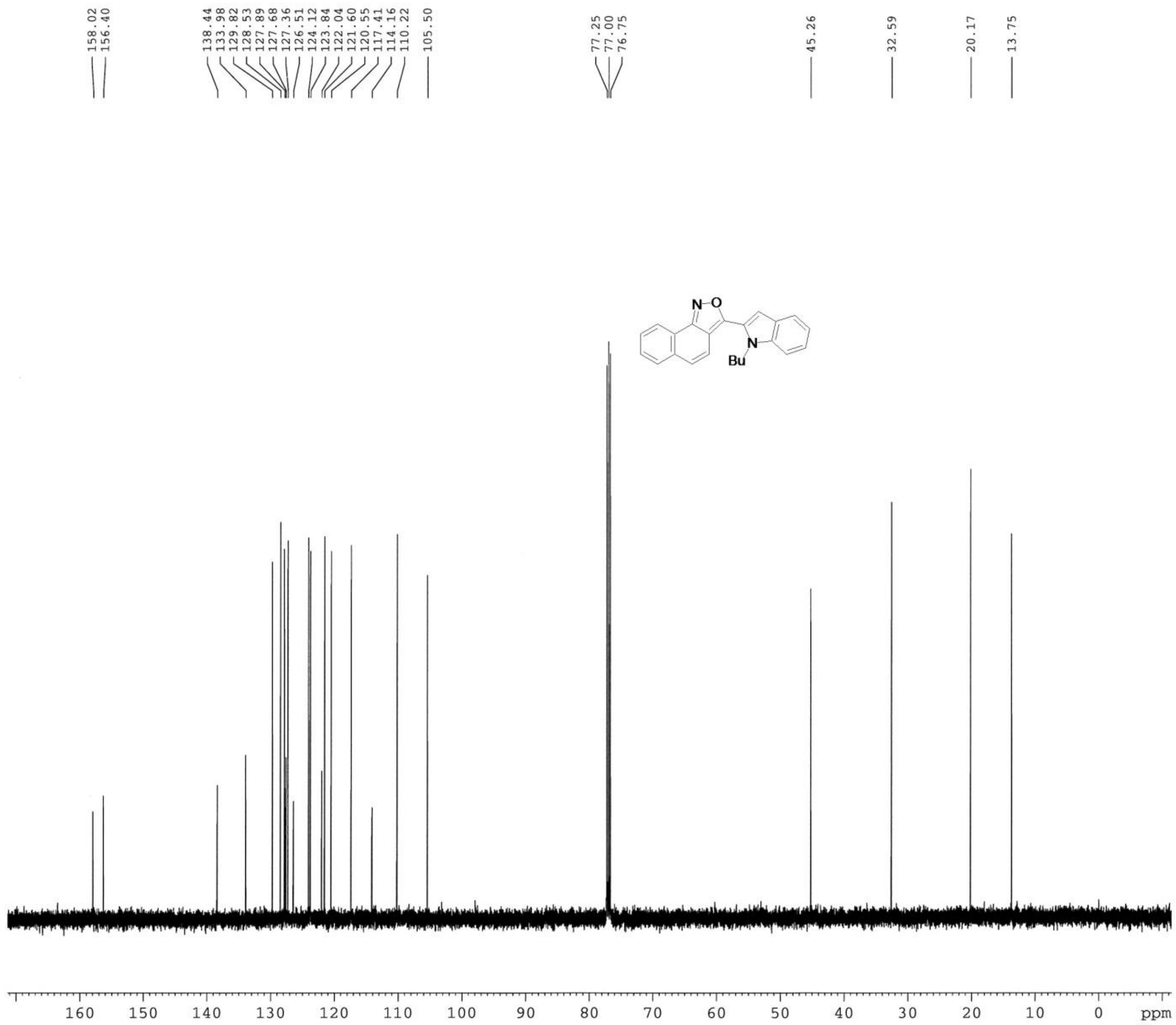

^{13}C NMR spectrum of 1-(1-*n*-butylindol-2-yl)naphth[1,2-*c*][1,2]oxazole (11).

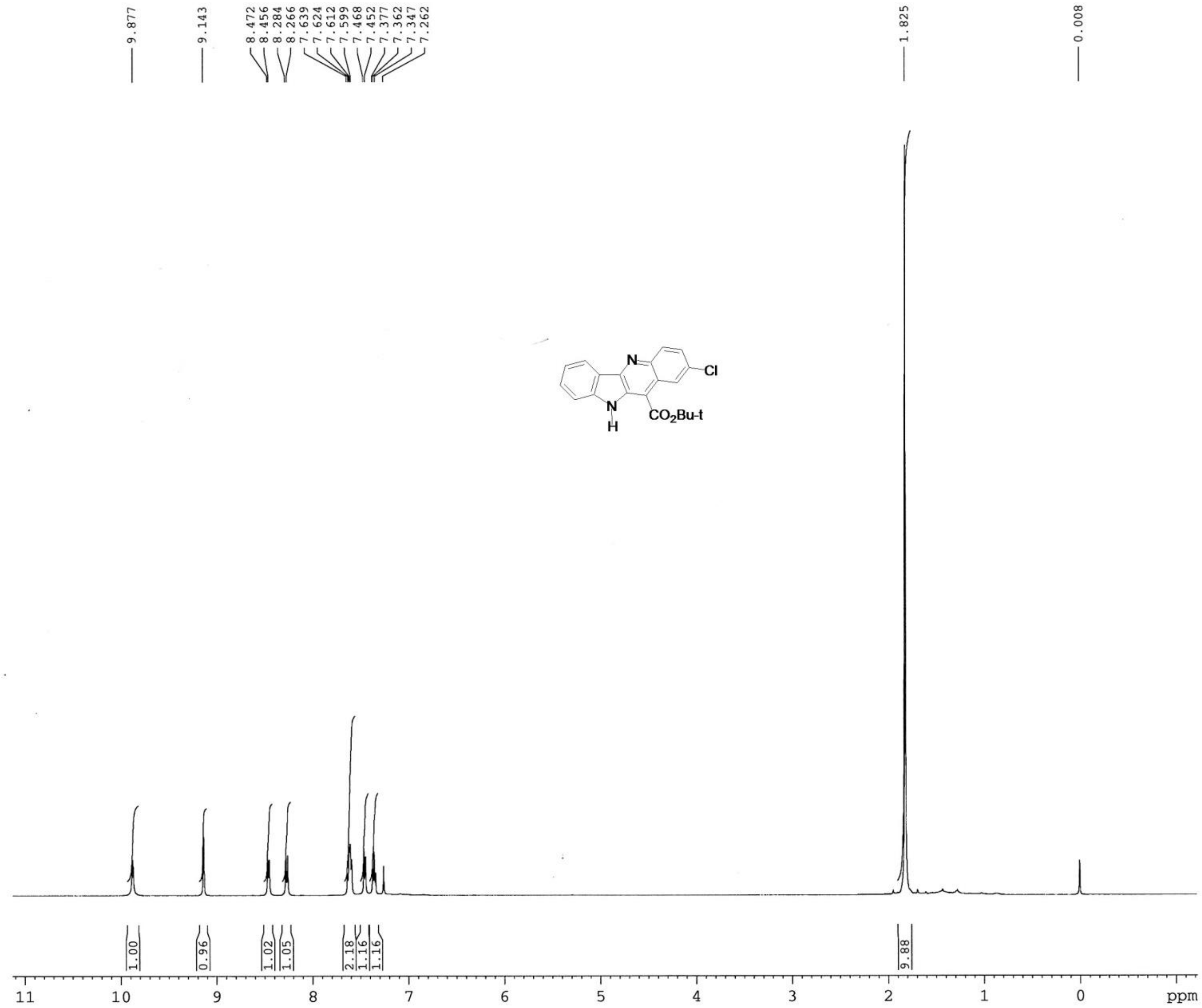

^{1}H NMR spectrum of *tert*-butyl 2-chloro-10*H*-indolo[3,2-*b*]quinoline-11-carboxylate (12).

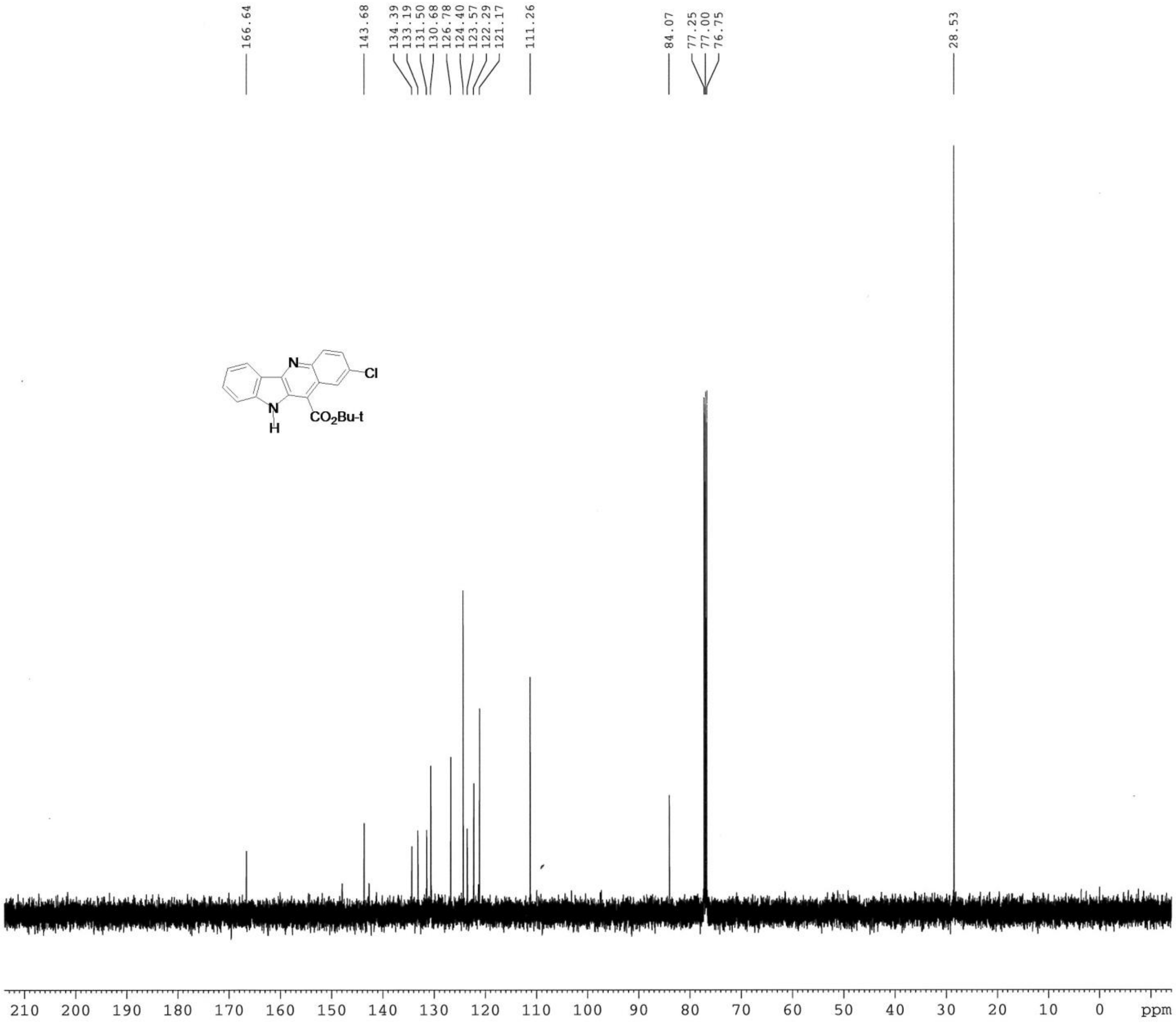

^{13}C NMR spectrum of *tert*-butyl 2-chloro-10*H*-indolo[3,2-*b*]quinoline-11-carboxylate (12).

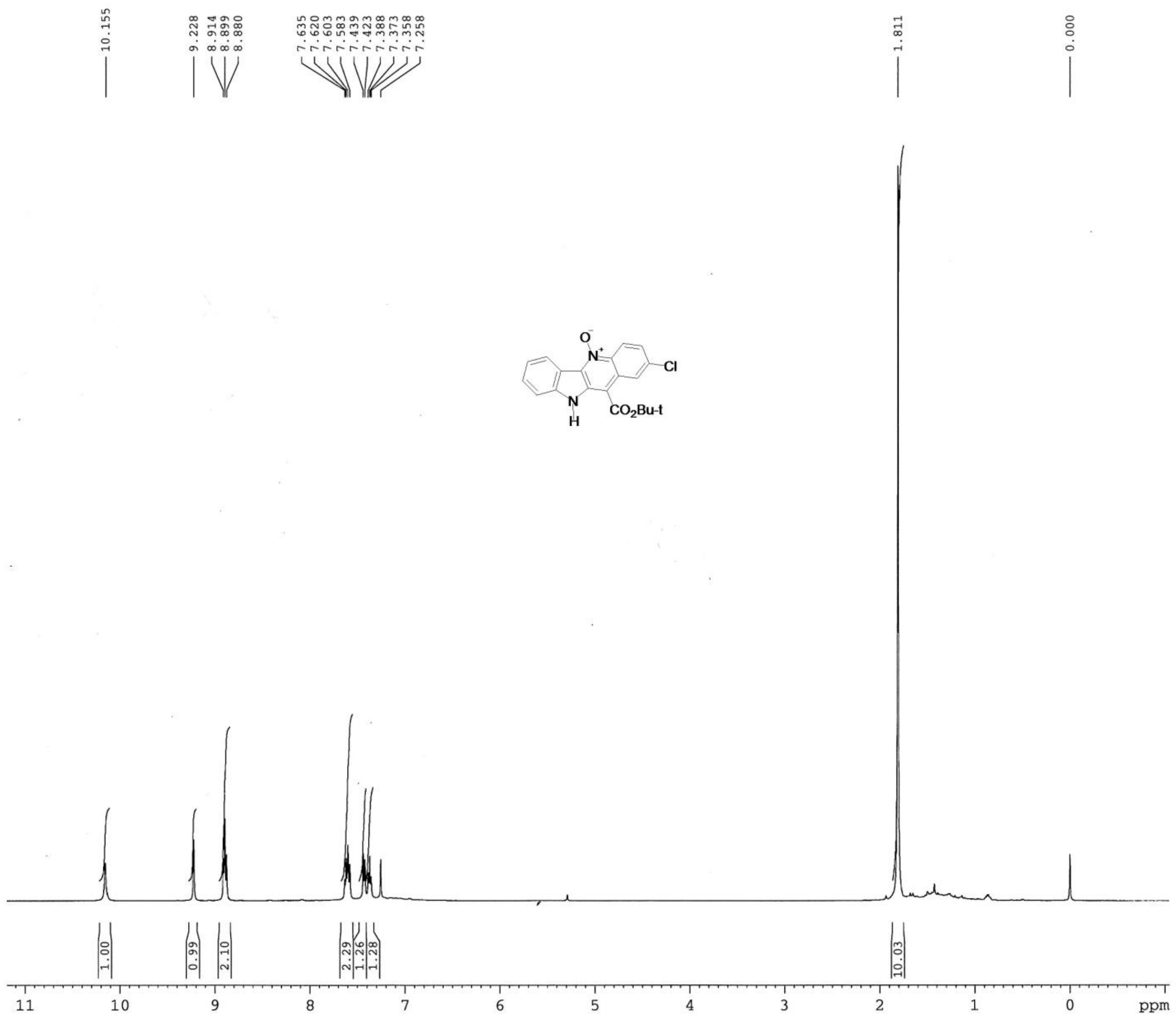

^{1}H NMR spectrum of *tert*-butyl 2-chloro-10*H*-indolo[3,2-*b*]quinoline-11-carboxylate-5-oxide (13).

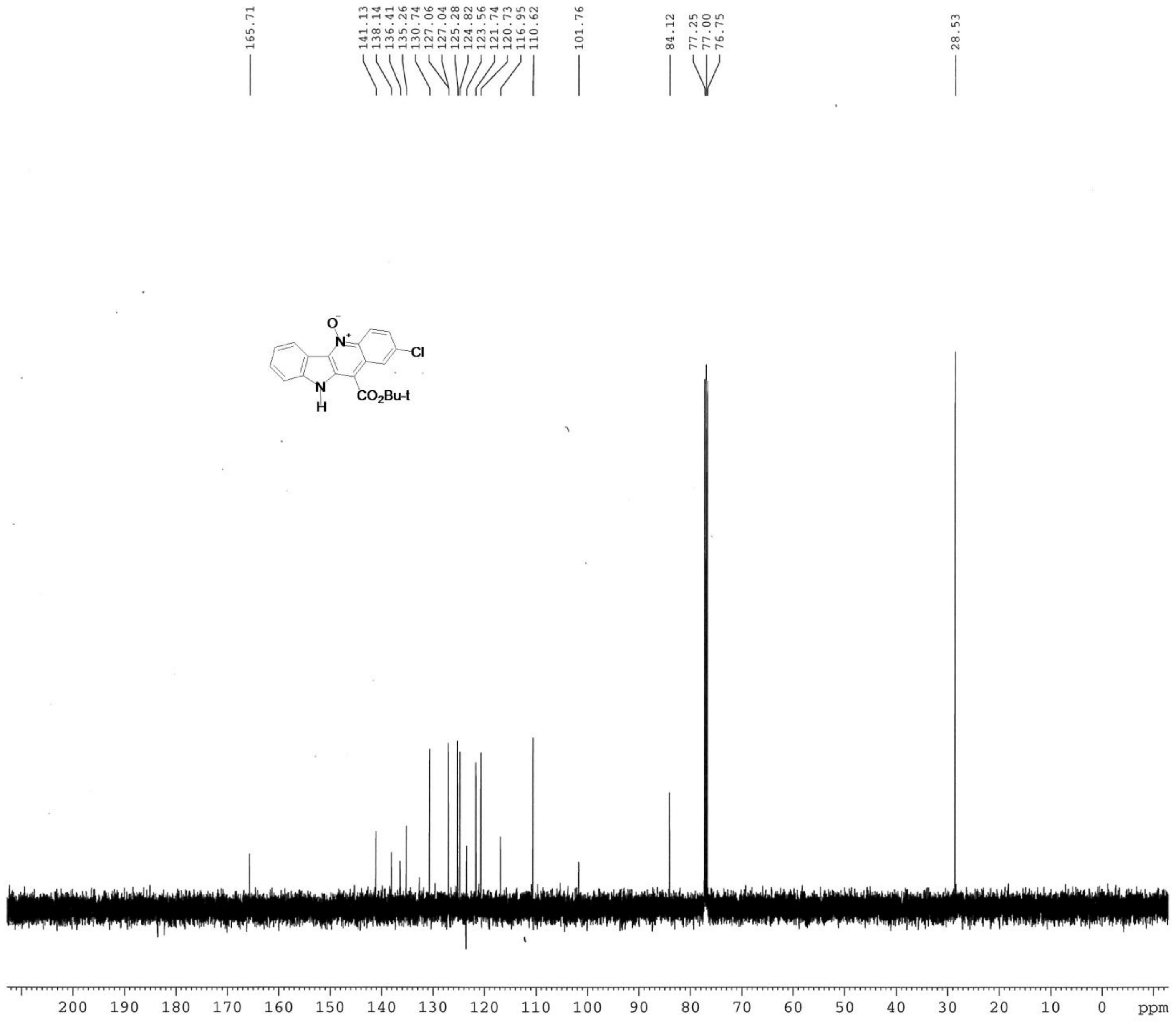

^{13}C NMR spectrum of *tert*-butyl 2-chloro-10*H*-indolo[3,2-*b*]quinoline-11-carboxylate-5-oxide (13).

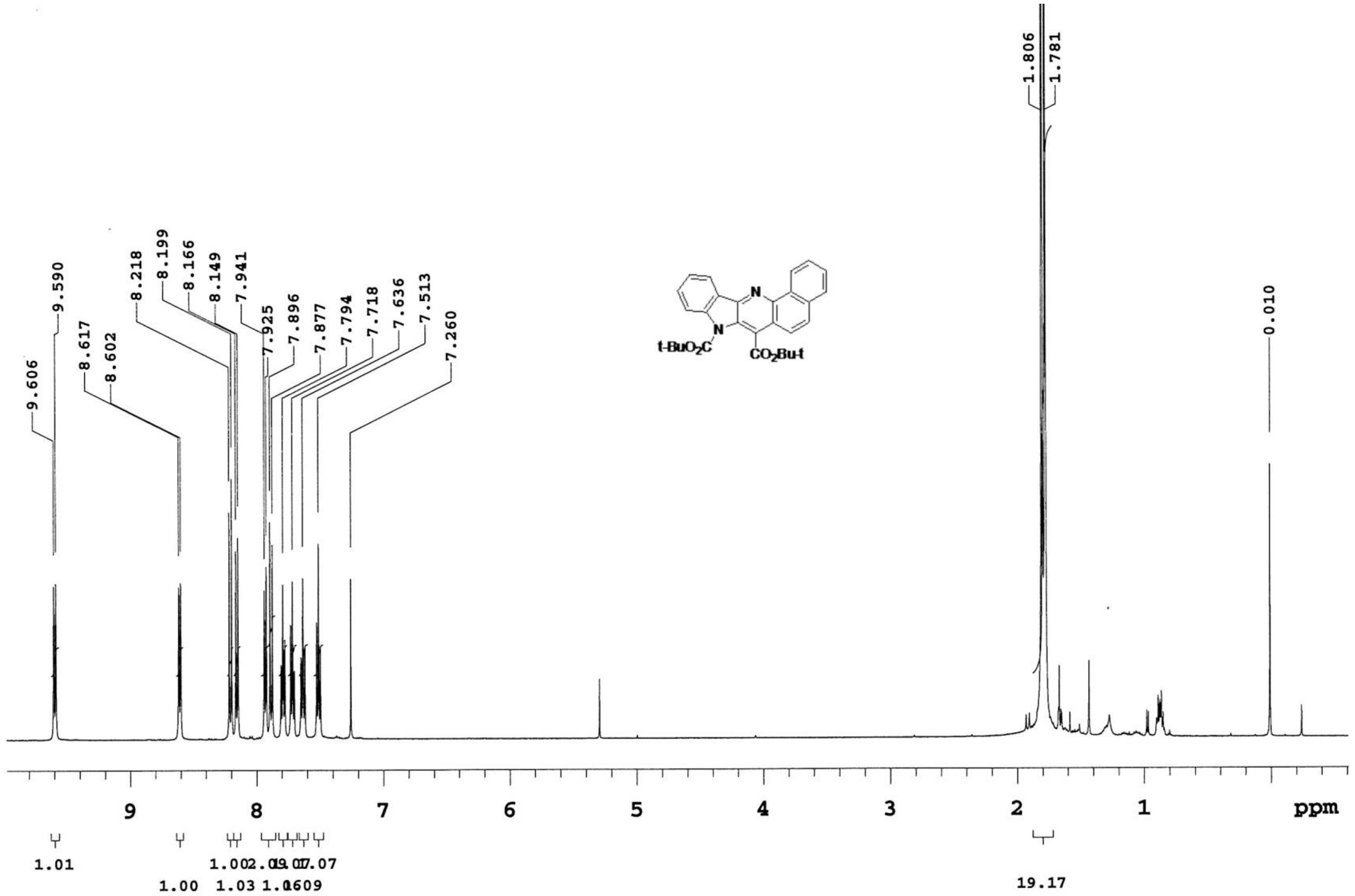

^{1}H NMR spectrum of bis(*tert*-butyl) 8*H*-benzo[*h*]indolo[3,2-*b*]quinoline-7,8-dicarboxylate (14).

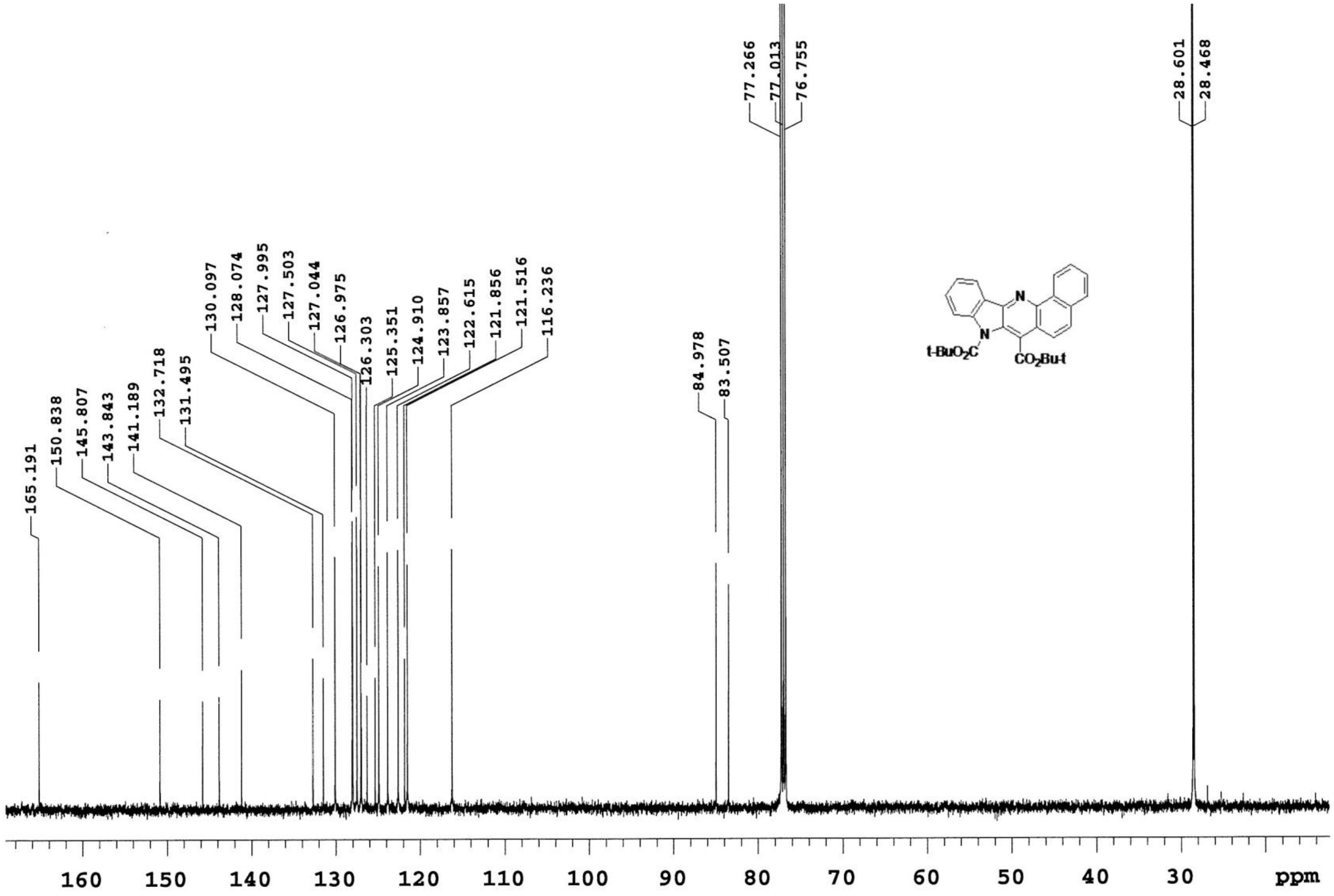

^{13}C NMR spectrum of bis(*tert*-butyl) 8*H*-benzo[*h*]indolo[3,2-*b*]quinoline-7,8-dicarboxylate (14).

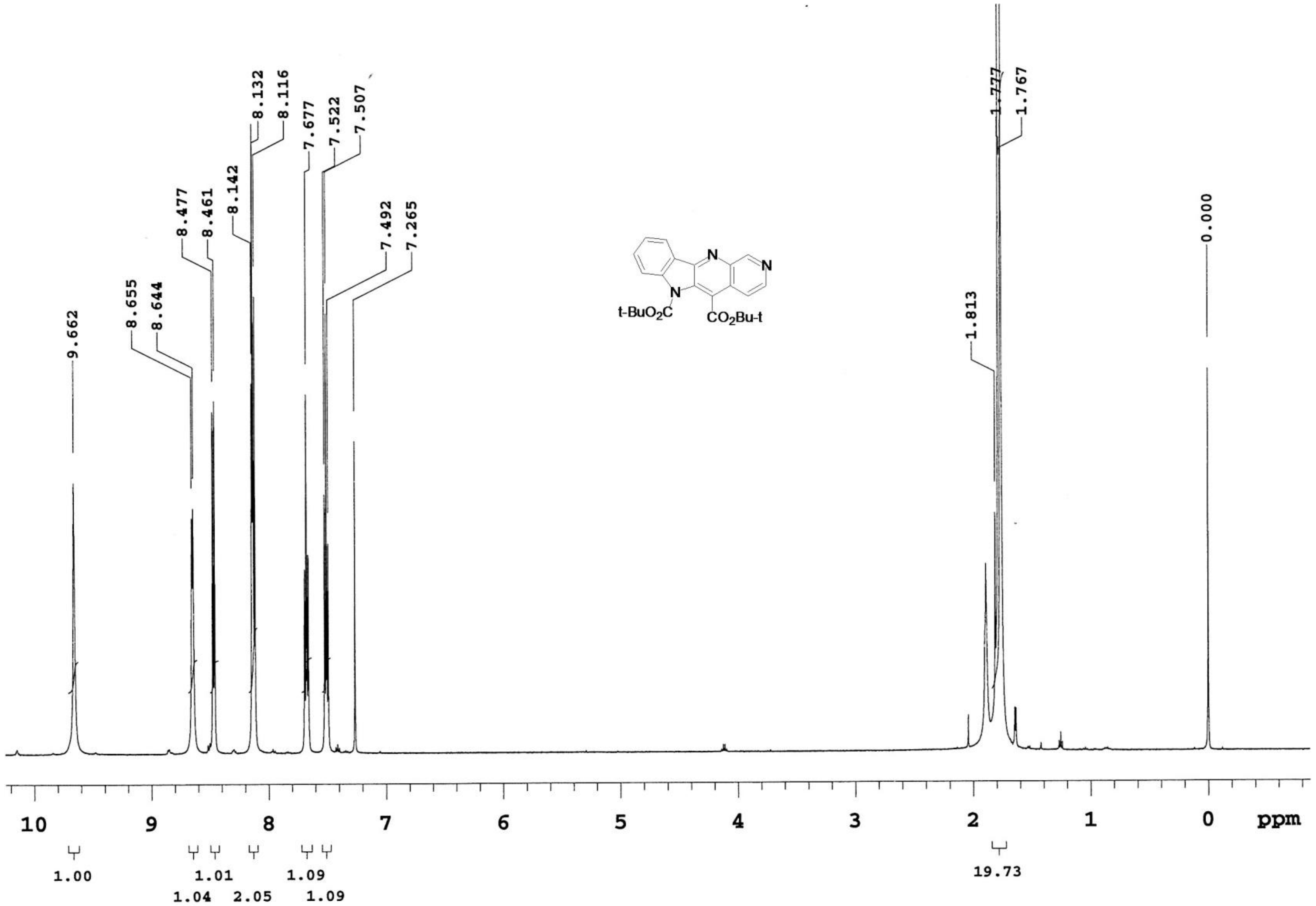

^{1}H NMR spectrum of bis(*tert*-butyl) 6*H*-indolo[3,2-*b*][1,7]naphthiridine-5,6-dicarboxylate (15).

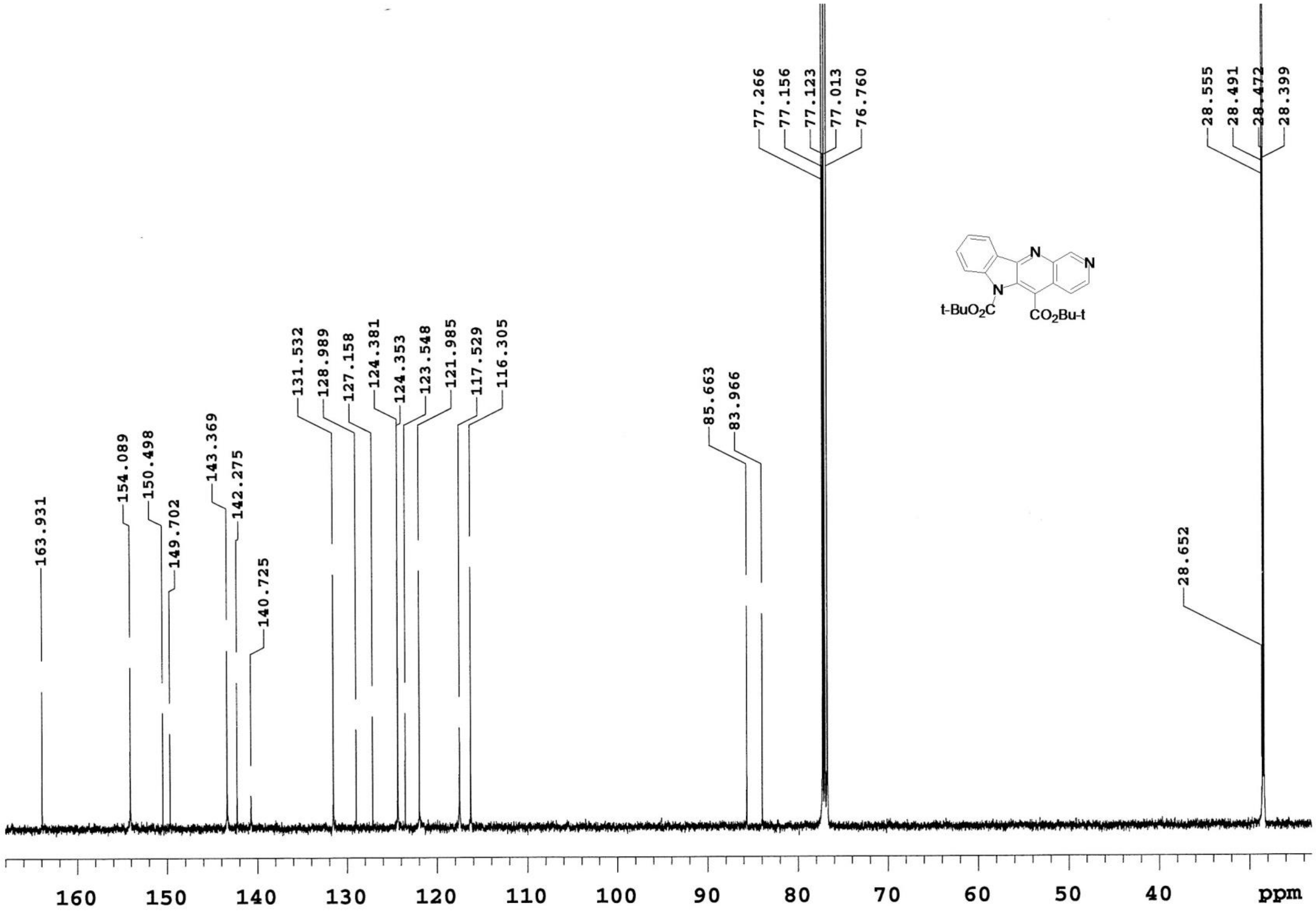

^{13}C NMR spectrum of bis(*tert*-butyl) 6*H*-indolo[3,2-*b*][1,7]naphthiridine-5,6-dicarboxylate (15).

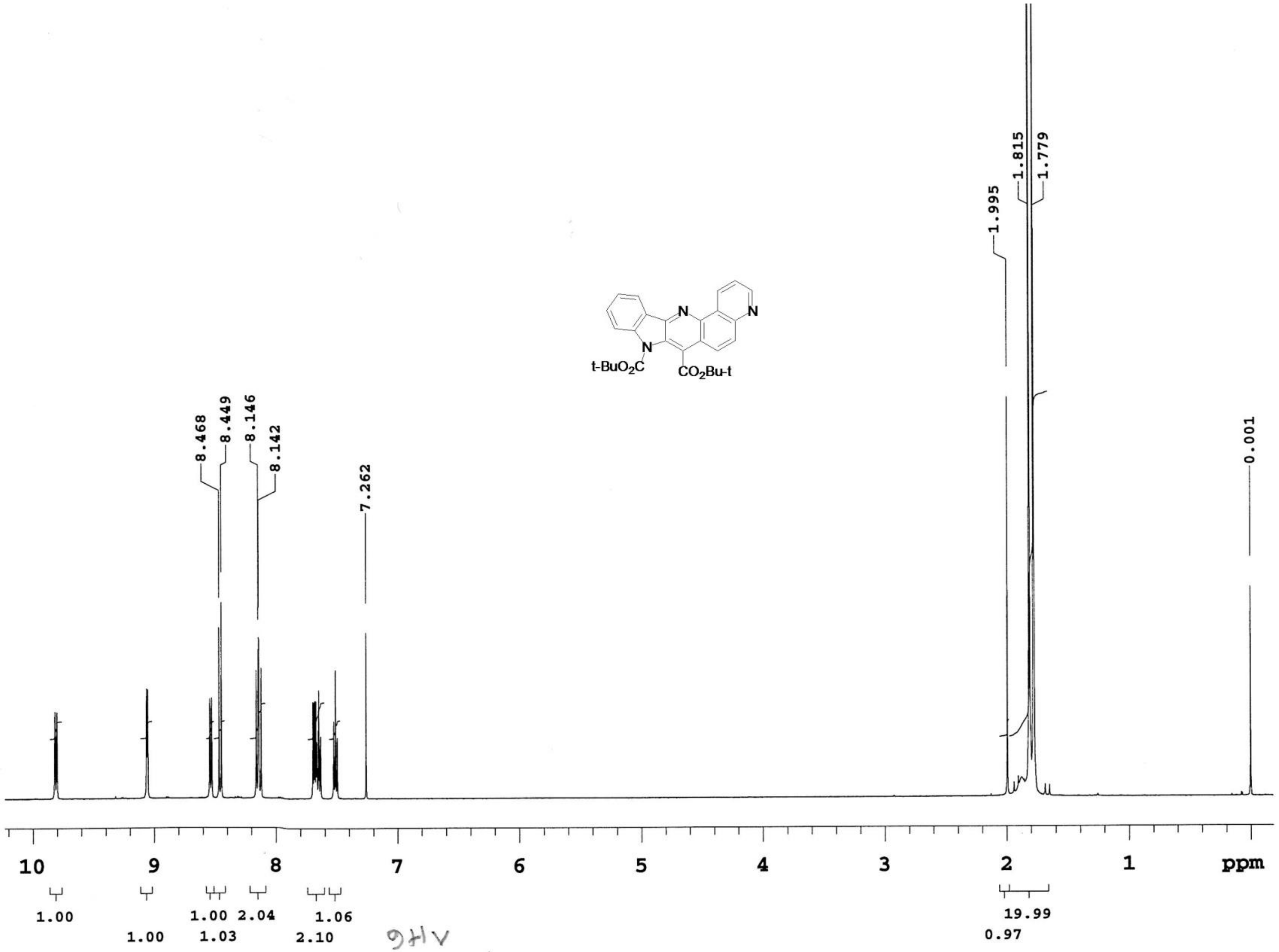

^{1}H NMR spectrum of bis(*tert*-butyl) 8*H*-indolo[3,2-*b*][1,7]phenanthroline-7,8-dicarboxylate (16).

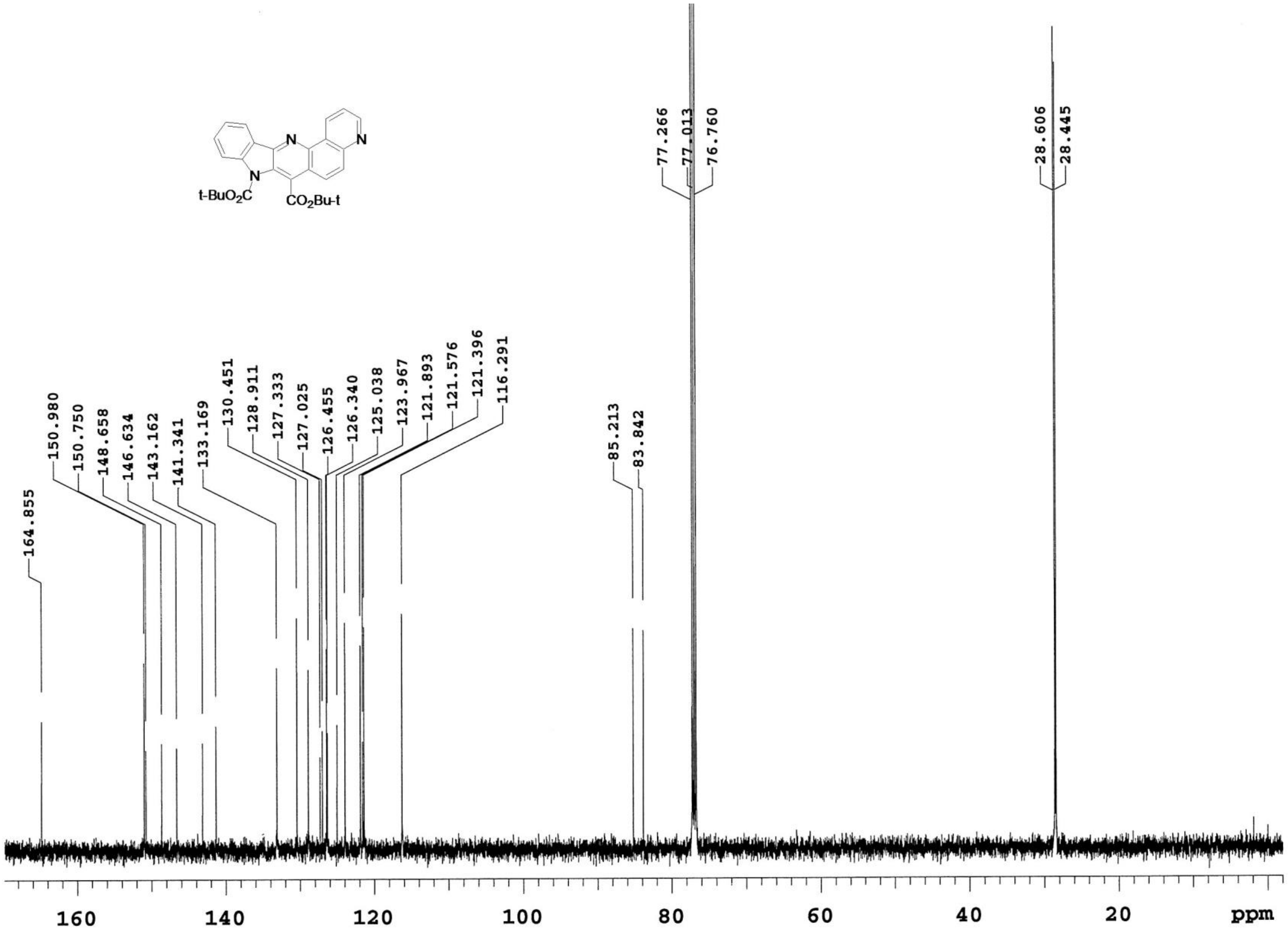

^{13}C NMR spectrum of bis(*tert*-butyl) 8*H*-indolo[3,2-*b*][1,7]phenanthroline-7,8-dicarboxylate (16).

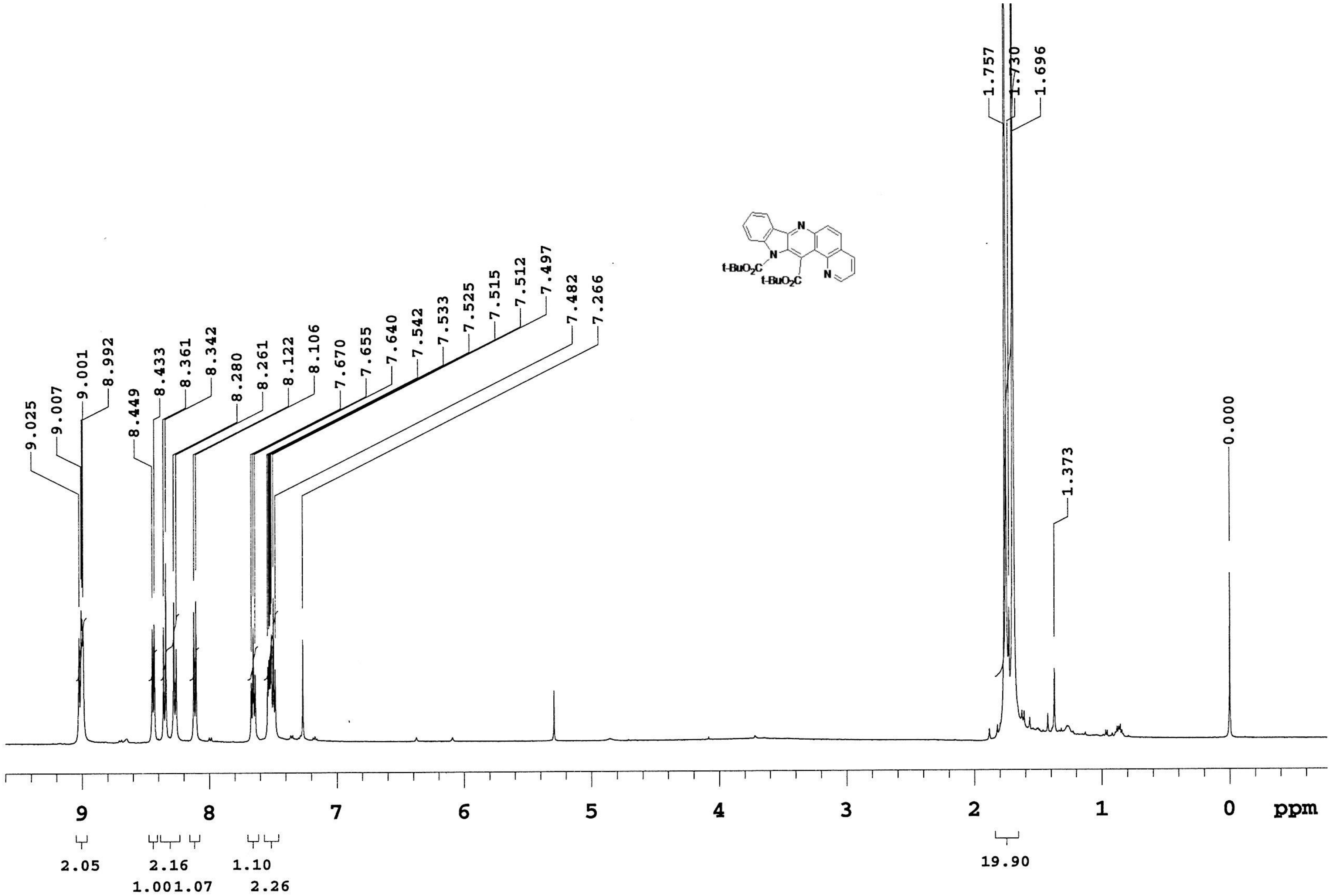

^{1}H NMR spectrum of bis(*tert*-butyl) 12*H*-indolo[3,2-*j*][1,7]phenanthroline-12,13-dicarboxylate (17).

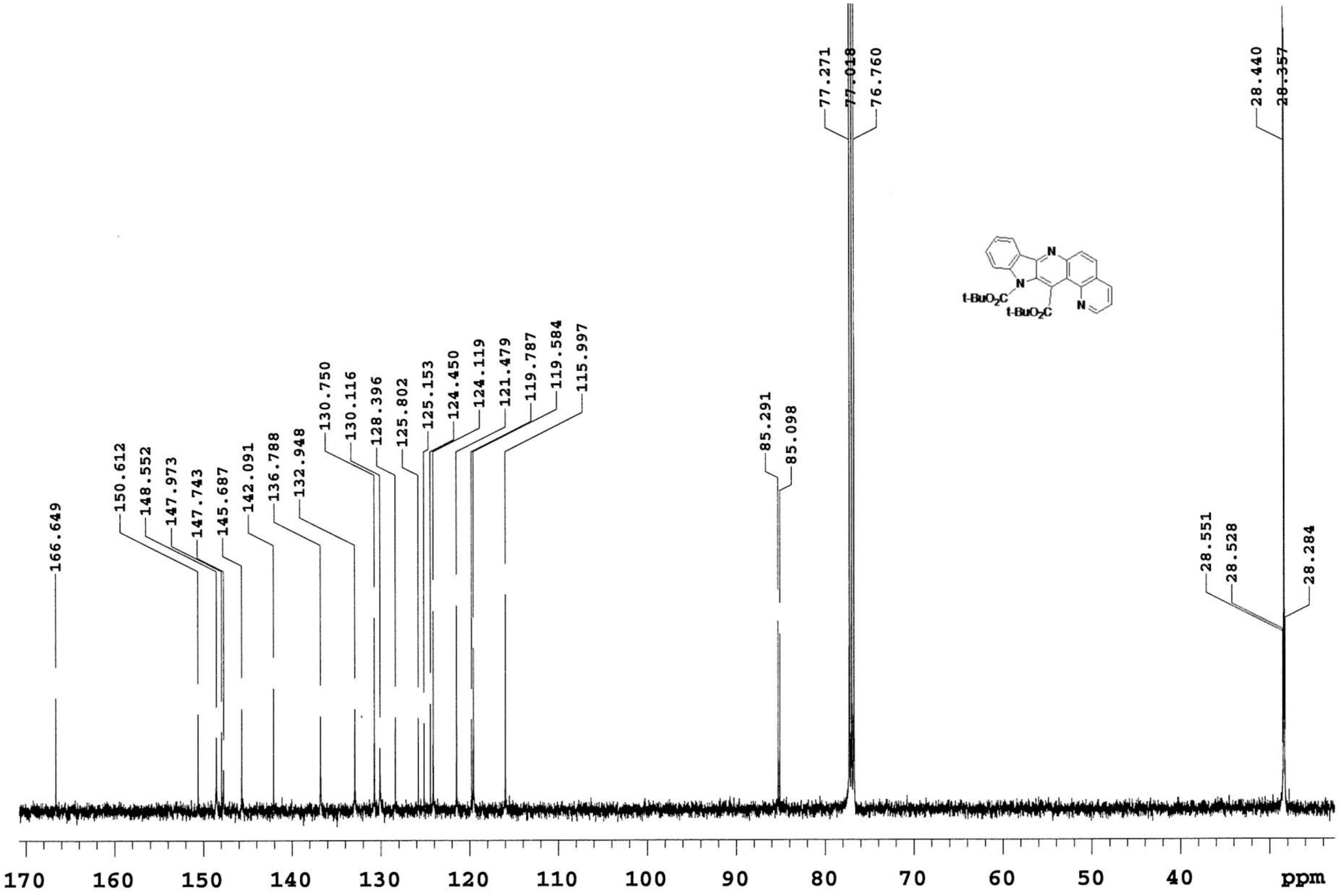

^{13}C NMR spectrum of bis(*tert*-butyl) 12*H*-indolo[3,2-*j*][1,7]phenanthroline-12,13-dicarboxylate (17).

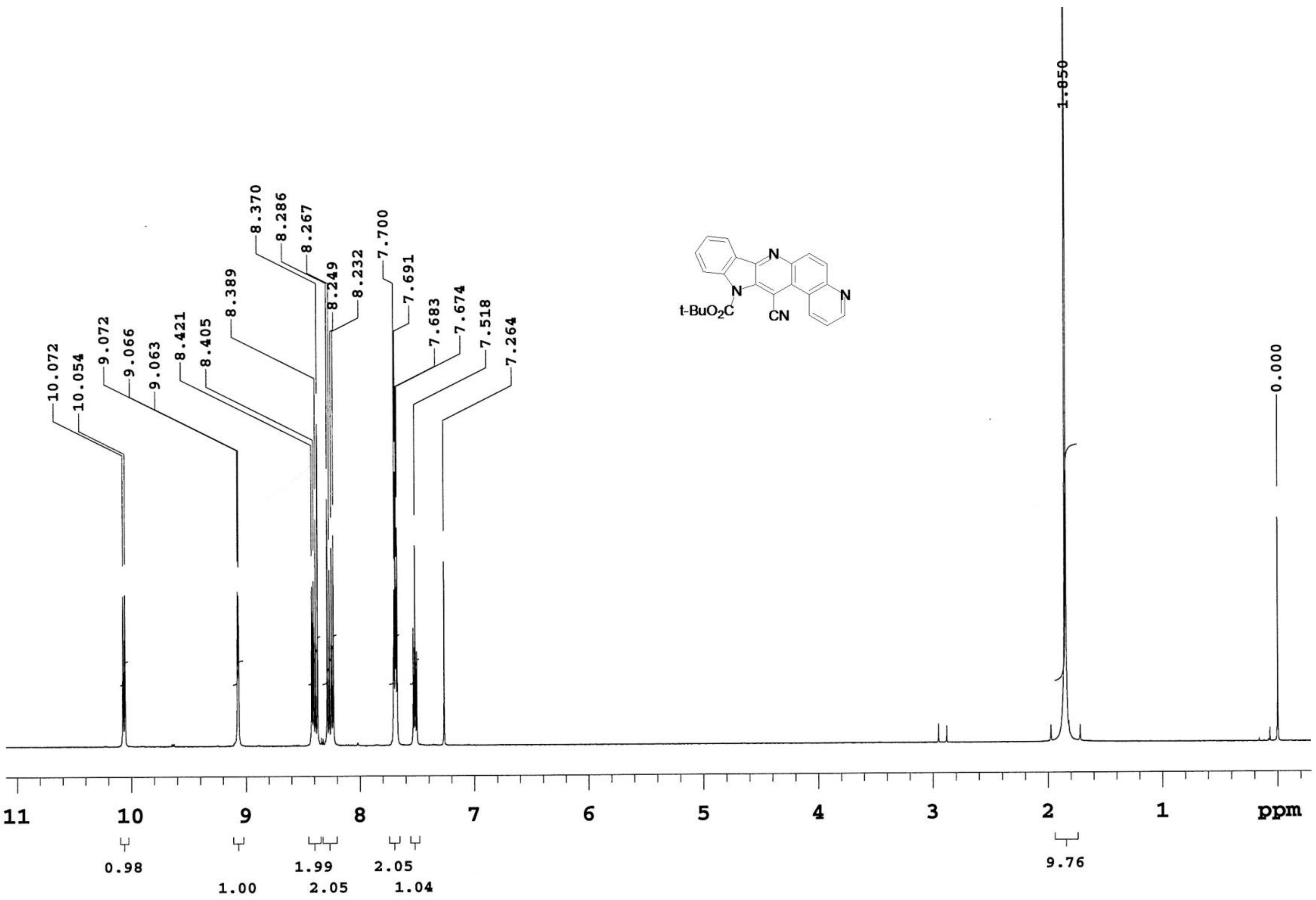

^{1}H NMR spectrum of 12 *tert*-butyl 13-cyano-12*H*-indolo[3,2-*b*][4,7]phenanthroline-12-carboxylate (18).

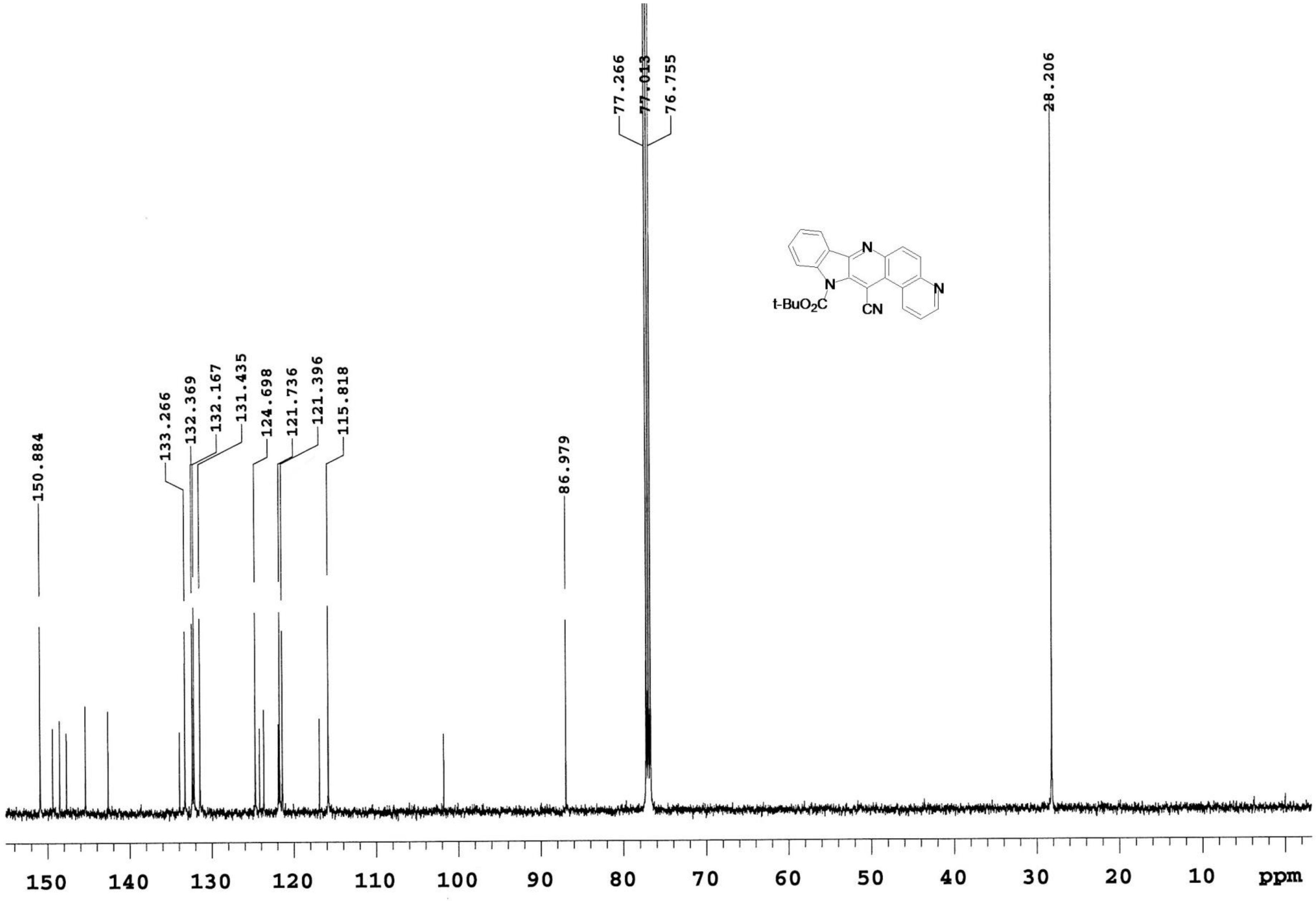

^{13}C NMR spectrum of *tert*-butyl 13-cyano-12*H*-indolo[3,2-*b*][4,7]phenanthroline-12-carboxylate (18).

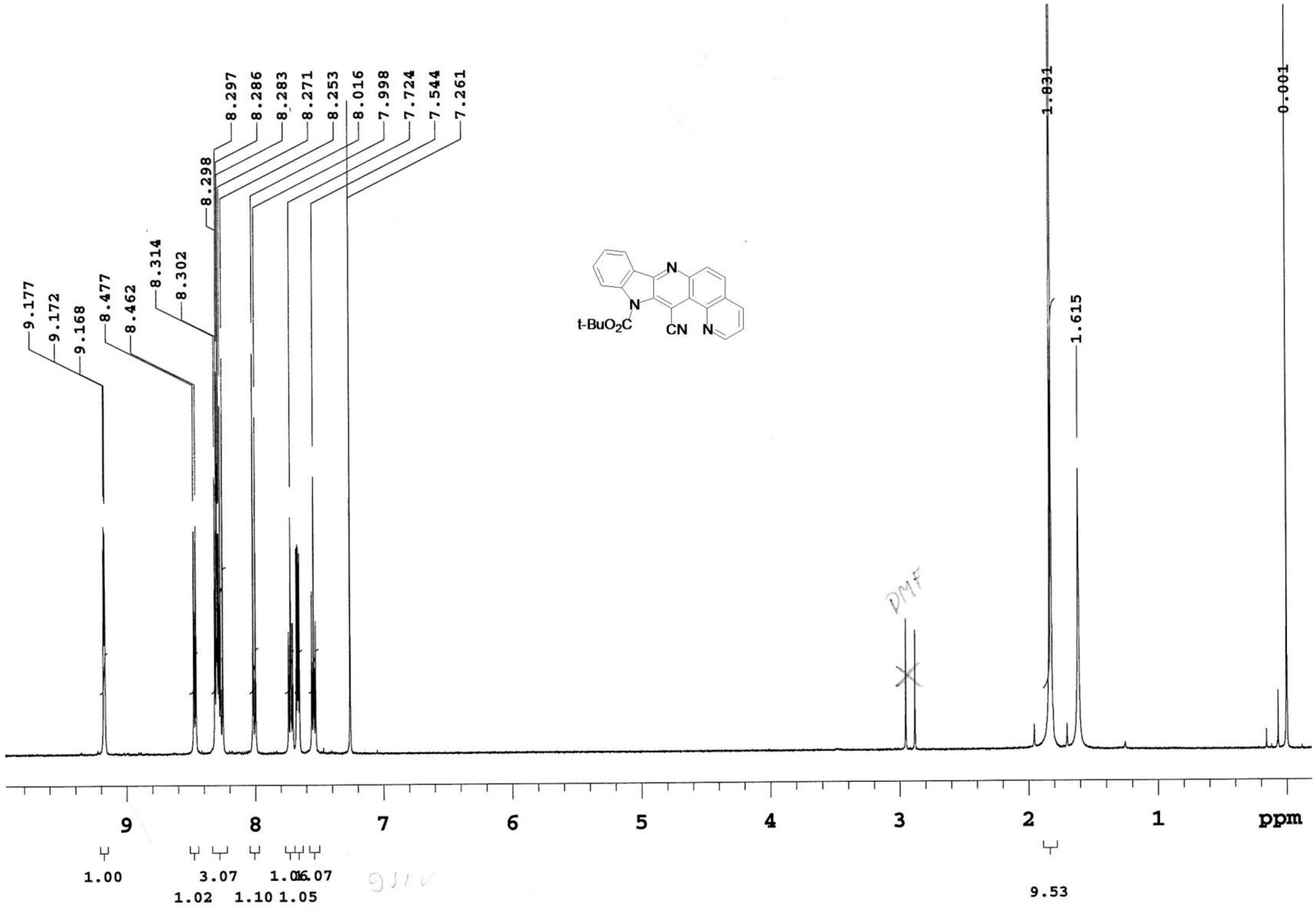

^{1}H NMR spectrum of *tert*-butyl 13-cyano-12*H*-indolo[3,2-*b*][1,5]phenanthroline-12-carboxylate (19).

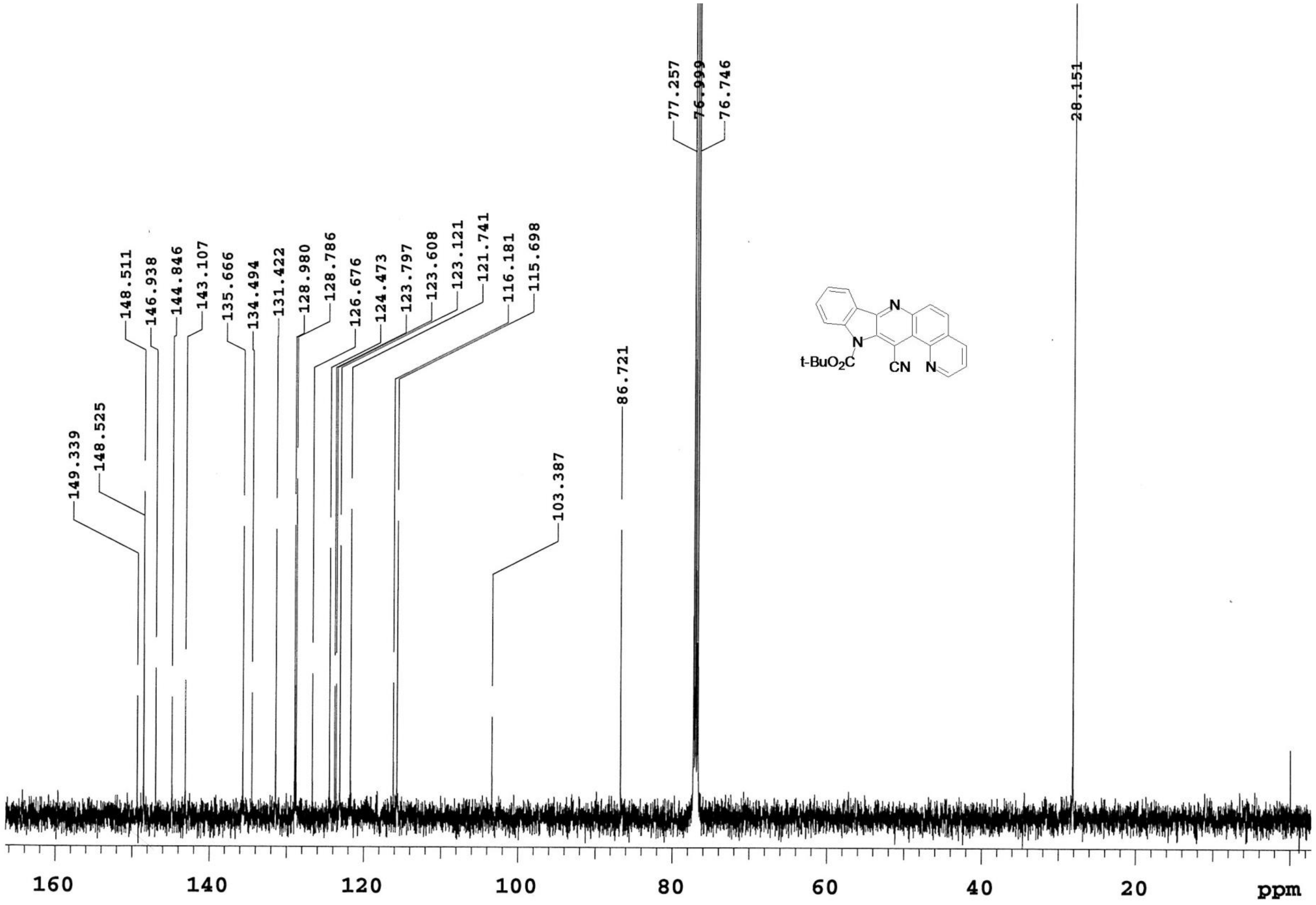

^{13}C NMR spectrum of *tert*-butyl 13-cyano-12*H*-indolo[3,2-*b*][1,5]phenanthroline-12-carboxylate (19).

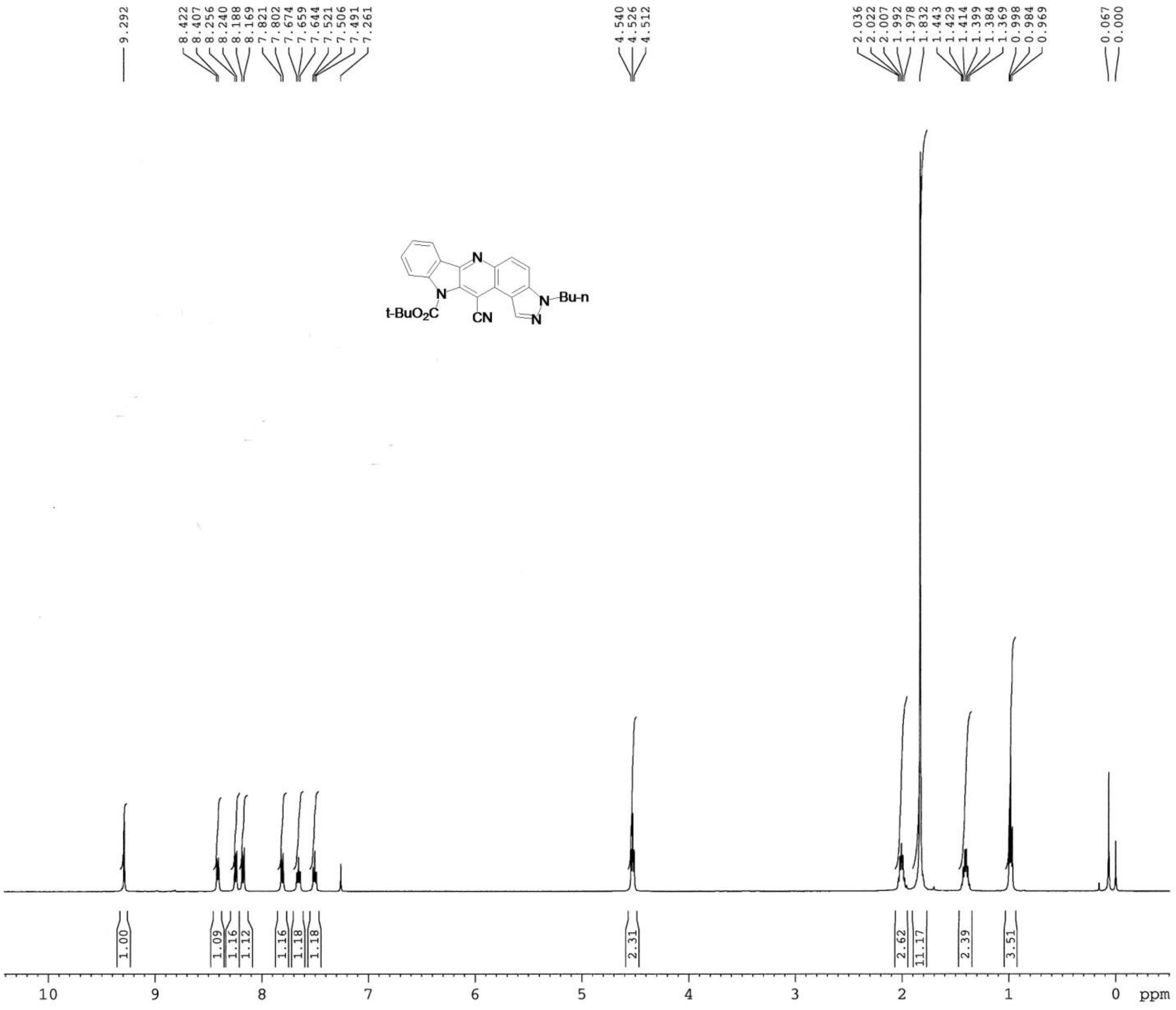

^{1}H NMR spectrum of *tert*-butyl 3-*n*-butyl-12-cyano-11*H*-indolo[3,2-*b*]pyrazolo[4,5-*f*]quinoline-11-carboxylate (20).

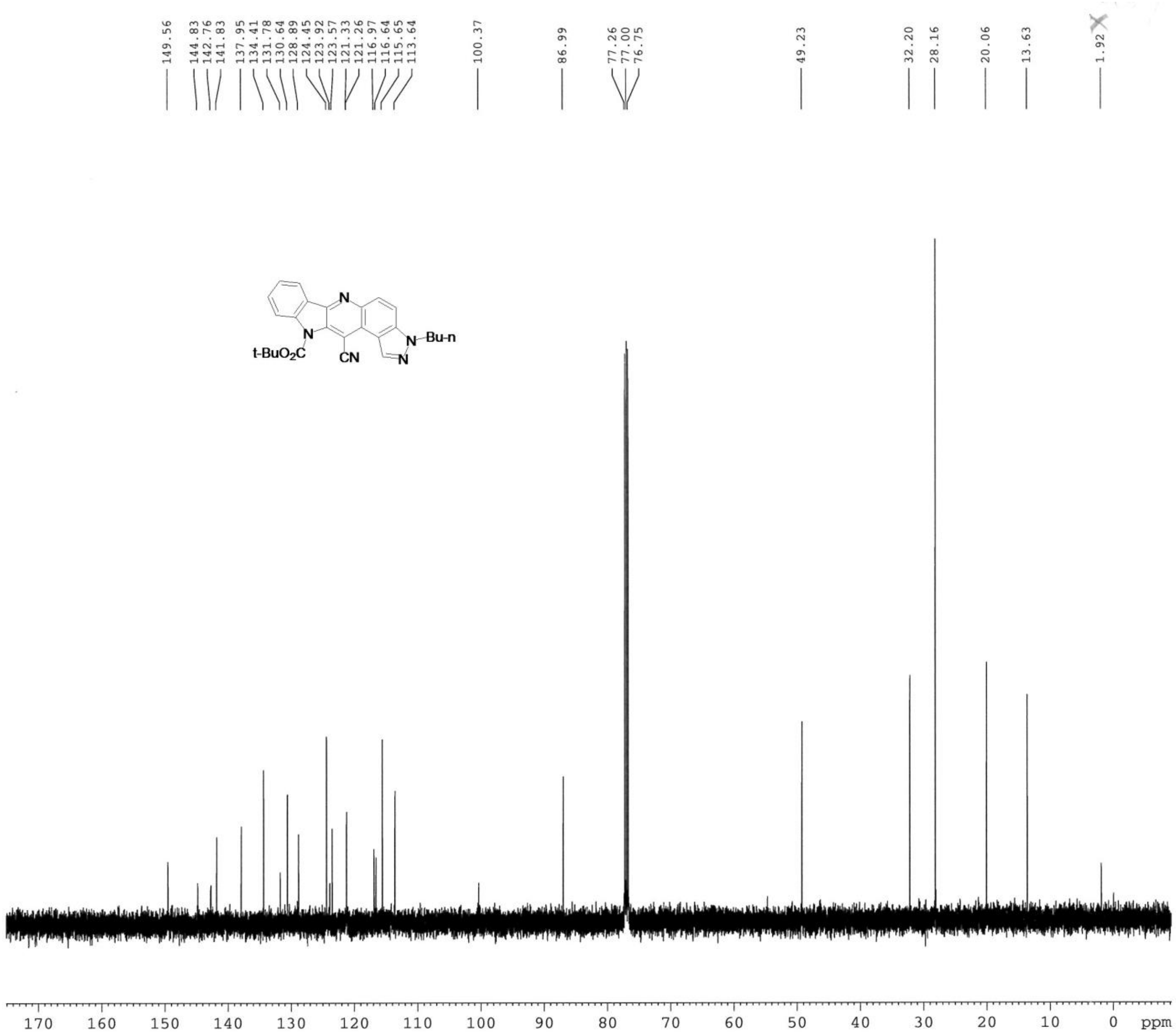

^{13}C NMR spectrum of *tert*-butyl 3-*n*-butyl-12-cyano-11*H*-indolo[3,2-*b*]pyrazolo[4,5-*f*]quinoline-11-carboxylate (20).
